# A qualitative systematic review and quality assessment of pharmacoeconomic evaluations on Chinese Herbal Medicine from 2020 to 2025

**DOI:** 10.3389/fpubh.2026.1738097

**Published:** 2026-02-17

**Authors:** YuRan Cai, Qing Tang, TengFei Lin, Dan Wang, Ming Hu, Nan Yang

**Affiliations:** 1 West China School of Pharmacy, Sichuan University, Chengdu, Sichuan, China; 2 Shenzhen Institute of Advanced Technology, Chinese Academy of Sciences, Shenzhen, Guangdong, China

**Keywords:** Chinese herbal medicine, economic evaluations, pharmacoeconomics, quality assessment, systematic review, traditional Chinese medicine

## Abstract

**Objective:**

This systematic review evaluated the status quo and quality of pharmacoeconomic (PE) evaluations of Chinese Herbal Medicine (CHM) published between 2020 and 2025, aiming to identify common quality flaws, compare the performance of different quality assessment tools, and formulate targeted recommendations for improving study quality.

**Methods:**

A comprehensive literature search was conducted across six databases to retrieve studies published in six years. Included studies were assessed using three validated tools: Consolidated Health Economic Evaluation Reporting Standards 2022 (CHEERS 2022), the British Medical Journal guidelines, and the Quality of Health Economics Studies (QHES) scales. Data regarding study characteristics were systematically extracted and assessed. Descriptive statistics were employed to analyze the study characteristics, while Intraclass Correlation Coefficients (ICC) and Bland-Altman analysis were applied to assess the differences in scores among the three tools.

**Results:**

A total of 198 studies were included. Of these, 58 studies (29.29%) failed to report the study perspectives, 148 studies (74.75%) adopted short-term time horizons (< 6 months), 2 studies with time horizons exceeding 1 year did not report discounting rates. 160 studies (80.81%) measured direct medical cost only. Outcome measures predominantly relied on conventional clinical efficacy rates (119 studies, 60.10%) rather than Traditional Chinese Medicine (TCM) syndrome indicators (25 studies, 12.63%). Incremental analyses was conducted in 155 studies (78.28%), and sensitivity analyses was performed in 161 studies (81.31%). Mean quality scores were 54.14% ± 9.16% for CHEERS 2022, 71.08% ± 9.41% for BMJ guidelines, and 68.83 ± 14.69 points for QHES scale. The overall ICC among the three tools was 0.580. Limits of agreement were as follows: CHEERS vs. BMJ were (−32.61%, −1.27%), CHEERS vs. QHES were (−39.12%, 9.75%), and BMJ vs. QHES were (−18.37%, 22.87%).

**Conclusion:**

While the quality of CHM PE evaluations has improved, substantial gaps remain in reporting transparency and methodological rigor, particularly regarding study protocols and CHM-specific outcome measures. The discrepancy in scores across assessment tools suggests that reporting quality does not always align with methodological soundness. Future research should strictly adhere to international guidelines (e.g., CHEERS), pre-register protocols, and adopt a multi-tool strategy for quality assessment to enhance the evidence base for decision-making.

**Systematic Review Registration:**

https://www.crd.york.ac.uk/PROSPERO/, identifier: CRD420251117386.

## 1 Introduction

Driven by advances in medical technology and social wealth accumulation, a critical global trend has emerged: healthcare expenditures are growing faster than household income (1, 2). This trend has been exacerbated in the post COVID-19 pandemic era, placing unprecedented financial strain on healthcare systems worldwide (3). According to the World Health Statistics 2025, substantial gaps persist in achieving Universal Health Coverage. Globally, 13.50% of the population spends over 10.00% of their household income on Out-Of-Pocket medical expenses, often leading to or exacerbating poverty (4). Against this backdrop, optimizing healthcare resource allocation and delivering high-quality health services has become a core and long-term challenge for policymakers seeking sustainable global health development (5).

Chinese Herbal Medicine (CHM) refers to therapeutic agents derived from plants, animals, and minerals, applied under the theoretical guidance of Traditional Chinese Medicine (TCM). CHM encompasses a broad spectrum of classifications, primarily categorized into raw medicinal materials and processed preparations. These preparations include traditional clinical forms (such as pills and decoction), Chinese patent medicines, and formula granules (6). While TCM offers a distinct therapeutic approach through its natural ingredients, holistic treatment, and syndrome differentiation (7), a significant challenge lies in the considerable overlap in the efficacy and indications of many TCM formulations (8). This overlap creates a need for evidence-based selection to identify the most clinically effective and cost-effective options. Such an approach is crucial for optimizing patient outcomes while alleviating their financial burden.

Pharmacoeconomics (PE) has progressively emerged as a critical decision-making tool since the 1970s (9). The first PE evaluation of CHM was published in 1997, initiating a subsequent, gradual expansion of related literature (10). However, concerns regarding the quality of this growing evidence base have persisted. Indeed, systematic reviews of CHM PE evaluations published prior to 2020 have consistently identified recurrent quality weaknesses, including poorly defined perspectives, small sample sizes, short time horizons, and an overall low quality (6, 11–13). While these findings underscore persistent challenges, it remains unclear whether more recent studies (from 2020 onward) have demonstrated meaningful improvement.

Beyond the need for an updated review, a critical methodological gap exists in the assessment of study quality. Previous systematic reviews have typically employed only one of the established quality assessment tool–either the Consolidated Health Economic Evaluation Reporting Standards 2022 (CHEERS 2022) (14), the British Medical Journal (BMJ) guidelines (15), or the Quality of Health Economics Studies (QHES) (16). Consequently, the field lacks a unified analysis that simultaneously leverages the strengths of all three tools and compares their results, which is essential for understanding potential assessment biases and establishing a more definitive judgment of study quality.

To address these gaps, the scope of this study was strictly limited to PE evaluations of CHM, and aims to characterize the current research landscape and to assess the reporting and methodological quality of the included studies by concurrently applying three validated tools. Our ultimate goal is to provide evidence-based guidance for enhancing the rigor of future studies.

## 2 Methods

This systematic review was conducted in accordance with the Preferred Reporting Items for Systematic Reviews and Meta-Analyses (PRISMA, 2020) guidelines (17), and registered on the International Prospective Register of Systematic Reviews (PROSPERO) under registration number CRD420251117386.

### 2.1 Search strategy

Literature retrieval for this study was conducted across multiple databases, including China National Knowledge Infrastructure (CNKI), VIP Database (VIP), Wanfang Database (WF), PubMed, Embase, and the Cochrane Library, covering the period from January, 2020 to December, 2025. To ensure exhaustive coverage, the search strategy employed a combination of subject headings and free-text terms.

The search string combined economic evaluation terms (e.g., “Pharmacoeconomics,” “Cost-Effectiveness Analyses,” “Cost-Utility Analyses,” “Markov Model,” “Decision Tree,” and “Partitioned Survival Model”) with a set of intervention terms. The intervention keywords encompassed two dimensions: (i) general CHM categories (e.g., “Chinese Medicine” OR “Traditional Chinese Medicine” OR “Chinese Herbal Pieces” OR “Chinese Patent Medicine”); (ii) specific dosage forms in CHM appearing in titles (e.g., “Granule,” OR “Pill,” OR “Oral Liquid,” OR “Decoction,”). Detailed search strategies for each database are presented in Supplementary Table 1.

### 2.2 Inclusion and exclusion criteria

The inclusion criteria were: (i) Studies published by established content experts in peer-reviewed journals; (ii) Focusing on CHM, including Chinese Patent Drug, Chinese Herbal Decoction Pieces, Chinese medicinal materials, or combined Chinese medicine therapies.

Exclusion criteria were: (i) Non-CHM therapies (e.g., acupuncture, gua sha, or tui na massage); (ii) Other traditional medicine studies not guided by TCM theory, such as Traditional Korean Medicine (TKM) or Korean Herbal Medicine (KHM); (iii) Non-PE empirical studies such as theoretical/methodological studies, reviews, news/commentary articles, budget impact analysis, or study protocols etc; (iv) Health technology assessments or clinical comprehensive evaluation studies that directly cite others' conclusions without conducting PE assessments or economic evaluations; (v) Unpublished works or gray literature including conference proceedings or abstracts were also excluded.

### 2.3 Study selection

After completing the literature search according to the established retrieval strategy, the results were batch imported into EndNote 21 reference management software. Duplicate records were removed through a combination of EndNote 1's automated duplication check and manual review.

Literature screening was conducted independently by two researchers (Cai and Tang) and cross-checked. Discrepancies in their Literature screening were resolved through discussion or consultation by a third researcher (Yang). Preliminary screening was performed by reviewing article titles and abstracts based on the inclusion and exclusion criteria, with final selection determined after reading the full texts.

### 2.4 Data extraction

A 23-item extraction form was designed for this study. Extracted content included: (i) Basic information related to the included studies (title, country or region, authors and affiliations, journal, funding sources, disease type, drug name), (ii) Study characteristics of PE (types of study design, model, perspective, intervention, evaluation method, time horizon, sample size, cost scope, health outcome, discounting rate, incremental analysis, threshold, sensitive analysis type and variable, and limitations analysis).

### 2.5 Quality assessments

In this study, three tools, i.e., CHEERS 2022, BMJ, and QHES, were employed for the quality assessment of the included studies, allowing for a distinct evaluation of both reporting and methodological quality. Among these, the CHEERS 2022 checklist, widely recognized within the field, prioritizes the standardization of reporting by the clarity and comprehensiveness of study design, results, and uncertainty analyses (18). The BMJ guidelines, representing the earliest and most frequently cited framework for PE, serve as a standard for peer review; aims to evaluate reporting completeness with a focus on logical rationale, data reliability, and internal validity. In contrast, the QHES scale, developed by Chiou et al. (16, 19), integrates core methodological elements using a quantitative weighting system, by assigning distinct weights to 16 items—such as data derivation, analytical robustness, incremental analyses, and subgroup analyses. Scores were calculated for included studies based on the respective items from these three quality assessment tools.

Given that no widely accepted quality assessment thresholds have yet been established, this study established a literature quality grading system by referencing methodologies from existing literature. When scoring using CHEERS 2022 and BMJ, “Fully Compliant,” “Partially Compliant,” “Applicable but not Compliant” or “Not Reported,” were respectively assigned with score “1,” “0.5” and “0,” and items for “not applicable” will not be counted toward the score. In order to determine both overall compliance rates and items compliance rates for each key evaluation techniques, the quality level of the included studies was calculated as the percentage of the actual score relative to the total possible score for applicable items (maximum of 100.00%). Finally, quality grades were assigned based on the compliance rate, and studies with compliance rates ≥ 85.00%, 85.00% > compliance rates ≥ 70.00%, 70.00% > compliance rates ≥ 55.00%, and compliance rates < 55.00% were rated as “High,” “Moderate,” “Poor,” and “Extremely Poor,” respectively (18, 20–22).

With the calculation of the quality score for each study (maximum of 100.00 points), the QHES scale used the evaluation criteria of “Yes” for “Compliant,” and “No” for 'Not Reported'. Studies were categorized into four categories of quality based on the total scores: High (score: 75.00–100.00), Moderate (score: 50.00–74.00), Poor (score: 25.00–49.00), and Extremely Poor (score: 0.00–24.00), respectively (6, 23–25).

Two reviewers (Cai and Tang) assessed the quality of the included studies and independently scored each item across assessment tools. Discrepancies in their scores were resolved through re-examination of the original texts and consultation. If consensus could not be reached through consultation, a third reviewer (Yang) conducted a re-evaluation.

### 2.6 Statistical analyses

We calculated the Mean and Standard Deviation to describe the central tendency and dispersion of the overall data for literature quality scores. And the heatmap was used to demonstrate the quality assessment result among three tools. To comprehensively evaluate the consistency of results between tools, we employed both the Intraclass Correlation Coefficient (ICC) and Bland-Altman analyses. While ICC assesses relative reliability, the Bland-Altman method was used to quantify the systematic bias (Mean Difference) and the 95% limits of agreement (LoA) between the instruments, and we using the criteria that values between 0.5 and 0.75 show moderate reliability, values between 0.75 and 0.9 show good reliability, and >0.90 show excellent reliability (26). (This study normalized QHES scores given that both CHEERS 2022 and BMJ quality assessment results were presented as compliance rates. Specifically, scores were converted to compliance rates using the equation of “Compliance Rate = Actual Study Score/Maximum Scale Score × 100.00%.”).

Statistical significance established at a *p*-value less than 0.05 and 95% confidence intervals (95% CI) used to indicate the precision of the estimates. Descriptive analysis of the extracted data was completed using Excel 2021. SPSS 27.0 was used to calculate ICC, and Python software was used to draw Bland-Altman plots and Heatmap.

### 2.7 Subgroup and sensitivity analyses

To investigate potential sources of heterogeneity in quality, we conducted prespecified subgroup analyses based on publication language (Chinese vs. English) and indexing databases (CNKI, WF, VIP, PubMed, Embase, and Cochrane Library). Note that studies could be indexed in multiple databases; thus, groups were not mutually exclusive. Within each subgroup, we calculated descriptive statistics (Mean ± SD) for the CHEERS 2022, BMJ, QHES scores, the ICC value and performed Bland-Altman analyses for all three pairwise comparisons.

In addition, to assess the robustness of our findings, a sensitivity analyses was performed by excluding studies that lacked a clear description of the specific CHM drug interventions. We compared the quality as assessment results (CHEERS 2022, BMJ, and QHES) of this filtered dataset against the original full dataset using independent samples *t*-tests.

## 3 Results

**Figure 1:**
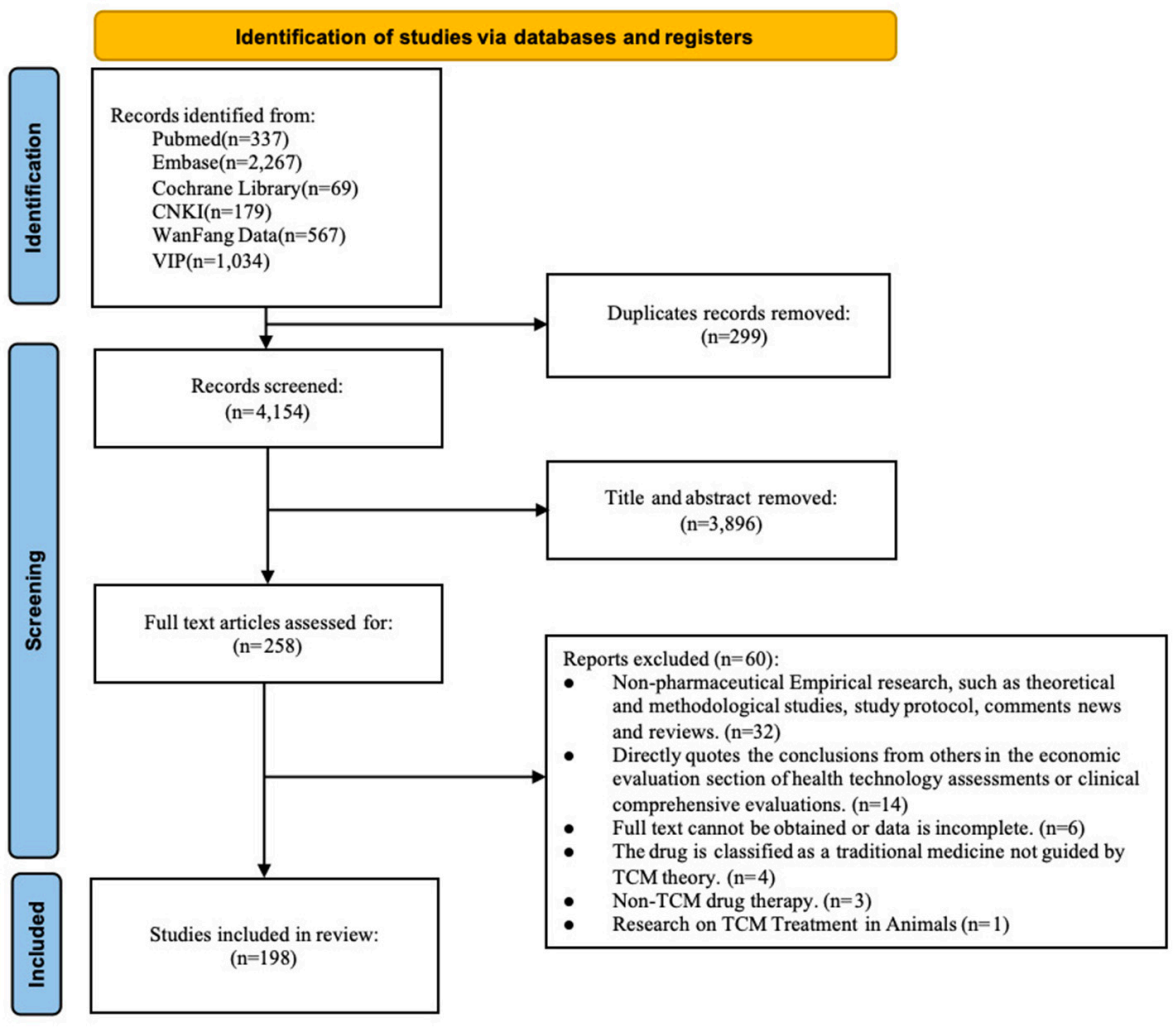
PRISMA flow diagram of the literature retrieval process.

According to the preset retrieval strategy, the initial search yielded 4,453 literatures sourced from PubMed (*n* = 337), Cochrane Library (*n* = 69), Embase (*n* = 2,267), WF (*n* = 567), VIP (*n* = 1,034), and CNKI (*n* = 179), respectively. After stepwise screening, 198 studies were ultimately included, comprising 178 in Chinese and 20 in English. The process of literature retrieval was illustrated in Figure 1, and an overview of the study characteristics is presented in Supplementary Table 2, Tables S1–S3.

### 3.1 Basic information of included studies

#### 3.1.1 . Study characteristics of included studies

All of the included studies originated from China, except four studies referenced data from non-Chinese populations, the remaining studies were conducted exclusively on Chinese patients. The identified studies were published on 65 journals, comprising 50 Chinese-language journals and 15 English-language journals. Among Chinese journals, the highest number of studies was published in *China Journal of Pharmaceutical Economics* (57 studies, 28.79%), followed by *Evaluation and analyses of drug-use in hospitals of China* (18 studies, 9.09%). Among English-language publications, *Frontiers in Pharmacology* hosted the most studies (4 studies, 2.02%). The affiliations of the first authors included hospitals, enterprises, research institutions, and universities, with 85.35% of the studies originating from hospitals and universities (169 studies). In addition, funding sources were specified in 101 studies (51.01%), except for 2 study funded by a company, all other studies were funded by organizations or governments.

As shown in Table 1, regarding study design, model-based studies were slightly more prevalent than those based on individual-level data, with Decision Tree being the predominant types of models (74, 37.37%). Studies based on individual data primarily employs retrospective designs. Regarding the study perspective, the healthcare system perspective was the most frequently adopted, accounting for over 40.00% of the studies. Notably, a substantial proportion of the literature (nearly 30%) failed to explicitly specify the perspective used. The specific disease categories were determined based on the International Classification of Diseases, 11th Revision (ICD-11) code groupings (https://icd.who.int/browse11/l-m/en). Consequently, the enrolled studies addressed treatments for 16 disease categories, with circulatory system diseases constituted the largest category, representing more than a quarter of all included studies, followed by infectious or parasitic diseases and neurological disorders, together, these top three categories accounted for more than half of the total studies. In terms of treatment comparisons, the evaluations were generally divided between using CHM as a monotherapy and as a combination therapy. For monotherapy, comparisons between different CHM regimens were the most common (56, 27.18%), while for combination therapies, the integration of CHM with conventional treatment vs. conventional treatment alone was the leading comparison type. Except for 7 studies that did not explicitly specify the drug names, substituting them “CHM Formulations” instead, and the remaining 191 studies involved a total of 225 CHM drugs in economic evaluations. When classifying the drugs involved by administration route, gastrointestinal tract administration was the primary delivery method, while injections also represented a notable share of the evaluated drugs. Among these, 174 drugs (77.33%) were included in China's National Reimbursement Drug List for Basic Medical Insurance (2025 Version) (NRDL) (27), with 47 (20.89%) classified as Category A and 127 (56.44%) as Category B. In addition, 63 (28.00%) types were listed in China's National Essential Medicines List (2018 Version) (28). Additionally, eight of these drugs were launched in China after 2020. The time horizons for these evaluations were generally short-term, a significant number of studies were conducted over a period of 14 days or less, and the majority concluded within six months (148, 74.75%). Sample sizes were falling in the range of 40 to 13,848, and 45 studies (22.73%) did not report sample size. Meanwhile, all the 198 studies reported the methods for economic evaluations. Over 65.00% employed Cost-effectiveness Analyses (CEA), 20 studies (10.10%) utilized combined analyses. Most of the studies considered direct costs only, with more than 80.00% of these further limited to direct medical costs. Indirect costs were incorporated in 14.65% of the studies, while only one study included intangible costs. Three studies did not specify the cost types used. Sensitivity analyses were performed in 81.31% of the studies, among these, 74 studies carried out DSA only, one conducted PSA only, and 84 studies performed both DSA and PSA. Among the studies performing DSA, 44 studies analyzed only cost parameters, 2 evaluated only health outcome parameters, and 9 studies with no sensitivity variables specified.

**Table 1:** Study characteristics for the included studies.

**Study characteristics**	***N* (%)**
**Study design (*****N*** = **198)**	
**Model-based study**	108 (54.55)
Decision Tree	74 (37.37)
Markov	28 (14.14)
Decision Tree + Markov	4 (2.02)
Partition Survival	1 (0.51)
Unspecified Model Type	1 (0.51)
**Individual-level data-based study**	90 (45.45)
**Prospective study**	27 (13.64)
Prospective Observational Study	17 (8.59)
Prospective RCT	10 (5.05)
**Retrospective study**	63 (31.82)
**Research perspective (*****N*** = **198)**	
Healthcare System Perspective	84 (42.42)
Societal Perspective	31 (15.66)
Healthcare Payer Perspective	12 (6.06)
Healthcare Provider's Perspective	8 (4.04)
Patient's Perspective	4 (2.02)
Societal Perspective + Healthcare System Perspective	1 (0.51)
Not Mentioned	58 (29.29)
**Disease classification (*****N*** = **198)**	
Circulatory System Diseases	55 (27.78)
Infectious or Parasitic Diseases	31 (15.66)
Neurological Diseases	20 (10.10)
Musculoskeletal or Connective Tissue Disorders	19 (9.60)
Respiratory System Diseases	16 (8.08)
Genitourinary System Diseases	16 (8.08)
Digestive System Diseases	12 (6.06)
Endocrine, Nutritional, or Metabolic Disorders	8 (4.04)
Tumors	6 (3.03)
Sleep Disorders	3 (1.52)
Pregnancy, Childbirth, and Postpartum Disorders	3 (1.52)
Skin Diseases	2 (1.01)
Diseases of the Eye and Adnexa	2 (1.01)
Mental, Behavioral, or Neurodevelopmental Disorders	2 (1.01)
Circulatory System Diseases + Neurological Diseases	1 (0.51)
Other (Diseases of the Ear or Mastoid Process; Injuries, Poisoning, or Consequences of External Causes)	2 (1.01)
**Treatment comparison (*****N*** = **206)**^a^	
**CHM used as monotherapy**	94 (45.63)
Comparing different types of CHM	56 (27.18)
CHM vs. Western Medicine	19 (9.22)
CHM vs. Placebo	9 (4.37)
CHM vs. Conventional Treatment	7 (3.40)
CHM vs. CHM vs. Western Medicine	2 (0.97)
CHM vs. CHM vs. Placebo	1 (0.49)
**CHM used as combination therapy**	112 (54.37)
CHM + Conventional Treatment vs. Conventional Treatment	47 (22.82)
CHM + Western Medicine vs. Western Medicine	34 (16.50)
CHM + Conventional Treatment vs. CHM + Conventional Treatment	15 (7.28)
CHM + Western Medicine vs. CHM + Western Medicine	6 (2.91)
CHM + Placebo vs. CHM + Placebo	2 (0.97)
CHM + Conventional Treatment vs. Conventional Treatment + Placebo	2 (0.97)
CHM + Conventional Treatment vs. Western Medicine + Conventional Treatment	2 (0.97)
CHM + Western Medicine + Conventional Treatment vs. Western Medicine + Conventional Treatment	1 (0.49)
CHM + Conventional Treatment vs. CHM + Conventional Treatment vs. No-treatment	1 (0.49)
CHM + Placebo vs. Western Medicine + Placebo	1 (0.49)
CHM + Western Medicine vs. CHM	1 (0.49)
**Drugs (*****N*** = **225)**	
Gastrointestinal Tract Administration	177 (78.67)
Injection Administration	43 (19.11)
Topical Administration	5 (2.22)
**Study time horizon (*****N*** = **202)**^b^	
≦14 d	56 (28.28)
15–30 d	31 (15.66)
1–3 m	41 (20.71)
4–6 m	20 (10.10)
7–12 m	11 (5.56)
>12 m	33 (16.67)
Not Specified	10 (5.05)
**Sample size (*****N*** = **198)**	
≦100	27 (13.64)
101–500	69 (34.85)
501–1,000	15 (7.43)
>1,000	42 (20.79)
Not Specified	45 (22.28)
**Analyzing method (*****N*** = **198)**^c^	
CEA	132 (65.84)
CUA	29 (14.85)
CMA	17 (8.59)
CEA + CUA	18 (9.09)
CEA + CMA	1 (0.51)
CEA + CBA	1 (0.51)
**Cost scope (*****N*** = **198)**	
Direct Medical Costs	160 (80.81)
Direct Medical Costs + Direct Non-Medical Costs + Indirect Costs	15 (7.58)
Direct Medical Costs + Indirect Costs	13 (6.57)
Direct Medical Costs + Direct Non-Medical Costs	6 (3.03)
Direct Medical Costs + Indirect Costs + Intangible Costs	1 (0.51)
Not Reported	3 (1.52)
**Incremental analyses (*****N*** = **198)**	
Yes	155 (78.28)
No	43 (21.72)
**Willingness-to-pay thresholds (*****N*** = **198)**	
Gross Domestic Product	57 (28.79)
Capita Disposable Income	31 (15.66)
Others	9 (4.55)
Not Reported	101 (51.01)
**Sensitivity analyses (*****N*** = **198)**	
One-Way Sensitivity Analyses + Probabilistic Sensitivity Analyses	82 (41.41)
One-Way Sensitivity Analyses Only	73 (36.87)
Multi-Way Sensitivity Analyses + Probabilistic Sensitivity Analyses	2 (0.99)
One-Way Sensitivity Analyses + Multi-Way Sensitivity Analyses + Probabilistic Sensitivity Analyses	1 (1.01)
One-Way Sensitivity Analyses + Multi-Way Sensitivity Analyses	1 (1.01)
Multi-Way Sensitivity Analyses Only	1 (1.01)
Probabilistic Sensitivity Analyses Only	1 (1.01)
Not Conducted	37 (18.69)
**Deterministic sensitivity analyses variables (*****N*** = **161)**^d^	
Drug Costs + Health Outcomes Indicators^**e**^	35 (21.74)
Drug Costs + Other Costs^**f**^ + Health Outcomes Indicators ^**e**^ + Other Variables ^**g**^	27 (16.77)
Drug Costs	26 (16.15)
Drug Costs + Other Costs^**f**^	19 (11.80)
Drug Costs + Other Costs^**f**^ + Health Outcomes Indicators^**e**^	19 (11.80)
Drug Costs + Health Outcomes Indicators ^**e**^ + Other Variables^**g**^	18 (11.18)
Drug Costs + Other Costs^**f**^ + Other Variables^**g**^	3 (1.86)
Drug Costs + Other Variables^**g**^	2 (1.24)
Health Outcomes Indicators^**e**^	2 (1.24)
Health Outcomes Indicators^**e**^ + Other Variables^**g**^	1 (0.62)
Not Reported	9 (5.59)

^a^Five studies established groups with different types of interventions and control measures, totaling 206.

^b^d, day; m, month, Three studies used both cost-effectiveness and cost-utility evaluation methods, with differing time horizons set for each method.

^c^CEA, cost-effectiveness analyses. CMA, cost-minimization analyses. CUA, cost-utility analyses. CBA, cost-benefit analyses.

^d^Exclusion of studies that did not conduct deterministic sensitivity analyses, resulting in 161 studies.

^e^“Health outcomes indicators variables” mainly involving health utility and clinical effective rate.

^f^“Other costs variables” mainly involving treatment fees, diagnostic test fees, medical service fees, and fees of absenteeism.

^g^“Other variables” mainly involving discounting rate, probability of disease state transition, hospitalization days, patient medication adherence and patient income.

#### 3.1.2 Outcomes

Outcomes were categorized by the number of endpoints for each study. Among included studies, 187 (94.44%) used a single endpoint, 4 (2.02%) used two endpoints, and 7 (3.54%) used three or more. Regarding effectiveness outcomes, 7 studies (3.47%) employed final end-points, including disease-attributable mortality (*n* = 2), disease event incidence or recurrence rate (*n* = 8); as for intermediate end-points, primarily comprised clinical metrics, including clinical effective rate/improvement rate/remission rate (*n* = 144) (25 studies used diagnostic criteria for TCM syndromes), biomarkers/physiological indicators (*n* = 29), scoring/index scales (*n* = 22), time to improvement of symptoms/signs (n=16), disease-specific efficacy indicators (*n* = 14), incidence of adverse drug events (*n* = 12), imaging or laboratory test indicators (*n* = 7). All CUA studies (47, 23.74%) used Quality-Adjusted Life Years (QALYs) as the health utility outcomes, and benefit outcome for CBA study includes the lost work time costs and hospitalization costs that can be saved by patients and their families through CHM treatment.

#### 3.1.3 Incremental analysis and willingness-to-pay (WTP) thresholds

181 included studies employed CEA or CUA, among which 155 (78.28%) conducted incremental analyses. Specifically, 111 studies (56.06%) reported the Incremental Cost-Effectiveness Ratio (ICER), 33 (16.67%) reported the Incremental Cost-Utility Ratio (ICUR), and 9 (4.55%) calculated both ICER and ICUR. Of the remaining studies, 19 (9.60%) calculated only average cost-effectiveness or cost-utility ratios, while 7 (3.54%) analysed costs and effects separately.

A total of 97 studies (48.99%) reported WTP thresholds, 57 (28.79) adopted per capita gross domestic product as the threshold (GDP) (43 compared with ICUR, 13 with ICER, 1 with both ICER and ICUR), 31 (15.66%) used per capita disposable income (CDI) (all compared with ICER), and 3 applied patient-reported WTP values (both comparing ICER).

In addition to standard benchmarks, several studies employed alternative sources for their thresholds. Three studies referenced previously published literature, while one utilized a retrospective review of historical medical data, establishing the minimum treatment cost as the threshold. For ICER comparisons specifically, one study derived the threshold from expert consultation, and another referenced the 2022 China Health Statistics Yearbook (29), adopting the average medical cost per fracture in public hospitals as the payment threshold. Notably, one study applied the ICUR threshold recommended by the Institute for Clinical and Economic Review in the United States (30). (One study applied distinct thresholds for both ICER and ICUR analyses, so the total count exceeds 97).

#### 3.1.4 Other details

As stipulated in the guidelines, discounting is recommended for studies with a time horizon longer than one year (31). PE should discount cost and health outcomes that occur in the future, converting future cost and health outcomes to the value at baseline. Expect for two studies, 31 studies (15.66%) with time horizon exceeding one year reported discount rates, with 28 studies at 5% which was recommended in guidelines, two studies at 3%, and one at 8%.

A total of 155 studies (78.28%) acknowledged study limitations. The most prevalent issue identified was the low quality of the included original data. Other frequently highlighted shortcomings included short study time horizons with a lack of long-term follow-up for health outcomes, incomplete cost calculations or missing data, and small sample sizes. Additionally, the lack of scientifically validated WTP thresholds were commonly noted.

### 3.2 Quality assessment of included studies

#### 3.2.1 Quality assessment of studies using the CHEERS 2022 checklist

According to the evaluation of the quality of included studies using the CHEERS 2022 checklist, the average compliance rate across 198 studies was 54.14%, with the highest rate at 76.79% and the lowest at 28.00%. Based on quality grading, 7 studies (3.54%) were rated as moderate quality, 87 studies (43.94%) as poor, 104 studies (52.53%) as extremely poor, and no study of high quality.

Based on the scoring for each item, as presented in Table 2, among the 28 items, six achieved an overall average compliance rate more than 85.00%, including Item 2 (Abstract), Item 3 (Background and Objectives), Item 11 (Selection of Outcomes), Item 14 (Costs), Item 22 (Analytic Inputs) and Item 23 (Summary of Main Results), with the highest compliance rate for a single item reaching 98.23%. Conversely, Item 4 (Economic Analyses Plan), Item 18 (Subgroup Analyses), Item 19 (Characterizing Distributional Effects), and Item 25 (Effect of Engagement with Patients and Others Affected by the Study), showed low compliance rates, with average compliance rates not exceeding 10.00%.

**Table 2:** Compliance rates for CHEERS 2022 checklist items.

**Dimensions**	**Evaluation item**	**Yes *N* (%)**	**Partially *N* (%)**	**No + N/A *N* (%)**	**Compliance rate (%) (Mean ±SD)**
Title	Item 1	99 (50.00)	98 (49.49)	1 (0.51)	74.75 ± 25.56
	Item 2	180 (90.91)	10 (5.05)	8 (4.04)	93.43 ± 22.13
Introduction	Item 3	150 (75.76)	48 (24.24)	0	87.88 ± 21.48
Method	Item 4	3 (1.52)	6 (3.03)	189 (95.45)	3.03 ± 14.81
	Item 5	62 (31.31)	55 (27.78)	81 (40.91)	45.20 ± 42.33
	Item 6	8 (4.04)	62 (31.31)	128 (64.65)	19.70 ± 28.34
	Item 7	76 (38.38)	121 (61.11)	1 (0.51)	68.94 ± 24.83
	Item 8	22 (11.11)	116 (58.59)	60 (30.30)	40.40 ± 30.79
	Item 9	41 (20.71)	140 (70.71)	17 (8.59)	56.06 ± 26.44
	Item 10^*^	19 (9.60)	12 (6.06)	2 (1.01) + 165 (83.33)	75.76 ± 30.93
	Item 11	191 (96.46)	7 (3.54)	0	98.23 ± 9.26
	Item 12	146 (73.74)	20 (10.10)	32 (16.16)	78.79 ± 37.76
	Item 13	122 (61.62)	27 (13.64)	49 (24.75)	68.43 ± 42.76
	Item 14	163 (82.32)	32 (16.16)	3 (1.52)	90.40 ± 21.58
	Item 15	61 (30.81)	39 (19.70)	98 (49.49)	40.66 ± 43.93
	Item 16^*^	63 (31.82)	44 (22.22)	1 (0.51) + 90 (45.45)	78.70 ± 25.76
	Item 17^*^	29 (14.65)	35 (17.68)	44 (22.22) + 90 (45.45)	43.06 ± 40.71
	Item 18	15 (7.58)	4 (2.02)	179 (90.40)	8.59 ± 27.17
	Item 19	2 (0.99)	0	196 (98.99)	1.01 ± 10.02
	Item 20	147 (74.24)	14 (7.07)	37 (18.69)	77.78 ± 39.49
	Item 21	9 (4.55)	3 (1.52)	186 (93.94)	5.30 ± 21.60
Result	Item 22	144 (72.73)	49 (24.75)	5 (2.53)	85.10 ± 25.54
	Item 23	175 (88.38)	23 (11.62)	0	94.19 ± 16.06
	Item 24	117 (59.09)	44 (22.22)	37 (18.69)	70.20 ± 39.30
	Item 25	3 (1.52)	1 (0.51)	194 (97.98)	1.77 ± 12.72
	Item 26	154 (77.78)	40 (20.20)	4 (2.02)	87.88 ± 23.72
Other relevant information	Item 27	20 (10.10)	81 (40.91)	97 (48.99)	30.56 ± 33.24
	Item 28	38 (19.19)	0	160 (80.81)	19.19 ± 39.48
Total (%) (Mean ± SD, Range)	54.14% ± 9.16% (28.00% ~ 76.79%)				

Specific items are detailed in Supplementary Table 2. Items marked with “^*^” have applicable conditions.

#### 3.2.2 Quality assessment of studies using the BMJ guideline

When employing the BMJ guidelines for quality assessment, the overall average compliance rate was 71.08% (ranging between 45.00% and 93.75%) for the 198 included studies. Among these, 3 studies (1.52%) were rated as high quality, 126 studies (63.64%) as moderate, 53 studies (26.77%) as poor, and 16 studies (8.08%) as extremely poor.

Scoring was conducted across 35 items, and the detailed compliance rates for each item are presented in Table 3. Twelve items achieved an overall average compliance rate exceed 85.00%. Including Item 1 (Study Questions), Item 2 (Economic Importance of Study Questions), Item 6 (Evaluation Methods), Item 11 (Health Outcomes), Item 14 (Productivity Changes), Item 14 (Models), Item 23 (Discounting Rate), Item 30 (Comparison of Interventions), Item 32 (Study Results), Item 33 (Addresses the Study Questions posed), and Item 34 (Study Conclusions). However, the included studies showed low compliance with Item 15 (The Relevance of Productivity Changes to Study Questions) and Item 19 (Impacts of Inflation), with only 13 and 4 studies complied respectively.

**Table 3:** Compliance rates for the BMJ guidelines items.

**Dimensions**	**Evaluation item**	**Yes *N* (%)**	**Partially *N* (%)**	**No + N/A *N* (%)**	**Compliance rate (%) (Mean ±SD)**
**Study design**					
Study question	Item 1	190 (95.96)	8 (4.04)	0	97.98 ± 9.87
	Item 2	171 (86.36)	16 (8.08)	11 (5.56)	90.40 ± 25.86
Comparative approach selection	Item 3	128 (64.65)	11 (5.56)	59 (29.80)	67.42 ± 45.47
	Item 4	80 (40.40)	118 (59.60)	0	70.20 ± 24.50
	Item 5	119 (60.10)	35 (17.68)	44 (22.22)	68.94 ± 41.33
Evaluation method	Item 6	195 (98.48)	2 (1.01)	1 (0.51)	98.99 ± 8.67
	Item 7	104 (52.53)	27 (13.64)	67 (33.84)	59.34 ± 45.63
**Data collection**					
Data validity	Item 8	195 (98.48)	0	3 (1.52)	98.48 ± 12.25
	Item 9^*^	70 (35.35)	28 (14.14)	12 (6.06) + 88 (44.44)	76.36 ± 34.34
	Item 10^*^	64 (32.32)	8 (4.04)	16 (8.08) + 110 (55.56)	77.27 ± 39.33
Measurement and evaluation of benefit	Item 11	194 (97.98)	4 (2.02)	0	98.99 ± 7.05
	Item 12	52 (26.26)	21 (10.61)	125 (63.13)	31.57 ± 43.64
	Item 13	71 (35.86)	47 (23.74)	81 (40.91)	47.73 ± 43.72
Cost	Item 14^*^	21 (10.61)	1 (0.51)	0 + 176 (88.89)	97.73 ± 10.66
	Item 15	13 (6.57)	7 (3.54)	178 (89.90)	8.33 ± 26.06
	Item 16	64 (32.32)	14 (7.07)	120 (60.61)	35.86 ± 46.20
	Item 17	127 (64.14)	34 (17.17)	37 (18.69)	72.73 ± 39.52
	Item 18	154 (77.78)	16 (8.08)	28 (14.14)	81.82 ± 35.95
	Item 19	4 (1.98)	13 (6.44)	185 (91.58)	5.30 ± 18.43
Model	Item 20^*^	85 (42.93)	22 (11.11)	1 (0.51) + 90 (45.45)	88.89 ± 21.97
	Item 21^*^	40 (20.20)	57 (28.79)	11 (5.56) + 90 (45.45)	63.43 ± 31.77
**Analyses and interpretation of results**					
Cost and benefit adjustment	Item 22	154 (77.78)	17 (8.59)	27 (13.64)	82.07 ± 35.54
	Item 23^*^	31 (15.66)	0	2 (1.01) + 165 (83.33)	93.94 ± 24.23
	Item 24^*^	23 (11.62)	1 (0.51)	9 (4.55) + 165 (83.33)	71.21 ± 45.12
	Item 25^*^	44 (22.22)	0	118 (59.60) + 33 (16.67)	28.48 ± 45.27
Acceptability and uncertainty	Item 26	136 (68.69)	22 (11.11)	40 (20.20)	74.24 ± 40.53
	Item 27	156 (78.79)	5 (2.53)	37 (18.69)	80.05 ± 39.26
	Item 28	81 (40.91)	68 (34.34)	49 (24.75)	58.08 ± 39.80
	Item 29	147 (74.24)	3 (1.52)	48 (24.24)	75.00 ± 42.97
	Item 30	198 (100.00)	0	0	100.00 ± 0.00
	Item 31	155 (78.28)	0	43 (21.72)	78.28 ± 41.34
Reporting of result	Item 32	192 (96.97)	5 (2.53)		98.23 ± 10.54
	Item 33	197 (99.49)	1 (0.51)	0	99.75 ± 3.55
	Item 34	198 (100.00)	0	0	100.00 ± 0.00
	Item 35	123 (62.12)	0	75 (37.88)	62.12 ± 48.63
Total (%) (Mean ± SD, Range)	71.08% ± 9.41% (45.00% ~ 93.75%)				

Specific items are detailed in Supplementary Table 2. Items marked with “^*^” have applicable conditions.

#### 3.2.3 Quality assessment of studies using the QHES scale

The overall average quality score evaluated by the QHES scale was 68.83 (range: 27.00 - 99.00), indicating significant quality variation among studies. In terms of the specific quality grading, 72 studies (36.36%) were rated as high quality, 103 studies (52.02%) as moderate quality, and 23 studies (11.62%) as poor quality.

As presented in Table 4, the average compliance rate across 16 items ranged from 9.90% to 98.02%, with a gap nearly 90.00% between the highest and lowest rates, indicating a notable disparity. Specifically, four items, i.e., Item 1, Item 7, Item 10 and Item 15, achieved compliance rates more than 85.00%. Conversely, compliance rates for Item 4 was below 10.00%.

**Table 4:** Scores and compliance rates for QHES scale items.

**Evaluation item**	**Yes *N* (%)**	**No *N* (%)**	**Average Score (Mean±SD)**
Item 1: Study Objective	181 (91.41)	17 (8.59)	6.34 ± 2.05
Item 2: Study Perspective	140 (70.71)	58 (29.29)	2.81 ± 1.83
Item 3: Estimation of Analysis Variable	80 (40.40)	118 (59.60)	3.21 ± 3.93
Item 4: Subgroup Analysis	19 (9.60)	179 (90.40)	0.10 ± 0.30
Item 5: Statistical Analysis and Sensitivity Analysis	140 (70.71)	58 (29.29)	6.42 ± 4.08
Item 6: Incremental Cost Analysis	155 (78.28)	43 (21.72)	4.66 ± 2.50
Item 7: Data Acquisition Method	190 (95.96)	8 (4.04)	4.80 ± 0.98
Item 8: Study Time Horizon and Discounting	114 (57.58)	84 (42.42)	4.05 ± 3.46
Item 9: Cost Quantity Estimation	94 (47.47)	104 (52.53)	3.84 ± 4.01
Item 10: Outcome Indicator	177 (89.39)	21 (10.61)	5.38 ± 1.84
Item 11: Measurement Methods for Health Outcome	138 (69.70)	60 (30.30)	4.92 ± 3.21
Item 12: Economic Models and Analytical Method	141 (71.21)	57 (28.79)	5.62 ± 3.66
Item 13: Statement and justification of models, assumptions, and limitations	73 (36.87)	125 (63.13)	2.56 ± 3.38
Item 14: Study Bias	155 (78.28)	43 (21.72)	4.66 ± 2.50
Item 15: Study Result	194 (97.98)	4 (2.02)	7.84 ± 1.12
Item 16: Funding Source	101 (51.01)	97 (48.99)	1.56 ± 1.50
Total (Mean ± SD, Range)	68.83 ± 14.69 (27.00 ~ 99.00)		

Specific items are detailed in Supplementary Table 2.

#### 3.2.4 Results comparisons of three quality assessment tools

**Figure 2:**
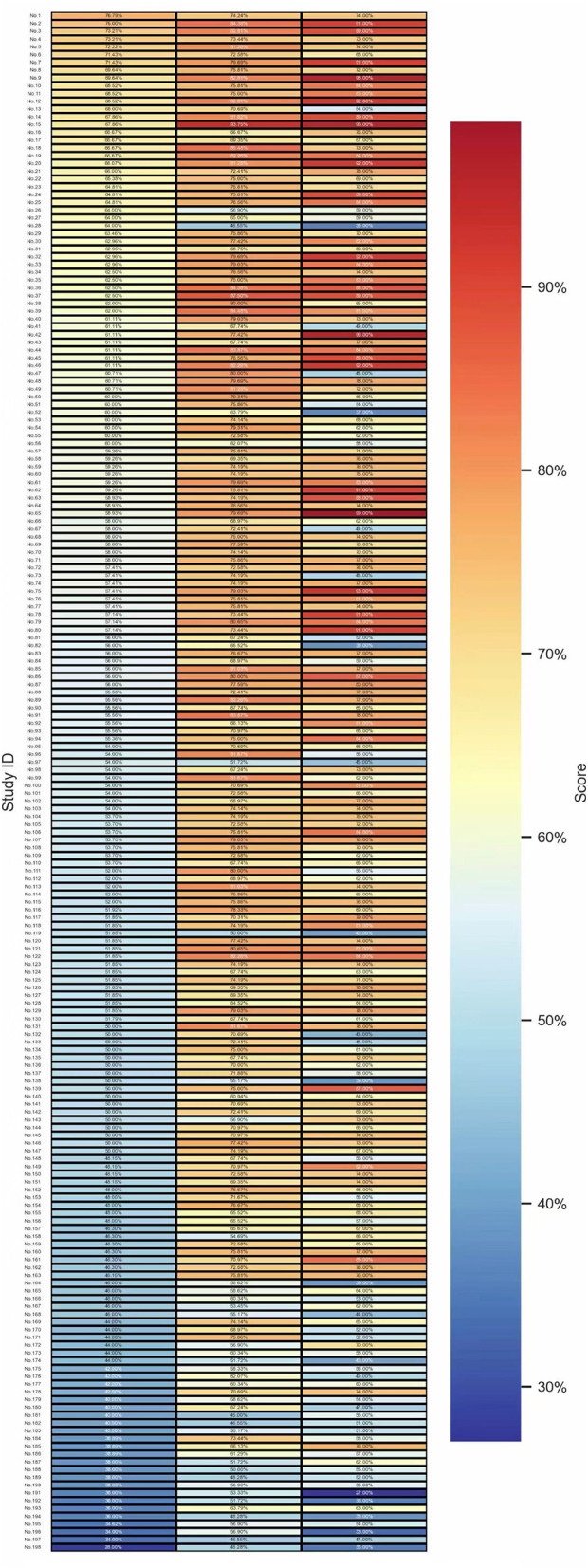
Heatmap of CHEERS 2022, BMI, QHES scores (198 studies).

A heatmap visualizing the quality assessment scores of the 198 studies across the three assessment tools is presented in Figure 2. A vertical analysis reveals that the CHEERS 2022 column is predominantly characterized by cool tones (blue), indicating lower compliance rates; conversely, the BMJ column displays warmer hues, suggesting higher overall adherence. Horizontally, no single study achieved a “high quality” rating across all three instruments. In contrast, studies such as Nos. 97, 119, 174, and 191–198 were consistently coded in blue or dark blue, corresponding to “poor” or “extremely poor” ratings across all instruments—thereby signaling significant quality deficiencies. Furthermore, a notable subset of studies exhibited marked color variation across the different tools, highlighting notable discrepancies between their reporting and methodological quality.

The ICC, first proposed by Bartko in 1966, is used to evaluate the consistency or reliability among different quantitative measurement methods (32). In this study, ICC was employed to assess the overall agreement of the three assessment tools across the 198 included studies (Table 5). The resulting ICC value was 0.580, indicating a moderate level of agreement (26), which was statistically significant (*p* < 0.05).

**Table 5:** Intraclass correlation coefficient of the assessment results of three tools.

**Intraclass correlation coefficient**	**Intraclass correlation^b^**	**95% CI**		***F*** **Test with True Value 0**		
		**Lower bound**	**Upper bound**	**df1**	**df2**	**Sig**
Single measures	0.580^a^	0.505	0.651	197	394	< 0.001
Average measures	0.806^c^	0.754	0.848	197	394	< 0.001

Two-way mixes effects model where tools effects are random and measures effects are fixed.

^a^The estimator is the same, whether the interaction effect is present or not.

^b^Type C intraclass correlation coefficients using an absolute agreement definition.

^c^This estimate is computed assuming the interaction effect is absent, because it is not estimable otherwise.

**Figure 3:**
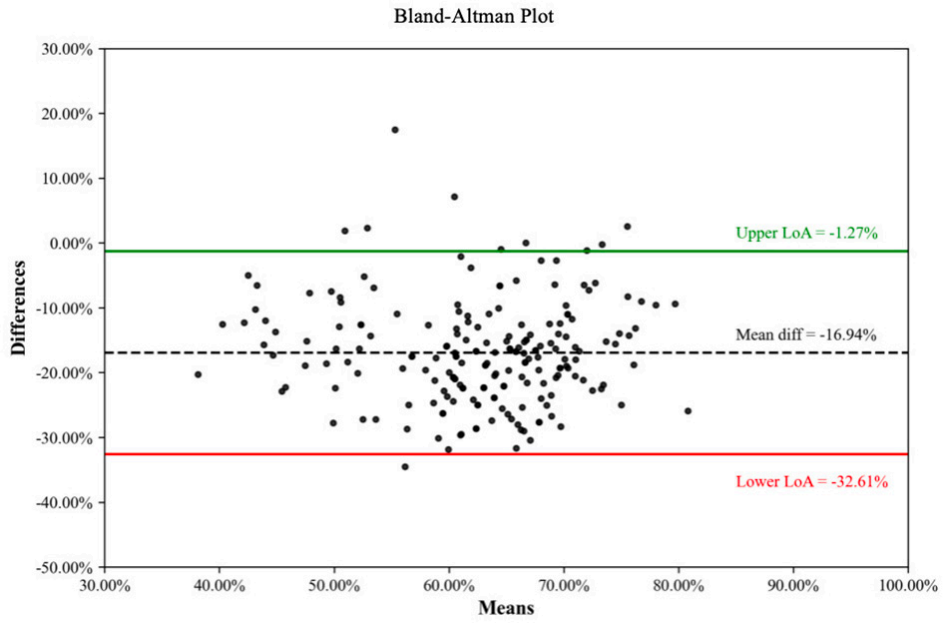
Consistency of comparison between CHEERS 2022 and BMJ.

**Figure 4:**
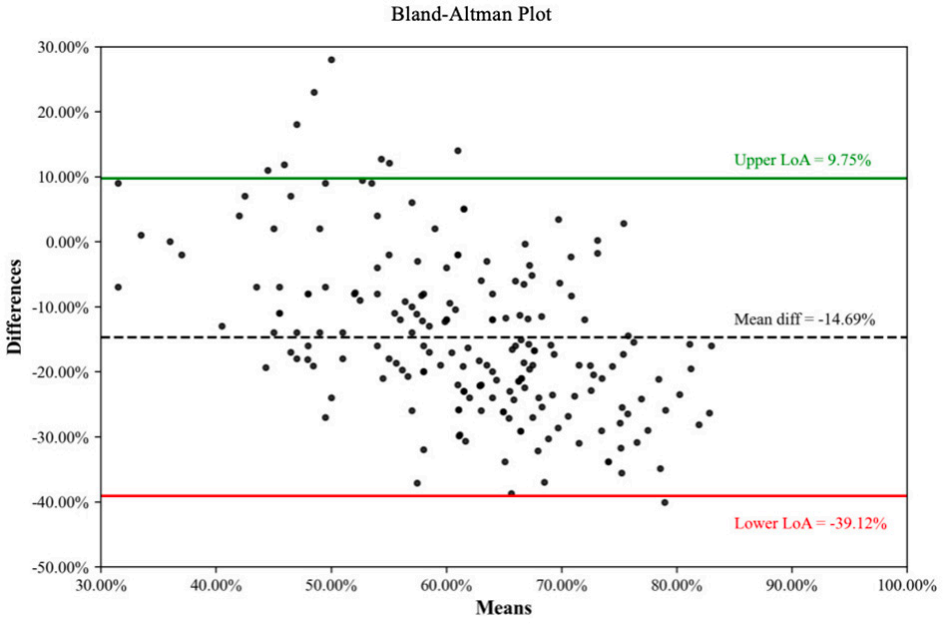
Consistency of comparison between CHEERS 2022 and QHES.

**Figure 5:**
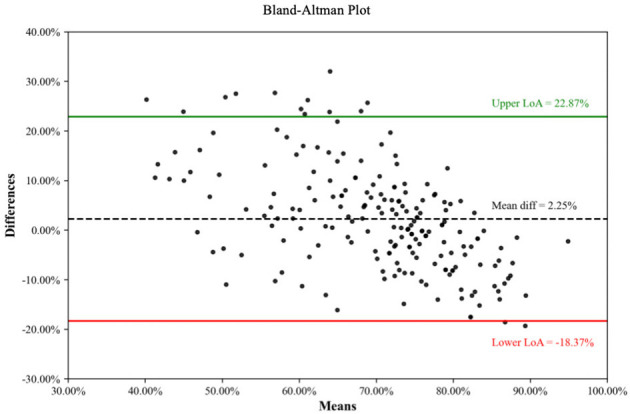
Consistency of comparison between BMJ and QHES.

And Bland-Altman analysis was performed for each pair of assessment tools (Figures 3–5) (33). When comparing CHEERS 2022 with the BMJ, a significant negative bias was observed, with a mean difference of −16.94% and 95% limits of agreement (LoA) ranging from−32.61% to −1.27%. Similarly, the comparison between CHEERS 2022 and QHES demonstrated a large systematic difference (mean difference: −14.69%), accompanied by wide LoA (−39.12% to 9.75%). Although the mean difference between the BMJ checklist and QHES was relatively small (2.25%), the 95% LoA remained wide, spanning from −18.37% to 22.87%, reflecting substantial variability in individual study assessments.

#### 3.2.5 Subgroup and sensitivity analyses

Subgroup analyses in this study are divided by languages and different databases (See Supplementary Table 2, Table S4), The result revealed a significant quality disparity, with English-language studies (*n* = 20) consistently outperforming Chinese-language studies (*n* = 178) across all three tools, particularly in CHEERS (Mean: 68.58 ± 5.79 vs. 52.77 ± 8.46) and QHES (76.50 ± 16.52 vs. 67.93 ± 14.27), while the gap was narrower for BMJ scores (74.02 ± 10.78 vs. 70.67 ± 9.24). The ICC values indicated moderate agreement within both groups (0.588 for English-language and 0.571 for Chinese-language).

Consistent with language findings, studies indexed in Chinese databases (CNKI, WF, VIP) exhibited similar and lower quality profiles, with CHEERS scores ranging from 53.11 to 54.47, BMJ scores around 70.83–71.51, and QHES scores approximately 68.50-69.45. In contrast, studies indexed in international databases (PubMed, Embase) achieved significantly higher scores across all tools, particularly in CHEERS (62.87 and 62.79, respectively) and BMJ (>74.00). The small subset of studies indexed in the Cochrane Library (*n* = 7) showed intermediate performance (CHEERS: 56.40; BMJ: 71.42; QHES: 68.86). The ICC values indicated moderate agreement across all databases, ranging from 0.423 to 0.556.

And sensitivity analyses confirmed the robustness of the primary results (see Supplementary Table 2, Table S4). After excluding 7 studies with undefined CHM interventions, the re-calculated quality scores showed no statistically significant differences compared to the original dataset (*P* = 0.87 for CHEERS, *P* = 0.92 for BMJ, and *P* = 0.65 for QHES). Similarly, the systematic biases in the Bland-Altman analyses remained stable (e.g., CHEERS vs. BMJ mean difference shifted marginally from −16.94 to −16.89).

## 4 Discussion

Our review found that the number of PE studies on CHM in the past six years has shown an increasing trend compared to previous years (12, 18). This is likely attributable to the reimbursement reforms in China, which established economic evaluation as a pivotal determinant for the inclusion of drugs and health technologies (34). However, given that the vast majority of these studies originated from mainland China, the current volume of evidence remains limited relative to clinical needs. Specifically, while 48 types of CHM drugs were added to the NRDL during this period (27, 35), only five were supported by published economic evaluations. Similarly, among the 105 newly approved CHM products, only eight were accompanied by publicly available PE studies.

Besides, our findings indicate a tangible improvement in reporting and methodological quality compared to earlier CHM PE studies, evidenced by higher average scores relative to previous benchmarks [CHEERS 2022: 43.00% (18); BMJ: 62.00% (11); QHES: 63.37 (6)]. This progress may be attributed to the release of the *China Guidelines for Pharmacoeconomic Evaluations* in 2020 (36). Notably, quality assessment results varied by instruments: while adherence to BMJ guidelines yielded relatively high scores, compliance with the CHEERS 2022 checklist was significantly lower, and the QHES results suggested only moderate methodological rigor. This discrepancy implies that while many studies may be fundamentally sound in their overall design, they frequently fail to meet contemporary standards for transparency and granular reporting.

Despite adequate reporting of basic study contexts, transparency regarding health economic analysis plans (HEAPs) and granular cost data remains a critical deficiency. Both CHEERS and BMJ confirmed that the majority of studies successfully defined their objectives and primary outcomes. However, a critical gap identified by CHEERS 2022 was the absence of pre-specified protocols, with only a small fraction of studies indicating that a HEAP was developed and accessible, which may lead to selective reporting or selective selection of results after data analyses, thereby introducing bias into the economic evaluation results and seriously compromising the reproducibility of the research (37). Furthermore, cost reporting was consistently opaque. According to BMJ guidelines, only one-third of studies reported resource quantities separately from unit costs. Similarly, the CHEERS evaluation found that only 40.66% of studies adequately reported reference years, while details regarding currency conversion and inflation adjustments were rarely provided. This lack of granularity hinders the transferability of results to other settings and complicates cross-study comparisons.

Methodological designs frequently lack theoretical robustness, particularly concerning structural validity, subgroup analysis, and threshold selection. Although 95.96% of studies explicitly stated their data abstraction methodology and most provided clear objectives, significant limitations persist. Evaluations focused predominantly on “average” costs and effectiveness, neglecting in-depth explorations of health equity or heterogeneous subgroups. The failure to assess variations across different populations, such as disease stages, TCM syndrome patterns, or socioeconomic groups—constrains the precise identification of target patient groups and weakens the evidence base for equitable resource allocation (38). Additionally, nearly two-thirds of studies failed to sufficiently justify their choice of economic model, raising concerns about structural validity. Furthermore, the scientific basis for WTP thresholds remains weak, while guidelines recommends using 1–3 times per capita GDP per QALY for CUA (31, 39, 40), no universally accepted standard exists for CEA based on clinical outcomes. Our review revealed that 14 CEA studies (7.07%) inappropriately adopted GDP as the thresholds, while 31 studies utilized per CDI without justification. Consequently, the reliability of evolution results based on these unverified thresholds warrants further scrutiny.

We also found that a fundamental disconnect persists between outcome metrics and the clinical characteristics of CHM. Health outcomes often failed to capture the holistic nature of CHM. The majority of included studies relied on conventional clinical efficacy indicators, while only 25 studies incorporated TCM syndrome scores or efficacy measures. Moreover, the utility instruments employed were rarely grounded in TCM theory. While international scales (e.g., EQ-5D, SF-6D) facilitate comparison, they may not fully capture the multidimensional effects of CHM (41–44), whereas existing CHM-specific utilities scales are often limited by specialized terminology and item complexity (45). This misalignment indicates that current PE frameworks have yet to fully adapt to the specific theoretical paradigms of CHM.

Furthermore, the quality of evidence is significantly stratified by publication venue, with international journals demonstrating superior adherence to quality standards compared to domestic Chinese publications. Subgroup analyses of this review revealed that studies published in English journals or indexed in international databases consistently outperformed those in Chinese journals or databases across all three tools. This disparity suggests that international journals likely enforce stricter reporting or methodological guidelines and peer-review processes (6). In contrast, domestic Chinese studies often demonstrate insufficient adherence to standard PE submission requirements, highlighting an urgent need for domestic journals to align with international standards to bridge this quality gap.

Finally, the results demonstrated only moderate overall correlation among the three quality assessment instruments (26), with Bland-Altman analyses revealing systematic scoring biases and wide confidence intervals across all pairwise comparisons. These findings highlight a distinct discrepancy between reporting quality and methodological quality, indicating that rigorous methodological design does not necessarily guarantee clear and complete reporting. Consequently, the choice of instrument systematically influences quality scores and final grading, suggesting that quality assessments should not be confined to a single evaluative dimension.

### 4.1 Limitations and implications for future studies

This systematic review has several limitations. First, the literature search was restricted to six major Chinese and English databases, and only publications in these two languages were included. Consequently, relevant studies published in non-Chinese or non-English databases may have been excluded, introducing a risk of language and publication bias. Second, our search strategy involved several eligibility criteria, including that studies must be published in peer-reviewed academic journals, and as such we did not included any gray literature. Since unpublished studies often lack rigor, their exclusion may have led to an overestimation of the overall quality of studies in this field. Furthermore, given the large number of included studies and the heterogeneity in reporting, we did not perform a granular statistical analyses of specific disease names. Instead, we categorized data based on ICD-11 disease classifications. Future research is warranted to investigate disease-specific variations in the application of PE studies in greater detail. Fourth, the application of three quality assessment tools inevitably involves a degree of subjectivity. To mitigate this potential bias, the quality assessment process was conducted independently by two reviewers, with a third reviewer consulted to resolve discrepancies. Finally, the quality grading thresholds (e.g., distinguishing between “high” and “low” quality) used in this review were based on relative performance observed in previous studies rather than empirically validated absolute standards. Therefore, our quality classifications should be interpreted as relative quality rather than definitive judgments.

As critical evidence for health decision-making, our review proposes several suggestions for future studies: (i) To address the prevalent lack of transparency in study design and reporting, strict adherence to report checklists (e.g., CHEERS) is imperative. And a pre-registration of protocols are needed to minimize selective reporting bias. Furthermore, Chinese journals should align with international standards by requiring the submission of quality checklists as a prerequisite for peer review, thereby bridging the quality gap between domestic and international publications. (ii) We recommend that future studies conduct rigorous subgroup analyses on key model parameters, including transition probabilities, costs, and utility values—stratified by critical variables such as TCM syndromes, disease staging, age, and comorbidity burden. This approach will reveal the heterogeneity of treatment effects, identify specific patient subgroups that benefit most from CHM interventions, and facilitate health equity analyses to support precise resource allocation. (iii) The development of health outcomes and user-friendly health assessment scales based on TCM syndromes is essential. Advancing these modern evaluation methods will objectively quantify the multidimensional health outcomes of CHM, enhance cross-study comparability, and establish scientifically sound standards for assessing its clinical and economic value (45). While scholars have developed utility scales based on TCM theories that demonstrate good reliability and validity for the Chinese population, future research should focus on adapting these tools for broader regions and diverse populations to enhance their universality (32). (iv) Quality evaluations should avoid relying on single tool, instead, a complementary strategy integrating multiple scales is recommended for a holistic judgment. Moreover, we advocate for the development of CHM-specific extensions to universal guidelines and scales (e.g., CHEERS or QHES), so as to harmonize standard economic reporting and methodological requirements with CHM-specific elements (11, 46).

## 5 Conclusion

Quality assessments indicate that research quality has improved compared to earlier literature, yet several issues persist. These include low data transparency, inadequate methodological justification, limited analytical dimensions, and health outcome measures that fail to fully reflect the distinctive characteristics of CHM. The consistency for the assessment results of three tools was moderate and Bland-Altman analysis further revealed significant and systematic inconsistent scoring biases between tools. Future study should strictly adhere to guidelines and pre-specified research analyses plans, develop health outcome measures reflecting the characteristics of CHM, and employ a complementary multi-tool assessment strategy during quality assessment. Furthermore, existing guidelines are encouraged to expand to include assessment criteria specific to CHM to address the inconsistencies present in current quality assessment methods. So as to provide higher-quality evidence for decision-making.

## Data Availability

The raw data supporting the conclusions of this article will be made available by the authors, without undue reservation.
