# Supplementary material for: A qualitative systematic review and quality assessment of pharmacoeconomic evaluations on Chinese Herbal Medicine from 2020 to 2025
Source: Front Public Health. 2026 Feb 17;14:1738097. doi: 10.3389/fpubh.2026.1738097 (PMC12953556; doi:10.3389/fpubh.2026.1738097)
Supplement: Supplementary file 1 [file Table_1.docx]

Supplementary Table 1

## **SEARCHING STRATEGY**

1. **CNKI**

SU= ('药物经济' + '经济性评价' + '成本效果' + '成本效益' + '成本效用' + '最小成本' + 'Markov' + '决策树' + '分区生存') AND (SU=('中药' + '中成药' + '中草药' + '植物药' + '中药材' + '中药饮片' + '中药配方颗粒' + '天然药物') OR TI= ('颗粒' + '丸' + '散' + '汤' + '口服液' + '胶囊' + '注射液' + '片'))

1. **WANFANG**

题名或关键词:("药物经济" OR "经济性评价" OR "成本效果" OR "成本效益" OR "成本效用" OR "最小成本" OR "Markov" OR "决策树" OR "分区生存"))AND( 题名或关键词:("中药" OR "中成药" OR "中草药" OR "植物药" OR "中药材" OR "中药饮片" OR "中药配方颗粒" OR "天然药物") OR 标题:("颗粒" OR "丸" OR "散" OR "汤" OR "口服液" OR "胶囊" OR "注射液" OR "片")

1. **VIP**

T=('药物经济' or '经济性评价' or '成本效果' or '成本效益' or '成本效用' or '最小成本' or 'Markov' or '决策树' or '分区生存') and ((M=('中药' or '中成药' or '中草药' or '植物药' or '中药材' or '中药饮片' or '中药配方颗粒' or '天然药物') and (T=('颗粒' or '丸' or '散' or '汤' or '口服液' or '胶囊' or '注射液' or '片'))

1. **PUBMED**

| No | Query Results | Results |
| --- | --- | --- |
| #1 | "Medicine, Chinese Traditional"[Mesh] | 27,595 |
| #2 | ((Traditional Medicine, Chinese[Title/Abstract]) OR (Chinese Traditional Medicine[Title/Abstract]) OR (Traditional Chinese Medicine[Title/Abstract]) OR (Chinese Medicine, Traditional[Title/Abstract])) | 45,364 |
| #3 | #1 OR #2 | 59,770 |
| #4 | "Drugs, Chinese Herbal"[Mesh] | 61,314 |
| #5 | ((Chinese Drugs, Plant[Title/Abstract]) OR (Chinese Herbal Drugs[Title/Abstract]) OR (Herbal Drugs, Chinese[Title/Abstract]) OR (Plant Extracts, Chinese[Title/Abstract]) OR (Chinese Plant Extracts[Title/Abstract]) OR (Extracts, Chinese Plant[Title/Abstract])) | 7,197 |
| #6 | #4 OR #5 | 65,624 |
| #7 | ((Chinese patent medicine[Title/Abstract]) OR (natural medicine[Title/Abstract]) OR (Chinese Medicinal Materials[Title/Abstract]) OR (Botanical medicine[Title/Abstract]) OR (Chinese herbal pieces[Title/Abstract]) OR (Chinese medicinal granula[Title/Abstract]) OR (traditional chinese medicine decoction[Title/Abstract])) | 2,809 |
| #8 | "granule*"[Title/Abstract] OR "decoction*"[Title/Abstract] OR "oral liquid*"[Title/Abstract] OR "pill*"[Title/Abstract] | 127,437 |
| #9 | #3 OR #6 OR #7 OR #8 | 227,509 |
| #10 | "Economics, Pharmaceutical"[Mesh] | 3,174 |
| #11 | ((Pharmacoeconomics[Title/Abstract]) OR (Pharmaceutical Economics[Title/Abstract]) OR (Pharmacy Economics[Title/Abstract]) OR (Economic, Pharmacy[Title/Abstract]) OR (Economics, Pharmacy[Title/Abstract]) OR (Pharmacy Economic[Title/Abstract])) | 2,272 |
| #12 | #10 OR #11 | 4,747 |
| #13 | "Cost-Effectiveness Analysis"[Mesh] | 1,673 |
| #14 | ((Analysis, Cost-Effectiveness[Title/Abstract]) OR (Cost Effectiveness Analysis[Title/Abstract]) OR (Cost Effectiveness[Title/Abstract]) OR (Effectiveness, Cost[Title/Abstract]) OR (Cost Effectiveness Ratio[Title/Abstract]) OR (Cost Effectiveness Ratios[Title/Abstract]) OR (Effectiveness Ratio, Cost[Title/Abstract]) OR (Ratio, Cost Effectiveness[Title/Abstract])) | 93,819 |
| #15 | #13 OR #14 | 93,882 |
| #16 | "Cost-Benefit Analysis"[Mesh] | 99,730 |
| #17 | ((Analysis, Cost-Benefit[Title/Abstract]) OR (Cost-Benefit Analyses[Title/Abstract]) OR (Cost Benefit Analysis[Title/Abstract]) OR (Analyses, Cost Benefit[Title/Abstract]) OR (Analysis, Cost Benefit[Title/Abstract]) OR (Cost Benefit Analyses[Title/Abstract]) OR (Cost Benefit[Title/Abstract]) OR (Cost Benefit[Title/Abstract]) OR (Benefit and Cost[Title/Abstract]) OR (Benefits and Costs[Title/Abstract]) OR (Costs AND Benefits[Title/Abstract]) OR (Cost-Benefit Data[Title/Abstract]) OR (Cost Benefit Data[Title/Abstract]) OR (Data, Cost-Benefit[Title/Abstract]) OR (Cost-Utility Analysis[Title/Abstract]) OR (Analysis, Cost-Utility[Title/Abstract]) OR (Cost-Utility Analyses[Title/Abstract]) OR (Cost Utility Analysis[Title/Abstract]) OR (Economic Evaluation[Title/Abstract]) OR (Economic Evaluations[Title/Abstract]) OR (Evaluation, Economic[Title/Abstract])） | 79,776 |
| #18 | #16 OR #17 | 151,510 |
| #19 | [((Cost Minimization Analysis[Title/Abstract]) OR (Cost-Minimization Analysis[Title/Abstract]) OR (analysis, Cost-Minimization[Title/Abstract]) OR (Analysis, Cost-Minimization[Title/Abstract]) OR (Cost-Minimization Analyses[Title/Abstract]) OR (Cost Minimization Analysis[Title/Abstract]))](https://pubmed.ncbi.nlm.nih.gov/?term=((Cost+Minimization+Analysis[Title/Abstract])+OR+(Cost-Minimization+Analysis[Title/Abstract])+OR+(analysis,+Cost-Minimization[Title/Abstract])+OR+(Analysis,+Cost-Minimization[Title/Abstract])+OR+(Cost-Minimization+Analyses[Title/Abstract])+OR+(Cost+Minimization+Analysis[Title/Abstract]))&sort=&page=1) | 805 |
| #20 | "Decision Trees"[Mesh] | 13,773 |
| #21 | ((Decision Tree[Title/Abstract]) OR (Tree, Decision[Title/Abstract]) OR (Trees, Decision[Title/Abstract]) OR (Markov[Title/Abstract]) OR (Partitioned Survival Model[Title/Abstract])) | 52,267 |
| #22 | #20 OR #21 | 61,776 |
| #23 | #12 OR #15 OR #18 OR #18 OR #22 | 374,985 |
| #24 | #8 AND #23 from 2020 - 2025 | 337 |

1. **Cochrane Library**

| No. | Query Results | Results |
| --- | --- | --- |
| #1 | MeSH descriptor: [Medicine, Chinese Traditional] explode all trees | 1,877 |
| #2 | (Traditional Medicine, Chinese; Chinese Traditional Medicine; Traditional Chinese Medicine; Chung I Hsueh; Chinese Medicine, Traditional; Zhong Yi Xue; Hsueh, Chung I; Tongue Diagnoses, Traditional; Tongue Diagnosis, Traditional; Traditional Tongue Assessments; Tongue Assessment, Traditional; Traditional Tongue Assessment; Traditional Tongue Diagnosis; Traditional Tongue Diagnoses):ti,ab,kw (Word variations have been searched) | 0 |
| #3 | #1 or #2 | 1,877 |
| #4 | MeSH descriptor: [Drugs, Chinese Herbal] explode all trees | 4,806 |
| #5 | (Extracts, Chinese Plant; Chinese Plant Extracts; Plant Extracts, Chinese; Chinese Herbal Drugs; Herbal Drugs, Chinese; Chinese Drugs, Plant):ti,ab,kw (Word variations have been searched) | 267 |
| #6 | (Chinese patent medicine):ti,ab,kw | 290 |
| #7 | (natural medicine):ti,ab,kw | 1,259 |
| #8 | (Chinese Medicinal Materials):ti,ab,kw | 19 |
| #9 | (Botanical medicine):ti,ab,kw | 66 |
| #10 | (Chinese medicinal granula):ti,ab,kw | 1 |
| #11 | #4 or #5 | 4,855 |
| #12 | (chinese medicine decoction):ti,ab,kw | 2,268 |
| #13 | (granule or decoction or oral liquid or pill):ti | 6,978 |
| #14 | #3 or #6 or #7 or #8 or #9 or #10 or #11or #12 or #13 | 15,215 |
| #15 | MeSH descriptor: [Economics, Pharmaceutical] explode all trees | 142 |
| #16 | (Economics, Pharmacy; Economic, Pharmacy; Pharmacy Economics; Pharmacy Economic; Pharmaceutical Economics; Pharmacoeconomics):ti,ab,kw (Word variations have been searched) | 5 |
| #17 | #15 or #16 | 144 |
| #18 | MeSH descriptor: [Cost-Effectiveness Analysis] explode all trees | 268 |
| #19 | (Cost Effectiveness; Effectiveness, Cost; Effectiveness Ratio, Cost; Cost Effectiveness Ratios; Ratio, Cost Effectiveness; Cost Effectiveness Ratio; Cost Effectiveness Analysis; Analysis, Cost-Effectiveness):ti,ab,kw (Word variations have been searched) | 7,529 |
| #20 | #18 or #19 | 7,623 |
| #21 | MeSH descriptor: [Cost-Benefit Analysis] explode all trees | 11,805 |
| #22 | (Marginal Analysis; Analysis, Marginal; Marginal Analyses; Cost Benefit; Benefit and Cost; Costs and Benefits; Cost and Benefit; Benefits and Costs; Analysis, Cost Benefit; Analyses, Cost Benefit; Cost-Benefit Analyses; Cost Benefit Analysis; Analysis, Cost-Benefit; Cost Benefit Analyses; Economic Evaluation; Evaluation, Economic; Economic Evaluations; Cost Benefit Data; Data, Cost-Benefit; Cost-Benefit Data; Cost-Utility Analyses; Cost-Utility Analysis; Analysis, Cost-Utility; Cost Utility Analysis):ti,ab,kw (Word variations have been searched) 9 | 9 |
| #23 | #21or #22 | 11,805 |
| #24 | MeSH descriptor: [Costs and Cost Analysis] explode all trees | 16,890 |
| #25 | (Cost-Minimization Analysis; Analysis, Cost-Minimization; Cost Minimization Analysis; Analyses, Cost-Minimization; Cost-Minimization Analyses; Pricing; Comparisons, Cost; Cost Comparisons; Comparison, Cost; Cost Comparison; Cost Measures; Cost Measure; Measure, Cost; Measures, Cost; Costs; Cost; Analyses, Cost; Cost Analyses; Cost Analysis; Analysis, Cost; Costs and Cost Analyses; Cost, Cost Analysis; Costs, Cost Analysis; Affordability; Affordabilities):ti,ab,kw (Word variations have been searched) | 0 |
| #26 | #24 or #25 | 16,890 |
| #27 | MeSH descriptor: [Markov Chains] explode all trees | 659 |
| #28 | (Chain, Markov; Markov Process; Markov Processes; Processes, Markov; Markov Chain; Process, Markov; Chains, Markov):ti,ab,kw | 0 |
| #29 | #27 or #28 | 659 |
| #30 | MeSH descriptor: [Decision Trees] explode all trees | 338 |
| #31 | (Decision Tree; Tree, Decision; Trees, Decision):ti,ab,kw | 266 |
| #32 | #30 or #31 | 434 |
| #33 | (Partitioned Survival Model):ti,ab,kw | 333 |
| #34 | MeSH descriptor: [Cost-Benefit Analysis] explode all trees | 11,805 |
| #35 | (Marginal Analysis; Analysis, Marginal; Marginal Analyses; Cost Benefit; Benefit and Cost; Costs and Benefits; Cost and Benefit; Benefits and Costs; Analysis, Cost Benefit; Analyses, Cost Benefit; Cost-Benefit Analyses; Cost Benefit Analysis; Analysis, Cost-Benefit; Cost Benefit Analyses; Economic Evaluation; Evaluation, Economic; Economic Evaluations; Cost Benefit Data; Data, Cost-Benefit; Cost-Benefit Data; Cost-Utility Analyses; Cost-Utility Analysis; Analysis, Cost-Utility; Cost Utility Analysis):ti,ab,kw | 0 |
| #36 | #34 or #35 | 11,805 |
| #37 | #17 or #20 or #23 or #26 or #29 or #32 or #33 or #36 | 21,423 |
| #38 | #14 and #37 | 69 |

1. **EMBASE**

| No. | Query Results | Results |
| --- | --- | --- |
| #1 | ('chinese medicine'/exp OR 'chinese medicine') AND [embase]/lim | 277,742 |
| #2 | ('chinese herbal medicine':ti,ab,kw OR 'chinese traditional medicine':ti,ab,kw OR 'medicine, chinese traditional':ti,ab,kw OR 'traditional chinese medicine':ti,ab,kw OR 'chinese medicine':ti,ab,kw) AND [embase]/lim | 66,635 |
| #3 | #1 OR #2 | 279,846 |
| #4 | ('chinese patent medicine'/exp OR 'chinese patent medicine') AND [embase]/lim | 1,499 |
| #5 | ('herbal medicine'/exp OR 'herbal medicine') AND [embase]/lim | 53,147 |
| #6 | ('chinese herbal medicine':ti,ab,kw OR 'chinese traditional medicine':ti,ab,kw OR 'medicine, chinese traditional':ti,ab,kw OR 'traditional chinese medicine':ti,ab,kw OR 'chinese medicine':ti,ab,kw) AND [embase]/lim | 66,635 |
| #7 | #5 OR #6 | 109,582 |
| #8 | ('natural medicine'/exp OR 'natural medicine') AND [embase]/lim | 8,379 |
| #9 | 'chinese medicine granules':ti,ab,kw AND [embase]/lim | 25 |
| #10 | ('decoction'/exp OR 'decoction') AND [embase]/lim | 16,192 |
| #11 | ('granule*':ti OR 'decoction*':ti OR 'oral liquid*':ti OR 'pill*':ti | 57,076 |
| #12 | #3 OR #4 OR #7 OR #8 OR #9 OR #10 OR #11 | [361,688](http://www--embase--com--https.embase.shd1rmyy.lwnote.com:50001/) |
| #13 | ('pharmacoeconomics'/exp OR 'pharmacoeconomics') AND [embase]/lim | 239.025 |
| #14 | ('economics, pharmaceutical':ti,ab,kw OR 'pharmaceutical economics':ti,ab,kw OR 'pharmaco-economic analysis':ti,ab,kw OR 'pharmaco-economic evaluation':ti,ab,kw OR 'pharmaco-economics':ti,ab,kw OR 'pharmacoeconomic analysis':ti,ab,kw OR 'pharmacoeconomic evaluation':ti,ab,kw OR 'pharmacoeconomics':ti,ab,kw) AND [embase]/lim | 6,991 |
| #15 | #13 OR #14 | 239,261 |
| #16 | ('cost effectiveness analysis'/exp OR 'cost effectiveness analysis') AND [embase]/lim | 211,033 |
| #17 | ('cost effectiveness':ti,ab,kw OR 'cost effectiveness ratio':ti,ab,kw OR 'cost efficiency analysis':ti,ab,kw OR 'cost-effectiveness analysis':ti,ab,kw OR 'cost effectiveness analysis':ti,ab,kw) AND [embase]/lim | 114,325 |
| #18 | #16 OR #17 | 228,758 |
| #19 | ('cost utility analysis'/exp OR 'cost utility analysis') AND [embase]/lim | 14,891 |
| #20 | ('cost utility':ti,ab,kw OR 'cost utility analysis':ti,ab,kw) AND [embase]/lim | 10,689 |
| #21 | #19 OR #20 | 15,936 |
| #22 | ('cost benefit analysis'/exp OR 'cost benefit analysis') AND [embase]/lim | 74,747 |
| #23 | ('cost analysis':ti,ab,kw OR 'cost benefit':ti,ab,kw OR 'cost benefit assessment':ti,ab,kw OR 'cost benefit evaluation':ti,ab,kw OR 'cost benefit ratio':ti,ab,kw OR 'cost-benefit analysis':ti,ab,kw OR 'cost benefit analysis':ti,ab,kw) AND [embase]/lim | 32,450 |
| #24 | #22 OR #23 | 83,184 |
| #25 | ('cost minimization analysis'/exp OR 'cost minimization analysis') AND [embase]/lim | 4,416 |
| #26 | ('cost minimization':ti,ab,kw OR 'cost minimization analysis':ti,ab,kw) AND [embase]/lim | 2,245 |
| #27 | #25 OR #26 | 4,667 |
| #28 | ('markov chain'/exp OR 'markov chain') AND [embase]/lim | 21,728 |
| #29 | ('markov chains':ti,ab,kw OR 'markov model':ti,ab,kw OR 'markov process':ti,ab,kw OR 'markov chain':ti,ab,kw) AND [embase]/lim | 23,708 |
| #30 | #28 OR #29 | 31,877 |
| #31 | ('decision tree'/exp OR 'decision tree') AND [embase]/lim | 30,325 |
| #32 | ('decision trees':ti,ab,kw OR 'decision tree':ti,ab,kw) AND [embase]/lim | 22,961 |
| #33 | #31 OR #32 | 31,125 |
| #34 | ('partitioned survival model'/exp OR 'partitioned survival model') AND [embase]/lim | 1,160 |
| #35 | #14 OR #17 OR #20 OR #23 OR #26 OR #29 OR #32 OR #33 | 527,381 |
| #36 | #12 AND #35 AND [embase]/lim AND [01-01-2020]/sd NOT [20-12-2025]/sd | 2,543 |
