# Supplementary material for: A qualitative systematic review and quality assessment of pharmacoeconomic evaluations on Chinese Herbal Medicine from 2020 to 2025
Source: Front Public Health. 2026 Feb 17;14:1738097. doi: 10.3389/fpubh.2026.1738097 (PMC12953556; doi:10.3389/fpubh.2026.1738097)
Supplement: Supplementary file 2 [file Table_2.docx]

Supplementary Table 2

**Table S1. Studies included in the review and main study characteristics**

| **Study ID** | **Country/**  **Region** | **Author Affiliation** | **TCM Drug Name** | **Disease** | **Types of Study Design** | **Intervention and Comparator** | **Evaluation Methods** |
| --- | --- | --- | --- | --- | --- | --- | --- |
| No.1: LongChen, Xu, 2023 | China | School of Management, Beijing University of Chinese Medicine | Anluo Huaxian Pill | Chronic Hepatitis B | Model-Based Study | A: Anluo Huaxian Pill plus Entecavir  B: Entecavir | CEA+CUA |
| No.2: Cong Wang, 2025 | China | Dept.of Pharmacy, Union Hospital, Tongji Medical College, Huazhong University of Science  and Technology | Xianling Gubao Capsule, Jintiange Capsule | Postmenopausal Oseoporosis | Model-Based Study | A: Xianling Gubao Capsule plus Conventional Standard Treatment  B: Jintiange Capsule plus Conventional Standard Treatment  C: No-treatment Control | CUA |
| No.3: Yumei He, 2023 | China | International Research Center for Medicinal Administration, Peking University | Bailing Capsule | Diabetic Nephropathy | Model-Based Study | A: Bailing Capsule plus Western Medicine  B: Western Medicine | CEA+CUA |
| No.4: Lu Wang, 2025 | China | Dept.of Pharmacy, Yantai Yuhuangding Hospital | Qili Qiangxin Capsule | Chronic Heart Failure | Model-Based Study | A: Qili Qiangxin Capsule plus Conventional Standard Treatment  B: Conventional Standard Treatment | CUA |
| No.5: Liang Lu, MM, 2022 | China | School of International Pharmaceutical Business, China Pharmaceutical University | Safflor Yellow Injection, (SYI) | Angina Pectoris | Model-Based Study | A: SYI  B: Conventional Standard Treatment | CEA |
| No.6: Yang shuo, 2024 | China | Institute of Basic Research in Clinical Medicine, China Academy of Chinese Medical Sciences | Zhichuanling Oral Liquid | Bronchial Asthma | Retrospective Study | A: Zhichuanling Oral Liquid plus Conventional Standard Treatment  B: Conventional Standard Treatment | CEA |
| No.7: Guoqiang Liu, 2024 | China | Dept.of Clinical Pharmacy, The Third Hospital of Hebei Medical University | Tongxinluo  Capsule, (TXL) | Acute Myocardial Infarction | Model-Based Study | A: TXL plus Conventional Standard Treatment  B: Placebo plus Conventional Standard Treatment | CEA+CUA |
| No.8: YANG Xuanru, 2024 | China | Dongzhimen Hospital, Beijing University of Chinese Medicine | Danbai Granule | Sequelae of Pelvic Inflammatory Disease | Model-Based Study | A: Danbai Granule  B: Standard Antibiotic Therapy (Levofloxacin, Metronidazole) | CEA |
| No.9: Junjie Zhu, 2024 | China | Dept.of Pharmacoeconomics, School of International Pharmaceutical Business, China Pharmaceutical University | Kang Ai Injection, Shenqi Fuzheng Injection | Non-small Cell Lung Cancer (NSCLC) | Model-Based Study | A: Kang Ai Injection plus Chemotherapy  B: Shenqi Fuzheng Injection plus Chemotherapy | CUA |
| No.10: ZHAO Xiaoxiao, 2023 | China | Institute of Basic Research in Clinical Medicine, China Academy of Chinese Medical Scicences | Longshengzhi Capsule | Cerebral Infarction | Model-Based Study | A: Longshengzhi Capsule plus Conventional Standard Treatment  B: Conventional Standard Treatment | CEA |
| No.11: Jie Pan, 2024 | China | School of Traditional Chinese Medicine, Beijing University of Chinese Medicine | Shexiang Baoxin Pill (MUSKARDIA) | Stable Coronary Artery Disease | Model-Based Study | A: Shexiang Baoxin Pill  B: Placebo | CUA |
| No.12: Yake Lou, et al. 2025 | China | Interventional Center of Valvular Heart Disease, Beijing Anzhen Hospital, Capital Medical University | Qili qiangxin Capsule | Heart Failure | Model-Based Study | A: Qili qiangxin Capsule plus Conventional Standard Treatment  B: Conventional Standard Treatment plus Placebo | CUA |
| No.13: WU Xue, 2024 | China | Institute of Basic Research in Clinical Medicine, China Academy of Chinese Medical Scicences | Shaoma Zhijing Granule, Jiuwei Xifeng Granule, Changma Xifeng Tablet | Tic Disorders In Children | Retrospective Study | A: Shaoma Zhijing Granule  B: Jiuwei Xifeng Granule  C: Shaoma Zhijing Granule plus Conventional Standard Treatment  D: Jiuwei Xifeng Granule plus Conventional Standard Treatment  E: Changma Xifeng Tablets plus Conventional Standard Treatment | CEA |
| No.14: Yang Li, 2021 | China | School of International Pharmaceutical Business, China Pharmaceutical University | Safflower Yellow Pigment, Sanqi Panax (Notoginseng) | Acute Cerebral Infarction | Model-Based Study | A: Safflower Yellow Pigment  B: Sanqi Panax (Notoginseng) | CEA |
| No.15: Zhou Wenxin, 2023 | China | West China School of Pharmacy, Sichuan University | Rehmanniaglutinosa Leaf Total Glycosides Capsule | Chronic Glomerulonephritis | Model-Based Study | A: Rehmanniaglutinosa Leaf Total Glycosides Capsule plus Irbesartan Tablet  B: Irbesartan Tablet | CUA |
| No.16: CHEN Zijia, 2024 | China | Institute of Basic Research in Clinical Medicine, China Academy of Chinese Medical Sciences | Annao Pill | Primary Hypertension | Model-Based Study | A: Annao Pill plus Conventional Standard Treatment  B: Conventional Standard Treatment | CEA |
| No.17: CHENG Lili, 2022 | China | Jiangsu Pharmaceutical Industry Association | Houtou Jianweiling Tablet, Gastroparesis Granule | Chronic Gastritis | Model-Based Study | A: Houtou Jianweiling Tablet  B: Gastroparesis Granule | CEA+CUA |
| No.18: PAN Huimin, 2024 | China | Dept.of Pharmacy The First Affiliated Hospital of Xinjiang Medical University | Qili Qiangxin Capsule | Chronic Heart Failure | Model-Based Study | A: Qili Qiangxin Capsule plus Sacubitril Valsartan Sodium Tablet  B: Sacubitril Valsartan Sodium Tablet | CUA |
| No.19: Kai Xu, 2025 | China | Dept.of Pharmacy, The Second People's Hospital of Changzhou, The Third Affiliated Hospital of Nanjing Medical University | Marsdenia Tenacissima Injection, Aidi Injection, Kanglaite Injection, Kang Ai Injection, Shenqi Fuzheng Injection | NSCLC | Model-Based Study | A: Marsdenia Tenacissima Injection plus Chemotherapy vs. Chemotherapy  B: Aidi Injection plus Chemotherapy vs. Chemotherapy  C: Kanglaite Injection plus Chemotherapy vs. Chemotherapy  D: Kang Ai Injection plus Chemotherapy vs. Chemotherapy  E: Shenqi Fuzheng Injection plus Chemotherapy vs. Chemotherapy | CEA |
| No.20: LIANG Liang, 2024 | China | Dept.of Medicine, Faculty of Chinese Medicine Science Guangxi University of Chinese Medicine | Compound Biejia Rugan Tablet | Chronic Hepatitis B | Model-Based Study | A: Compound Biejia Rugan Tablets combination with Entecavir  B: Entecavir | CUA |
| No.21: WAN Feng, 2023 | China | State Key Laboratory of Southwestern Chinese Medicine Resources, Chengdu University of Traditional Chinese Medicine | Motherwort Injection | Pregnancy, Childbirth, and Postpartum Disorders | Prospective Cohort Study | A: Motherwort injection plus Oxytocin  B: Oxytocin | CEA+CUA |
| No.22: ZHANG XinYing, 2024 | China | School of Management, Beijing University of Chinese Medicine | Huashibaidu Granule | Coroma Virus Disease 2019 | Retrospective Study | A: Hua Shi Bai Du Granule plus Conventional Standard Treatment and Monitoring  B: Compound Fucidin Oral Solution plus Conventional Standard Treatment and Monitoring | CEA |
| No.23: LIANG wanxian, 2022 | China | Center for Evidence-Based Chinese Medicine, Beijing University of Chinese Medicine | Fufang Banmao Capsule, Yangzheng Xiaoji Capsule, Huai'er Granule | Primary Liver Cancer | Model-Based Study | A: Fufang Banmao Capsule plus Transarterial Chemoembolization (TACE)  B: Yangzheng Xiaoji Capsule plus TACE  C: Huai'er Granule plus TACE  D: TACE | CEA |
| No.24: ZHAO Yifan, 2022 | China | North China University of Science and Technology | Brucea Javanica Injection | Colorectal Cancer | Model-Based Study | A: Brucea Javanica Injection plus FOLFOX vs. FOLFOX  B: Brucea Javanica Injection plus CapeOX vs.CapeOX  C: Brucea Javanica Injection plus FOLIRI vs. FOLIRI | CEA |
| No.25: Yuliang Xiang, 2021 | China | Pharmaceutical Policy and Pharmacoeconomics Research Center, Sichuan University West China School of Pharmacy | Ginkgolide Injection | Ischemic Stroke | Model-Based Study | A: Ginkgolide Injection  B: Placebo | CUA |
| No.26: Lisong Yang, et al. 2020 | China | State Key Laboratory of Quality Research in Chinese Medicine, Institute of Chinese Medical Sciences, University of Macau | Salvianolate Injection, Danhong Injection, Alprostadil Injection | Coronary Heart Disease | Retrospective Study | A: Salvianolate Injection  B: Danhong injection  C: .Alprostadil injection | CEA |
| No.27: Meng-Bin Tang, 2024 | Taiwan, China | Dept.of Family Medicine, China Medical University Hospital, Taichung, Taiwan | Not Specified | NSCLC | Retrospective Study | A: Western Medicine Treatment plus Complementary TCM Treatment  B: Western Medicine Treatment | CEA |
| No.28: Gordon Liu, 2021 | China | Institute for Global Health and Development, Peking University | Xiaoke Pill | Type 2 Diabetes | Prospective Cohort Study | A: Xiaoke Pill  B: Metformin  C: Gliclazide  D:Acarbose  E:Glibenclamide  F:Other TCM | CEA+CUA |
| No.29: ZHAN Xudan, 2024 | China | Ningde Traditional Chinese Medicine Hospital of Fujian Province | Qizejupi Decoction and Granule | Chronic Heart Failure | Retrospective Study | A: Qizejupi Decoction  B: Qizejupi Granule | CMA |
| No.30: CAO ShiHuan, 2024 | China | Center for Evidence-Based Medicine, Beijing University of Chinese Medicine | Lianhua Qingwen Capsule (Granule),  Jinhua Qinggan Granule, Xuanfei Baidu Decoction | Novel Coronavirus Pneumonia | Model-Based Study | A: Lianhua Qingwen Capsule plus Conventional Standard Treatment  B: Jinhua Qinggan Granule plus Conventional Standard Treatment  C: Xuanfei Baidu Decoction plus Conventional Standard Treatment  D: Conventional Standard Treatment | CEA |
| No.31: Wei Zhongyi, 2024 | China | Institute of Basic Research in Clinical Medicine, China Academy of Chinese Medical Sciences | Jinye Baidu Granule, Compound LonICERa Granule | Acute Upper Respiratory Tract Infection | Model-Based Study | A: Jinye Baidu Granule  B: Compound LonICERa Granule | CEA |
| No.32: DAI Yingying, 2023 | China | Dept.of Pharmacy, Affiliated Ninth Hospital of Soochow University | Ginkgo Biloba Oral Preparation (G.biloba oral) | Cerebral Ischemic Stroke | Model-Based Study | A: G.biloba Oral Preparation plus Conventional Standard Treatment  B: Conventional Standard Treatment | CUA |
| No.33: YANG Shuo, 2023 | China | Institute of Basic Research in Clinical Medicine, China Academy of Chinese Medical Sciences | Xialiqi Capsule | Prostatic Hyperplasia with Deficiency in Origin and Excess in Superficies Syndrome | Model-Based Study | A: Xialiqi Capsule plus Conventional Standard Treatment  B: Conventional Standard Treatment | CEA |
| No.34: Ming Hu, 2022 | China | West China School of Pharmacy Sichuan University | Yupingfeng Granule | Acute Exacerbations of COPD | Model-Based Study | A: Yupingfeng Granule  B: Placebo | CUA |
| No.35: Qian Xu et al., 2020 | China | West China School of Pharmacy Sichuan University | Compound Apocynum Tablet | Hypertension | Model-Based Study | A: Compound Apocynum Tablet plus Nifedipine Extended-Release Tablet  B: Nifedipine Extended-Release Tablet | CEA+CUA |
| No.36: SUN Yang, 2024 | China | West China College of Pharmacy, Sichuan University | Huatuo Jiuxin Pill | Coronary Heart Disease | Model-Based Study | A: Huatuo Jiuxin Pill plus Conventional Standard Treatment  B: Conventional Standard Treatment | CUA |
| No.37: LI Mengyuan, 2025 | China | School of International Pharmaceutical Business, China Pharmaceutical University | Tribulus Terrestris Saponin Capsule, Xinnao Shutong Capsule | Windphlegm and Blood Stasis Obstruction Syndrome | Model-Based Study | A: Tribulus Terrestris Saponin Capsule  B: Xinnao Shutong Capsule | CUA |
| No.38: XIAO Guirong, 2021 | China | West China College of Pharmacy, Sichuan University, Dept.of Clinical Pharmacy, West China Hospital, Sichuan University | Chaishi Tuire Granule | Influenza | Prospective RCT | A: Chaishi-Tuire Granule  B: Oseltamivir Capsule | CMA |
| No.39: WEI Chunyan, 2022 | China | West China College of Pharmacy, Sichuan University | Shenshuaining Capsule | Chronic Renal Failure | Retrospective Study | A: Shenshuaining Capsule plus Conventional Standard Treatment  B: Conventional Standard Treatment | CEA |
| No.40: CAO ShiHuan, 2023 | China | Center for Evidence-Based Chinese Medicine, Beijing University of Chinese Medicine | Xiaoer Dingchuan Oral Liquid, Xiaoer Feire Kechuan Granule | Bronchial Asthma | Model-Based Study | A: Xiaoer Dingchuan Oral Liquid plus Budesonide  B: Xiaoer Feire Kechuan Granule plus Budesonide | CEA |
| No.41: LU Zhenkai, 2024 | China | Institute of Basic Research in Clinical Medicine,China Academy of Chinese Medical Sciences | Jiangzhi Tongluo Soft Capsule, Pushen Capsule | Hyperlipidemia | Model-Based Study | A: Jiangzhi Tongluo Soft Capsule  B: Pushen Capsule | CEA |
| No.42: LIU Teng, 2021 | China | Dept.of Pharmacy, Beijing Tiantan Hospital, Capital Medical University | Fufang Xueshuantong Dripping Pill (Capsule) | Retinal Vein Occlusion | Model-Based Study | A: Fufang Xueshuantong Dripping Pill  B: Fufang Xueshuantong Capsule | CUA |
| No.43: CUI Mancang, 2020 | China | Dept.of Pharmacy, the 989^th^ Hospital of Joint Logistics Support Unit | Salvia Miltiorrhiza Ligustrazine Injection, Danhong Injection | Unstable Angina Pectoris | Model-Based Study | A: Salvia Miltiorrhiza Ligustrazine Injection  B: Danhong Injection | CEA |
| No.44: LIANG WanXian, 2024 | China | Center for Evidence-Based Chinese Medicine, Beijing University of Chinese Medicine | Lianhua Qingwen Capsule | Adult Influenza | Model-Based Study | A: Lianhua Qingwen Capsule vs. Oseltamivir Phosphate Capsule  B: Lianhua Qingwen Capsule plus Oseltamivir Phosphate Capsule vs. Oseltamivir Phosphate Capsule | CEA |
| No.45: Pengli Su, 2024 | China | Institute of Basic Research in Clinical Medicine, China Academy of Chinese Medical Sciences | Danhong Injection (DHI) | Chronic Stable Angina | Model-Based Study | A: DHI plus Conventional Standard Treatment  B: Conventional Standard Treatment | CUA |
| No.46: CHEN Zijia, 2024 | China | Clinical Basic Medicine of Traditional Chinese Medicine, Chinese Academy of Traditional Chinese Medicine | Yiqing Capsule, Niuhuang Jiedu Capsule | Gingivitis (Heat-Toxin Syndrome) | Model-Based Study | A: Yiqing Capsule  B: Niuhuang Jiedu Capsule | CEA |
| No.47: LIU Huan, 2022 | China | Institute of Basic Research in Clinical Medicine, China Academy of Chinese Medical Sciences | Xiangju Capsule | Chronic Rhinosinusitis | Model-Based Study | A: Xiangju Capsule plus Conventional Standard Treatment  B: Conventional Standard Treatment | CEA |
| No.48: WANG Zhi-heng, 2021 | China | School of Management,Beijing University of Chinese Medicine | Suhuang Zhike Capsule | Acute Exacerbation Requiring Hospitalization, Stable Period Without Hospitalization, Death | Model-Based Study | A: Suhuang Zhike Capsule plus Conventional Standard Treatment  B: Conventional Standard Treatment | CUA |
| No.49: GUO ZhaoTing, 2021 | China | West China Shool of Pharmacy, Sichuan University | Ginkgolide Injection, Shuxuening Injection | Cerebral Infarction | Model-Based Study | A: Ginkgolide Injection  B: Shuxuening Injection | CEA+CUA |
| No.50: Hu Haiyao, 2022 | China | West China School of Medicine, Sichuan  University | Qinxiang Qingjie Oral Liquid | Upper Respiratory Tract Infection | Retrospective Study | A: Qinxiang Qingjie Oral Liquid plus Ribavirin Injection  B: Ribavirin Injection | CEA |
| No.51: LI Han, 2020 | China | Dongguan Traditional Chinese Medicine Hospital | Shuxuening Injection, Ginkgo, Biloba Extract Injection, Ginkgo Biloba Extract Injection (Ginaton) | Sudden Deafness | Retrospective Study | A:Shuxuening Injection (Xingxue)  B: Ginkgo Biloba Extract Injection  C: Ginkgo Biloba Extract Injection (Ginaton) | CEA |
| No.52: SHI Suisui, 2023 | China | Dongfang Hospital of Beijing University of Chinese Medicin | Sisang Ming Mu Decoction | Wet Age-Related Macular Degeneration | Prospective Cohort Study | A: Sisang Ming Mu Decoction plus Anti-angiogenic Drug  B: Anti-angiogenic Drug plus Placebo | CEA |
| No.53: WANG Zhuojue, 2022 | China | One Ward of Pediatric, Affiliated Hospital of Yan'an University | Xiaoer Douqiao Qingre Granule, Xiaoer Magan Granule | Mycoplasma Pneumonia | Retrospective Study | A: Xiaoer Douqiao Qingre Granule plus Azithromycin vs. Azithromycin  B: Xiaoer Magan Granule plus Azithromycin vs. Azithromycin | CEA |
| No.54: ZHOU Li, 2020 | China | West China School of Pharmacy, Sichuan  University | Ginkgolide Injection | Ischaemic Stroke of Large-Artery Atherosclerosis | Retrospective Study | A: Ginkgolide Injection plus Aspirin  B: Diphenhydramine Injection plus Aspirin | CMA |
| No.55: Xiang Gao, 2024 | China | Dept.of Urology, The Second Affiliated Hospital of Nanjing Medical University | Flavonoids of Desmodium Styracifolium (TFDS) | Urolithiasis | Model-Based Study | A: TFDS  B: Placebo | CEA |
| No.56: Sun Yan, 2025 | China | Dept.of Pharmacy, Zhengzhou Orthopaedic Hospital | Jinwugutong Capsule | Primary Osteoporosis | Prospective Cohort Study | A: Jinwugutong Capsule plus Conventional Standard Treatment  B: Conventional Standard Treatment | CEA |
| No.57: XIA Ruyu, 2022 | China | Centre for Evidence-based Chinese Medicine, Beijing University of Chinese Medicine, International Institute of Evidence-based Traditional Chinese Medicine, Beijing University of Chinese Medicine | Jinghuaweikang Capsule, Weisu Granule, Qizhiweitong Granule, Anweiyang Capsule, Jiawei Zuojin Pill, Xiangsha Liujunzi Pill, Zhizhu Kuanzhong Capsule | Helicobacter Pylori Related Peptic Ulcer And Gastritis | Model-Based Study | A: Jinghuaweikang Capsule plus Triple Therapy  B: Weisu Granule plus Triple Therapy  C: Qizhiweitong Granule plus Triple Therapy  D: Anweiyang Capsule plus Triple Therapy  E: Jiawei Zuojin Pill plus Triple Therapy  F: Xiangsha Liujunzi Pill plus Triple Therapy  G: Zhizhu Kuanzhong Capsule plus Triple Therapy  H: Triple Therapy | CEA |
| No.58: LEI Chao, 2022 | China | Institute of Basic Research in Clinical Medicine,China Academy of Chinese Medical Sciences | Xianyu Capsule | Epilepsy | Model-Based Study | A: Xianyu Capsule plus Magnesium Valproate Sustained-Release Tablet  B: Magnesium Valproate Sustained-Release Tablet | CEA |
| No.59: HU Xingmao, 2020 | China | Hebei Medical University | Yiqifumai Injection (Lyophilization), Xinmailong Injection | Chronic Heart Failure | Model-Based Study | A: Yiqifumai Injection (Lyophilization)  B: Xinmailong Injection | CMA |
| No.60: SHENG Ye, 2021 | China | Dept.of Pharmacy,Liyang People's Hospital,Jiangsu Province | Liqihuoxue Dripping Pill, Compound Danshen Dripping Pill | Chronic Stable Angina Pectoris | Model-Based Study | A: Liqihuoxue Dripping Pill  B: Compound Danshen Dripping Pill | CEA+CUA |
| No.61: ZHANG Lidan, 2022 | China | Institute of Basic Clinical Medicine of Traditional Chinese Medicine, China Academy of Chinese Medical Sciences | Mudan Granule | Diabetic Peripheral Neuropathy | Model-Based Study | A: Mudan Granule plus Mecobalamine, Eparstat, α-lipoic acid and other Western Medicine  B: Western Medicine | CEA |
| No.62: ZHENG Yingying, 2021 | China | The Third Hospital of Hebei Medical University, Shijiazhuang, Hebei | Compound Danshen Dripping Pill, Isosorbide Dinitrate Tablet | Stable Angina Pectoris | Model-Based Study | A: Compound Danshen Dripping Pill  B: Isosorbide Dinitrate Tablet | CEA |
| No.63: ZHANG Shiqin, 2025 | China | Boya Intelligent Health Research Institute | Dachuanxiong Oral Liquid, Tongtian Oral Liquid | Convalescent Stroke | Model-Based Study | A: Dachuanxiong Oral Liquid plus Conventional Standard Treatment  B: Tongtian Oral Liquid plus Conventional Standard Treatment | CEA |
| No.64: LI Wei, 2021 | China | International Research Center for Medicinal Administration, Peking University | Dengzhan Shengmai Capsule (DZSM) | Secondary Stroke Prevention | Model-Based Study | A: DZSM Capsule plus Conventional Standard Treatment  B: Conventional Standard Treatment | CUA |
| No.65: WANG Zhiheng, 2021 | China | School of Management, Beijing University of Chinese Medicine | Zhishe Tongluo Capsule, Naoan Capsule | Apoplexy (Qi Deficiency and Blood Stasis Syndrome) | Model-Based Study | A: Zhishe Tongluo Capsule  B: Naoan Capsule | CUA |
| No.66: KOU Zhenzhen, 2023 | China | Traditional Chinese Medicine Pharmacy, Suixian Traditional Chinese Medicine Hospital | Qingkailing Injection, Shuanghuanglian Injection, Yanhuning Injection, Xiyanping Injection | Acute Viral Myocarditis | Retrospective Study | A: Qingkailing Injection  B: Shuanghuanglian Injection  C: Yanhuning Injection  D: Xiyanping Injection | CEA |
| No.67: GUO Xiaojie, 2020 | China | Dept.of Internal Medicine,Taian Second Hospital of Traditional Chinese Medicine,Shandong Province | Bushen Zhuyu Huatan Decoction, Xuesaitong Capsule | Hypertensive Cerebral Hemorrhage | Retrospective Study | A:Bushen Zhuyu Huatan Decoction plus Conventional Standard Treatment  B: Xuesaitong Capsule plus Conventional Standard Treatment | CEA |
| No.68: ZHEN Hui, 2021 | China | Drug Research and Evaluation Technology Center, China Traditional Chinese Medicine Association | Shuangdong Capsule | Infection of Dampness-Heat | Prospective RCT | A: Shuangdong Capsule  B: Western Medicine | CEA+CUA |
| No.69: LI Xuan, 2022 | China | Clinical Trial Center, First Teaching Hospital of Tianjin University of Traditional Chinese Medicine/National Clinical Research  Center for Chinese Medicine Acupuncture and Moxibustion | Xiaoer Resuqing Syrup, Xiaoer Qingrening Granule | Upper Respiratory Tract Infection | Retrospective Study | A: Xiaoer Resuqing Syrup plus Xiaoer Qingrening Granule Simulator  B: Xiaoer Qingrening Granule plus Xiaoer Resuqing Syrup Simulator | CEA |
| No.70: GAN Xiong, 2023 | China | Dept.of Clinical Pharmacy, Hanchuan People's Hospital | Qili Qiangxin Capsule | Chronic Heart Failure  and Reduced Rjection Fraction | Retrospective Study | A: Qili Qiangxin Capsule plus Conventional Standard Treatment  B: Conventional Standard Treatment plus Placebo | CEA |
| No.71: LIU Zhaohui, 2024 | China | Dept.of Gynecology, Beijing Obstetrics and Gynecology Hospital, Capital Medical University, Beijing Maternal and Child Health Care Hospital | Kangfuyan Capsule, Gongyanping Capsule | Pelvic Inflammatory Disease Sequelae (SPID) -Chronic Pelvic Pain (CPP) | Prospective RCT | A: Kangfuyan Capsule  B: Gongyanping Capsule | CEA+CUA |
| No.72: CUI Xin, 2023 | China | Institute of Basic Research in Clinical Medicine,China Academy of Chinese Medical Sciences | Jintiange Capsule, Gushukang Capsule | Primary Osteoporosis (Liver  And Kidney Deficiency Syndrome) | Model-Based Study | A: Jintiange Capsule  B: Gushukang Capsule | CEA |
| No.73: WEI Ruili, 2020 | China | Institute of Basic Research in Clinical Medicine,China Academy of Chinese Medical Sciences | Qidong Yixin Oral Liquid | Viral Myocarditis (Qi-Yin Deficiency Syndrome) | Model-Based Study | A: Qidong Yixin Oral Liquid plus Conventional Standard Treatment  B: Conventional Standard Treatment | CEA |
| No.74: CUI Xin, 2023 | China | Institute of Basic Research in Clinical Medicine,China Academy of Chinese Medical Sciences | Qianggu Capsule | Primary Osteoporosis | Model-Based Study | A: Qianggu Capsule plus Alendronate Sodium Tablet  B: Alendronate Sodium Tablet | CEA |
| No.75: CUI Xin, 2022 | China | Institute of Basic Research in Clinical Medicine, China Academy of Chinese Medical Sciences | Qilong Capsule | Ischemic Stroke | Model-Based Study | A: Qilong capsule plus Conventional Standard Treatment  B: Conventional Standard Treatment | CEA |
| No.76: LIU Jifang, 2023 | China | Beijing Rongchang Pharmaceutical Research Institute Co., Ltd. | Tianmeng Oral Liquid, Wuling Capsule | Insomnia | Model-Based Study | A: Tianmeng Oral Liquid  B: Wuling Capsule | CEA |
| No.77: WANG Fangxu, 2023 | China | Institute of Health Policy and Technology Assessment, Health Science Center, Peking University | Jingutongxiao Pill, Guci Tablet | Knee Osteoarthritis | Model-Based Study | A: Jingutongxiao Pill  B: Guci Tablet  C: Diclofenac Sodium Sustained-release Tablet | CEA |
| No.78: PAN Jie, 2021 | China | School of Management,Beijing University of Chinese Medicine | Shexiang Tongxin Dripping Pill | Coronary heart Disease | Model-Based Study | A: Shexiang Tongxin Dripping Pill plus Conventional Standard Treatment  B: Conventional Standard Treatment | CUA |
| No.79: CUI Xin, 2023 | China | Institute of Basic Research in Clinical Medicine,China Academy of Chinese Medical Sciences | Yinhuang Qingfei Capsule | Acute Attack of  Chronic Bronchitis | Model-Based Study | A: Yinhuang Qingfei Capsule plus Conventional Standard Treatment  B: Conventional Standard Treatment | CEA |
| No.80: XU Haiting, 2024 | China | Fuqing City Hospital of Fujian | Xue Saitong Soft Capsule | Ischemic Stroke | Model-Based Study | A: Xue Saitong Soft Capsule  B: Placebo | CUA |
| No.81: LI Fan, 2020 | China | Dazu District People's Hospital | Lishi Buqi Decoction, Yinzhihuang Oral Liquid | ABO Hemolytic Disease | Retrospective Study | A: Lishi Buqi Decoction  B: Yinzhihuang Oral Liquid | CEA |
| No.82: LIU Xing, 2020 | China | Dept.of Pharmacy Kunming Third People's Hospital | Bufei Decoction | MDR-TB | Retrospective Study | A: Bufei Decoction plus Conventional Standard Treatment  B: Conventional Standard Treatment | CEA |
| No.83: Ma Luyao, 2023 | China | Tianjin University of Traditional Chinese Medicine | Shensong yangxin Capsule | Coronary Heart Disease Arrhythmia | Retrospective Study | A: SSYX vs. Metoprolol  B: SSYX vs. Amiodarone | CEA |
| No.84: LIU Dan, 2022 | China | Dept.of Pharmacy/Evidence-Based Pharmacy Center, West China Second University Hospital | Danmu Extract Syrup, Xiaoer Chiqiao Qingre Granule | Acute Upper Respiratory Tract Infection | Prospective Cohort Study | A: Danmu Extract Syrup plus Conventional Standard Treatment  B: Xiaoer Chiqiao Qingre Granule plus Conventional Standard Treatment | CEA |
| No.85: WANG Yaqin, 2021 | China | Dept.of Pharmacy, Henan Provincial People's Hospital | Compound Kushen Injection (CKI) | Colorectal Cancer | Retrospective Study | A: CKI plus FOLFOX  B: FOLFOX | CEA |
| No.86: XU Longchen, 2022 | China | Beijing University of Chinese Medicine, School of management | Simotang Oral Liquid | Gastrointestinal Functional Diseases and Jaundice | Retrospective Study | A: Simotang Oral Liquid vs. Domperidone Suspension  B: Simotang Oral Liquid plus Conventional Standard Treatment vs. Yinzhi Huang Oral Liquid plus Conventional Standard Treatment  C: Simotang Oral Liquid plus Conventional Standard Treatment vs. Conventional Standard Treatment | CEA+CBA |
| No.87: MA Luyao, 2022 | China | Baokang Hospital Affiliated to Tianjin University of Traditional Chinese Medicine | Shensong Yangxin Capsule | Ventricular Premature Beat | Retrospective Study | A: Shensong Yangxin Capsule plus Metoprolol vs. Metoprolol  B: Shensong Yangxin Capsule vs. Metoprolol  C: Shensong Yangxin Capsule vs. Propafenone | CEA |
| No.88: Lian Tang, 2022 | China | Nantong Municipal First People's Hospital | Ginko Leaf Extract | Angina Pectoria of Cornary Heart Disease | Retrospective Study | A: Ginko Leaf Extract plus Conventional Standard Treatment  B: Conventional Standard Treatment | CEA |
| No.89: ZHANG XinYing, 2024 | China | School of Management, Beijing University of Chinese Medicine | Huashi Baidu Granule | Coroma Virus Disease  2019 | Model-Based Study | A: Huashi Baidu Granule plus Conventional Standard Treatment  B: Conventional Standard Treatment | CUA |
| No.90: WANG Xiaoli, 2023 | China | Dept of Pharmacy, the Third Hospital of Hebei Medical University | Compound Danshen Dripping Pill, Shexiang Baoxin Pill, Naoxintong Capsule | Stable Angina Pectoris | Model-Based Study | A: Compound Danshen Dripping Pill plus Conventional Standard Treatment  B: Shexiang Baoxin Pill plus Conventional Standard Treatment  C: Naoxintong Capsule plus Conventional Standard Treatment | CUA |
| No.91: ZHU Longxun, 2024 | China | Dept.of pharmacy, The Second Affiliated Hospital of Nantong University | Sanjin Tablet | Urinary Tract Infection | Model-Based Study | A: Sanjin Tablet plus Antibiotic  B: Antibiotic | CEA |
| No.92: HOU Xiaohua, 2024 | China | Dept.of Gastroenterology, Union Hospital Affiliated to Tongji Medical College, Huazhong University of Science and Technology | Tongxiening Granule | Diarrheal Irritable Bowel  Syndrome | Model-Based Study | A: Tongxiening Granule plus Piverium Bromide Tablets Simulator  B: Piverium Bromide Tablet plus Tongxiening Granule Simulator | CUA |
| No.93: LI Junhui, 2025 | China | Shangqiu First People's Hospital Shangqiu | HuoBaHuaGen Pill, KunMingShanHaiTang Pill | Rheumatoid Arthritis | Model-Based Study | A: HuoBaHuaGen Pill  B: KunMingShanHaiTang Pill | CUA |
| No.94: LIU Chang, 2022 | China | Center for Health Policy and Technology Evaluation, Peking University Health Science Center | Keluoxin Capsule | Diabetic Kidney Disease | Model-Based Study | A: Keluoxin Capsule plus Chemotherapy  B: Chemotherapy | CUA |
| No.95: LI Menglan, 2020 | China | Inner Mongolia Autonomous Region Hospital of Traditional Chinese | Ginkgolide Injection, Xueshuantong Injection, Danhong Injection | Cerebral Infarction | Retrospective Study | A: Ginkgolide Injection  B: Xueshuantong Injection  C: Danhong Injection | CEA |
| No.96: ZHANG Huiling, 2020 | China | Dept.of Traditional Chinese Medicine, The Third Hospital of Hebei Medical University | Not Specified | Irregular Menstruation | Retrospective Study | A: Chinese Herbal Decoction Pieces  B: Chinese Herbal Formula Granule | CMA |
| No.97: Yang Guangyao, 2024 | China | Beijing Hepingli Hospital | Fufang Huangbai (FFHB) Fluid Hydropathic Compress | Diabetic Foot Infections | Prospective RCT | A: FFHB Hydropathic Compress  B: Antimicrobial Calcium Alginate Wound Dressing (ACAWD) | CEA |
| No.98: JIANG Qiyun, 2025 | China | The Third Affiliated Hospital, Southern Medical University | Tripterygium Glycosides Tablets (TGT) | Rheumatoid Arthriti | Model-Based Study | A:TGT plus with Methotrexate  B: Methotrexate plus sulfasalazine plus hydroxychloro | CUA |
| No.99: LI Xuejing, 2021 | China | Dept.of Pharmacy,the Third Hospital of Hebei Medical University | Compound Danshen Dripping Pill, Shexiang Baoxin Pill | Unstable Angina Pectoris | Retrospective Study | A: Compound Danshen Dripping Pill plus Conventional Standard Treatment  B: Shexiang Baoxin Pill plus Conventional Standard Treatment | CMA |
| No.100: WEI Jiayin, 2023 | China | School of Management, Beijing University of Traditional Chinese Medicine | Danshu Capsule, Jindan Tablet, Xiaoyanlidan Tablet | Chronic Cholecystitis, Chronic Calculous Cholecystitis | Retrospective Study | A: Danshu Capsule  B: Jindan Tablet  C: Xiaoyanlidan Tablet  D: Ursodeoxycholic Acid Tablet  E: Danning Tablet | CEA |
| No.101: HE Yumei, 2024 | China | International Research Center for Medicinal Administration, Peking University | Bailing Capsule | Chronic Renal Failure | Model-Based Study | A: Bailing Capsule plus Conventional Standard Treatment  B: Conventional Standard Treatment | CEA |
| No.102: YANG Daowen, 2025 | China | Dept.of Traditional Chinese Medicine Pulmonology I, China-Japan Friendship Hospital | Guben Kechuan Granule, Guilong Kechuanning Capsule | Chronic Bronchitis | Retrospective Study | A: Guben Kechuan Granule  B: Guilong Kechuanning Capsule  C: Placebo | CEA+CMA |
| No.103: Zou Hong, 2025 | China | Dept.of Pharmacy, the Second Hospital of Dalian Medical University | Xingnaojing Injection, Shuxuetong Injection | Cerebral Infarction | Retrospective Study | A: Xingnaojing Injection plus Conventional Standard Treatment  B: Shuxuetong Injection plus Conventional Standard Treatment | CEA |
| No.104: LIU Guoqiang, 2021 | China | Dept of Pharmacy,the Third Hospital of Hebei Medical University | Tongluo Kaibi Tablet,  Biqi Capsule, Gulong Capsule | Rheumatoid Arthritis | Model-Based Study | A: Tongluo Kaibi Tablet plus Methotrexate  B: Biqi Capsule plus Methotrexate  C: Gulong Capsule plus Methotrexate | CEA |
| No.105: LI Kebiao, 2022 | China | Hospital Management Institute of Wuhan University | Qizhi Weitong Granule | Functional Dyspepsia | Model-Based Study | A: Qizhi Weitong Granule plus Mosapride  B: Mosapride | CEA |
| No.106: CUI Xin, 2023 | China | Institute of Basic Research in Clinical Medicine,China Academy of Chinese Medical Sciences | Tianma Gouteng Granule, (TGG) | Essential  Hypertension | Model-Based Study | A: TGG, Nifedipine Controlled Released Tablet  B: Nifedipine Controlled Released Tablet | CEA |
| No.107: HU Yuer, 2023 | China | Institute of Basic Research in Clinical Medicine,China Academy of Chinese Medical Sciences | Compound Danshen Spray, Compound Danshen Tablet | Angina Pectoria of Cornary Heart Disease | Model-Based Study | A: Compound Danshen Tablet  B: Compound Danshen Spray | CEA |
| No.108: WANG Guozhen, 2020 | China | The First Affiliated Hospital of Chongqing Medical University | Yangxueqingnao  Granule | Migraine | Model-Based Study | A: Flunarizine Hydrochloride Capsule plus Yangxueqingnao Granule  B: Flunarizine Hydrochloride Capsule | CEA |
| No.109: WANG Xianying, 2021 | China | Dept.of Pharmacy, the Third Hospital of Hebei Medical University | Compound Danshen Dripping Pill, Shexiang Baoxin Pill | Angina Pectoria of Cornary Heart Disease | Model-Based Study | A: Compound Danshen Dripping Pill  B: Shexiang Baoxin Pill | CEA |
| No.110: LI Yunhui, 2025 | China | Dept.of Respiratory and Critical Care Medicine, the Second Affiliated Hospital of Tianjin University of Traditional Chinese Medicine | Shiweilongdanhua Capsule, Feilike Capsule | Acute Tracheo-bronchitis (Phlegm-heat Congestion of the Lungs Syndrome) | Model-Based Study | A: Shiweilongdanhua Capsule  B: Feilike Capsule | CEA |
| No.111: LIU Guoqiang, 2020 | China | Dept.of Pharmacy, the Third Hospital of Hebei Medical University | Not Specified | Nephrotic Syndrome | Retrospective Study | A: Decoction  B: Granule | CMA |
| No.112: ZHENG Qinxin, 2021 | China | Dept.of Pharmacy,the Second People's Hospital Affiliated to Fujian University of Traditional Chinese Medicine | Majing Cough Granule | Adult Non-severe Community-acquired Pneumonia | Retrospective Study | A: Moxifloxacin Injection plus Majing Cough Granule  B: Moxifloxacin Injection | CMA |
| No.113: LU Yunfei, 2021 | China | Dept.of traditional Chinese Medicine, Shanghai Public Health Clinical Center | Kuhuang Granule, Yinzhihuang Oral Liquid, Yinzhihuang Granule | Jaundice Viral Hepatitis | Retrospective Study | A: Kuhuang Granule plus Conventional Standard Treatment  B: Yinzhihuang Oral Liquid plus Conventional Standard Treatment  C: Yinzhihuang Granule plus Conventional Standard Treatment | CEA |
| No.114: ZHOU Ruiyue, 2023 | China | Dept.of Hepatology, Shanxi Provincial Hospital of Traditional Chinese Medicine | Kuhuang Injection, Shuganning Injection | Jaundice Viral Hepatitis | Retrospective Study | A: Kuhuang Injection  B: Shuganning Injection | CMA |
| No.115: CHEN Yu, 2025 | China | School of International Pharmaceutical Business,China Pharmaceutical University | Jiuwei Zhike Oral Liquid, Jizhi Syrup | Acute Bronchitis | Retrospective Study | A: Jiuwei Zhike Oral Liquid  B: Jizhi Syrup | CEA |
| No.116: DING Liman, 2023 | China | Zhejiang Chinese Medical University | Chaiyin Granule | Influenza | Prospective RCT | A: Chaiyin Granule and Oseltamivir Phosphate Capsule Simulator  B: Oseltamivir Phosphate Capsule and Chaiyin Granule Simulator | CEA |
| No.117: CHEN Zhongguo, 2023 | China | Guiyang Xintian Pharmaceutical Co., Ltd. Shanghai Haitian Pharmaceutical Technology Development Co., Ltd. | Ningmitai Capsule, Longqing Tablet, Sanjin Tablet, Shuangshi Tonglin Capsule, Qianlie Shutong Capsule, Qianlie Beixi Capsule | Chronic Prostatitis | Model-Based Study | Single-use α-receptor blockers (Tamsulosin), Antibiotics (Levofloxacin), Ningmitai Capsule and Ningmitai Capsule combination with the above drugs in the treatment | CEA+CMA |
| No.118: QI Ran, 2022 | China | Dept.of Pharmacy, The Third Hospital of Hebei Medical University | Tianma Xingnao Capsule, Compound Congrong Yizhi Capsule | Vascular Diseases | Model-Based Study | A: Tianma Xingnao Capsule  B: Compound Congrong Yizhi Capsule | CEA+CMA |
| No.119: LI Jintian, 2022 | China | College of Public Health, Xinjiang Medical University | Xiaoer Xiaoji Zhike Oral Liquid, Xiaoer Feire Kechuan Oral Liquid, Xiaoer Chiqiao Qingre Granule, Xiaoer Feike Granule, Jinzhen Oral Liquid, Xiaoer Feire Kechuan Granule | Mycoplasma Pneumonia | Model-Based Study | A: Xiaoer Xiaoji Zhike Oral Liquid plus Azithromycin vs. Azithromycin  B: Xiaoer Feire Kechuan Oral Liquid plus Azithromycin vs. Azithromycin  C: Xiaoer Chiqiao Qingre granule plus Azithromycin vs. Azithromycin  D: Xiaoer Feike Granule plus Azithromycin vs. Azithromycin  E: Jinzhen Oral Liquid plus Azithromycin vs. Azithromycin  F: Xiaoer Feire Kechuan Granule plus Azithromycin vs. Azithromycin | CEA |
| No.120: WANG Fuping, 2024 | China | Institute of Basic Research in Clinical Medicine,China Academy of Chinese Medical Sciences | Kuntai Capsule | Menopausal Syndrome | Model-Based Study | A: Kuntai Capsule plus Hormone Therapy  B: Hormone Therapy | CEA |
| No.121: WANG Fuping, 2024 | China | Institute of Basic Research in Clinical Medicine,China Academy of Chinese Medical Sciences | Yupingfeng Capsule | Stable Chronic Obstructive Pulmonary Disease | Model-Based Study | A: Yupingfeng Capsule plus Conventional Standard Treatment  B: Conventional Standard Treatment | CEA |
| No.122: CUI Xin, 2022 | China | Institute of Basic Research in Clinical Medicine,China Academy of Chinese Medical Sciences | Qilong Capsule | Cerebral Ischemic Stroke | Model-Based Study | A: Qilong Capsule plus Conventional Standard Treatment  B: Conventional Standard Treatment | CEA |
| No.123: DU Guiping, 2022 | China | The Third Hospital of Hebei Medical University | Tenghuangjiangu Capsule | Osteoporosis | Model-Based Study | A: Tenghuangjiangu Capsule plus Conventional Standard Treatment  B: Conventional Standard Treatment | CEA |
| No.124: CUI Xin, 2023 | China | Institute of Basic Research in Clinical Medicine,China Academy of Chinese Medical Sciences | Guanxin Shutong Capsule | Coronary Heart Disease Angina Pectoris | Model-Based Study | A: Guanxin Shutong Capsule plus Conventional Standard Treatment  B: Conventional Standard Treatment | CEA |
| No.125: ZHAO Dongming, 2024 | China | Dept.of Otolaryngology, Luoyang First People's Hospital | Ganqingre Tablet, Jingfang Mixture, Gegen Tang Granule | Colds | Model-Based Study | A: Ganqingre Tablet vs. Jingfang Mixture  B: Ganqingre Tablet vs. Gegen Tang Granule | CEA |
| No.126: WEI Zhongyi, 2024 | China | Institute of Basic Research in Clinical Medicine, China Academy of Chinese Medical Sciences | Luobufukebiri Pill | Asthenospermia | Model-Based Study | A: Luobufukebiri Pill  B: Vitamin E Soft Capsule | CEA |
| No.127: ZHANG Yanling, 2025 | China | Xiyuan Hospital, China Academy of Chinese Medical Sciences | Zhisang Capsule, Orthopedic Bone Tablet | Acute Soft Tissue Injuries | Model-Based Study | A: Zhisang Capsule  B: Orthopedic Bone Tablet | CEA |
| No.128: XU Longchen, 2025 | China | Aerospace 731 Hospital | Qiming Granule | Diabetic Retinopathy | Model-Based Study | A: Qiming Granule plus Conventional Standard Treatment  B: Conventional Standard Treatment | CUA |
| No.129: SHAO Wei, 2025 | China | Liaoning Vocational College of Medicine, Liaoning Institute of Basic Medical Sciences | Xiaoer Feire Kechuan Granule | Influenza | Model-Based Study | A: Xiaoer Feire Kechuan Granule  B: Oseltamivir Phosphate Granule | CEA |
| No.130: CUI Xin, 2023 | China | Institute of Basic Research in Clinical Medicine,China Academy of Chinese Medical Sciences | Qianggu Capsule (QC) | Primary Osteoporosis | Model-Based Study | A: QC  B: Alfacalcidol Capsule (AC) | CEA |
| No.131: ZHANG Xuebin, 2021 | China | Professional Committee of Pharmacoeconomics of China Association of Traditional Chinese Medicine | Danlou Tablet | Stable Coronary Heart Disease | Prospective cohort study | A: Danlou Tablet plus Western Medicine  B: Western Medicine | CEA+CUA |
| No.132: HU Jing, 2020 | China | Dept.of Pharmacy,Shanghai Ruijin Rehabilitation Hospital | Danshen Injection, Danshen Polyphenols Injection, Shuxuetong Injection, Xuesaitong Injection, Kudi Injection, Shuxuening Injection | Cervical Spondylosis | Retrospective Study | A: Danshen Injection vs.Conventional Standard Treatment  B: Danshen Polyphenols Injection vs.Conventional Standard Treatment  C: Shuxuetong Injection vs.Conventional Standard Treatment  D: Xuesaitong Injection vs.Conventional Standard Treatment  E: Kudi Injection, Shuxuening Injection vs.Conventional Standard Treatment | CMA |
| No.133: LIU GuoQiang, 2020 | China | Dept.of Pharmacy, the Third Hospital of Hebei Medical University | Banxia Xiexin Decoction and Granule | Chronic Non-Atrophic Gastritis With Syndrome of Intermingled Heat And Cold | Retrospective Study | A: Banxia Xiexin Granule  B: Banxia Xiexin Decoction | CMA |
| No.134: LI Xuejing, 2020 | China | Dept.of Pharmacy, the Third Hospital of Hebei Medical University | Qishen Yiqi Dripping Pill | Chronic Heart Failure | Retrospective Study | A: Qishen Yiqi Dripping Pill plus Conventional Standard Treatment  B: Conventional Standard Treatment | CEA |
| No.135: YU Rong, 2023 | China | Pharmacy Dept.of Shanxi Cardiovascular Hospital | Shexiang Baoxin Pill, Compound Danshen Dripping Pill | Coronary Heart Failure | Model-Based Study | A: Shexiang Baoxin Pill  B: Compound Danshen Dripping Pill | CEA |
| No.136: LIU Fumei, 2021 | China | Institute of Basic Research in Clinical Medicine,China Academy of Chinese Medical Sciences | Diemailing^Ⓡ^Kudiezi Injection | Cerebral Infarction | Model-Based Study | A: Diemailing^Ⓡ^Kudiezi Injection plus Conventional Standard Treatment  B: Conventional Standard Treatment | CEA |
| No.137: CHEN Binbin, 2022 | China | Beijing Medical and Health Economic Research Association | Compound Qingdai Capsule (Pill) | Psoriasis Vulgaris | Model-Based Study | A: Compound Qingdai Capsule (Pill) plus Conventional Standard Treatment  B: Conventional Standard Treatment | CEA+CUA |
| No.138: CUI Peng, 2022 | China | Qing Dao University School of Medicine (First Clinical Medical College) | Not specified | NSCL | Retrospective Study | A: TCM plus Conventional Standard Treatment  B: Conventional Standard Treatment | CEA |
| No.139: GUAN Haijing, 2022 | China | Dept.of Pharmacy, Beijing Tiantan Hospital, Capital Medical University | Qili Qiangxin Capsule | Chronic Heart Failure | Model-Based Study | A: Qili Qiangxin Capsule plus Conventional Standard Treatment  B: Conventional Standard Treatment | CUA |
| No.140: LIN Sisi, 2024 | China | Office of Drug Clinical Trial Institution, Third Affiliated Hospital, Southern Medical University | Epimedium Total Flavone Capsule, Gusongbao Capsule | Postmenopausal Osteoporosis Fractures | Model-Based Study | A: Epimedium Total Flavone Capsule  B: Gusongbao Capsule | CUA |
| No.141: PENG Nan, 2024 | China | School of International Pharmaceutical Business, China Pharmaceutical University | Jintiange Capsule, Xianlinggubao Capsule | Postmenopausal Oseoporosis | Prospective Cohort Study | A: Jintiange Capsule plus Bisphosphonate  B: Xianlinggubao Capsule plus Bisphosphonate | CEA+CUA |
| No.142: LEI Xiang, 2020 | China | Beijing Qihuang Pharmaceutical Clinical Contract Research Organization | Wangbi Tablet | Rheumatoid Arthritis | Prospective RCT | A: Wangbi Tablet plus  Methotrexate Tablet  B: Methotrexate Tablet plus Wangbi Tablet Simulator | CEA |
| No.143: WANG Yulai, 2021 | China | Huangshi Maternity and Children's Health Hospital,Edong Healthcare Group | Xueshuantong Injection, Tianma Injection | Cerebrovascular Diseases | Retrospective Study | A: Xueshuantong Injection plus Tianma Injection  B: Xueshuantong Injection  C: Tianma Injection  D: Conventional Standard Treatment | CEA |
| No.144: HE Yumei, 2024 | China | International Research Center for Medicinal Administration, Peking University | Bailing Capsule | Diabetic Kidney Disease | Model-Based Study | A: Bailing Capsule plus Conventional Standard Treatment  B: Conventional Standard Treatment | CEA |
| No.145: XIANG Heng, 2025 | China | Dept.of Pharmacy, The Second Xiangya Hospital, Central South University | Jiegu Qili Capsule, Jiegu Qili Tablet | Postoperative Fracture | Model-Based Study | A: Jiegu Qili Capsule  B: Jiegu Qili Tablet | CEA |
| No.146: XU Manling, 2025 | China | Shengda Mingrui (Beijing) International Pharmaceutical Technology Co., LTD. | Tongluo Kaibi Tablets, Wangbi Capsule | Rheumatoid Arthritis | Model-Based Study | A: Tongluo Kaibi Tablet plus Conventional Standard Treatment  B: Wangbi Capsule plus Conventional Standard Treatment | CEA |
| No.147: CAI Jiarui, 2025 | China | West China Fourth Hospital, Sichuan University | Prunella Vulgaris | Thyroid Nodules | Model-Based Study | A: Prunella vulgaris plus Levothyroxine Sodium Tablet  B: Levothyroxine Sodium Tablet | CEA |
| No.148: HOU Hairuo, 2023 | China | Dept.of Pharmacy, Affiliated Shenzhen Maternity & Child Healthcare Hospital, Southern Medical University | Not specified | Missed Abortion In Early Pregnancy | Model-Based Study | A: Chinese Decoction Powder plus Mifepristone  B: Chinese Decoction Pieces plus Mifepristone | CMA |
| No.149: WANG Xin, 2024 | China | Beijing Ainusen Pharmaceutical Technology Co., LTD. | Ganjiebingmei Tablet, Qingyan Dripping Pill | Acute Pharyngitis | Model-Based Study | A: Ganjiebingmei Tablet vs. Qingyan Dripping Pill  B: Ganjiebingmei Tablet plus Conventional Standard Treatment vs. Qingyan Dripping Pill plus Conventional Standard Treatment | CEA |
| No.150: Zhang Hongyan, 2025 | China | Institute of Basic Research in Clinical Medicine,China Academy of Chinese Medical Sciences | Tianshu Capsule | Migraine | Model-Based Study | A: Tianshu Capsule plus Conventional Standard Treatment  B: Conventional Standard Treatment | CEA |
| No.151: HUANG Zhengming, 2025 | China | China Medical Education Association | Total Glucosides of White Paeony Capsule | Rheumatoid Arthritis | Model-Based Study | A: Total Glucosides of White Paeony Capsule  B: Hydroxychloroquine | CEA |
| No.152: XU Qian, 2022 | China | Second Affiliated Hospital,Heilongjiang University of Chinese Medicine | Xueshuantong Capsule, Ginkgo Biloba Capsule | Coronary Heart Disease (Blood Stasis Syndrome) | Prospective RCT | A: Xueshuantong Capsule  B: Ginkgo Biloba Capsule | CEA+CUA |
| No.153: LUO Ji, 2021 | China | Kunming Third People's Hospital Kunming | Not specified | Multidrug-Resistant Tuberculosis(Qi-Yin Deficiency Pattern) | Retrospective Study | A: Chinese Decoction Pieces  B: Chinese Herbal Formula Granule | CEA |
| No.154: ZHANG Xuebin, 2021 | China | Pharmaceutical economics committee of Chinese Traditional Medicine Association | Suxiaojiuxin Pill, Compound Danshen Dripping Pill, Shexiangbaoxin Pill | Coronary Heart Disease | Prospective Cohort Study | A: Suxiaojiuxin Pill  B: Compound danshen Dripping Pill  C: Shexiangbaoxin Pill | CEA+CUA |
| No.155: ZHENG Hang, 2023 | China | College of Pharmacy, Chongqing Medical University | Ginkgo Leaf Extract and Dipyridamole Injection, Compound Danshen Injection | Cerebral Infarction | Retrospective Study | A:Ginkgo Leaf Extract and Dipyridamole Injection plus Aspirin vs. Aspirin  B: Ginkgo Leaf Extract and Dipyridamole Injection plus Sodium Ozagrel Injection vs. Sodium Ozagrel Injection  C: Ginkgo Leaf Extract and Dipyridamole Injection plus Batroxobin vs. Batroxobin  D: Ginkgo Leaf Extract and Dipyridamole Injection plus Conventional Treatment vs. Conventional Treatment  E: Ginkgo Leaf Extract and Dipyridamole Injection plus Compound Danshen Injection vs. Compound Danshen Injection  F: Ginkgo Leaf Extract and Dipyridamole Injection plus Edaravone Injection vs. Edaravone Injection | CEA |
| No.156: PANG Hongbo, 2022 | China | Dept.of Neurology, Wuhu Second People's Hospital | Xueshuantong Capsule | Cardiovascular and Cerebrovascular Diseases | Prospective Cohort Study | A: Xueshuantong Capsule  B: Aspirin and Simvastatin | CMA |
| No.157: YANG Chunmei, 2020 | China | Dept.of Pharmacy, Yancheng First People's Hospital | Liqi Huoxue Dripping Pill | Chronic Stable Angina Pectoris | Model-Based Study | A: Liqi Huoxue Dripping Pill  B: Ranolazine | CUA |
| No.158: Zhou Bin, 2023 | China | Dept.of Endocrinology, The Affiliated Wujin Hospital of Jiangsu University | Ganshaung Granule | Hyperthyroidism with Liver Damage | Model-Based Study | A: Ganshaung Granule  B: Polyene Phosphatidylcholine | CEA |
| No.159: LI Chang, 2024 | China | Dept.of Pharmacy, Shijiazhuang Hospital of Traditional Chinese Medicine | Shexiang Baoxin Pill, Suxiao Jiuxin Pill | Coronary Heart Disease | Model-Based Study | A: Shexiang Baoxin Pill  B: Suxiao Jiuxin Pill | CEA |
| No.160: WANG Yizhu, 2024 | China | Institute of Basic Research in Clinical Medicine,China Academy of Chinese Medical Sciences | Compound Longxuejie Capsule, Compound Danshen Capsule | Stable Angina Pectoris with Coronary Atherosclerotic Heart Disease (Heart Blood Stasis Syndrome) | Model-Based Study | A: Compound Longxuejie Capsule  B: Compound Danshen Capsule | CEA |
| No.161: WANG XiaoYan, 2024 | China | Institute of Basic Research in Clinical Medicine,China Academy of Chinese Medical Sciences | Dan Huang Expelling Blood Stasis Capsule | Chronic Pelvic Inflammatory Disease | Model-Based Study | A: Danghuang Expelling Blood Stasis Capsule plus Antibiotics  B: Antibiotics | CEA |
| No.162: DING Yanbing, 2025 | China | Dept.of Encephalopathy, Hubei Hospital of Traditional Chinese Medicine | Duliang Soft Capsule | Migraine | Model-Based Study | A: Duliang Soft Capsule plus Conventional Standard Treatment  B: Conventional Standard Treatment | CEA |
| No.163: REN Huijun, 2024 | China | Dept.of Pharmacy,The Third People's Hospital of Chengdu | Shu Feng Jie Du Capsule (SFJDC) | Acute Exacerbation of Chronic Obstructive Pulmonary Disease | Retrospective Study | A: SFJDC plus Conventional Standard Treatment  B: Conventional Standard Treatment | CEA |
| No.164: MA Haiqing, 2021 | China | Pharmacy Department, Zhanjiang Second Traditional Chinese Medicine Hospital | Suxiaojiuxin Pill,  Compound Danshen Dripping Pill, Shexiangbaoxin Pill | Coronary Heart Disease | Retrospective Study | A: Compound Danshen Dripping Pill plus Conventional Standard Treatment  B: Suxiaojiuxin Pill plus Conventional Standard Treatment  C: Shexiangbaoxin Pill plus Conventional Standard Treatment | CEA |
| No.165: HUANG Qian, 2020 | China | Wuhan Hospital of Traditional Chinese Medicine | Tanreqing Injection | Community Acquired Pneumonia | Retrospective Study | A: Tanreqing Injection plus Levofloxacin  B: Levofloxacin | CEA |
| No.166: ZHANG Xueyi, 2021 | China | Hubei University of Chinese Medicine | Huoxuehuatanxifeng Decoction | Acute Cerebral Infarction | Retrospective Study | A: Decoction plus Conventional Standard Treatment  B: Conventional Standard Treatment | CEA |
| No.167: LI Na, 2021 | China | Pharmacy Department Lingwu County People's Hospital | Pudilan Xiaoyan Oral Liquid | Influenza | Prospective Cohort Study | A: Pudilan Xiaoyan Oral Liquid plus Oseltamivir Phosphate Granule  B: Oseltamivir Phosphate Granule | CEA |
| No.168: JIA Jianying, 2023 | China | Binzhou Hospital of Traditional Chinese Medicine | Ciwujia Injection | Insomnia | Prospective Cohort Study | A: Ciwujia Injection  B: Mirtazapine | CEA |
| No.169: LIU Yu, 2020 | China | Tianjin First Central Hospital | Shuxuening Injection, Salvianolate Injection, Yiqi Fumai Injection | Coronary Heart Disease and Angina Pectoris | Retrospective Study | A: Shuxuening Injection  B: Salvianolate Injection  C: Yiqi Fumai Injection | CEA |
| No.170: ZHU Jingyan, 2020 | China | Shanghai Baoshan Integrated Traditional Chinese and Western Medicine Hospital, Baoshan Branch of Shanghai University of Traditional Chinese Medicine Affiliated Shuguang Hospital | Xiao Xuming Decoction | Acute Ischemic Stroke | Retrospective Study | A: Xiao Xuming Decoction  B: Placebo | CEA |
| No.171: HUANG Haitao, 2022 | China | State Key Laboratory for New Drug Development of Tibetan Medicine | Ruyi Zhenbao Pill, Synovitis Granule | Osteoarthritis | Retrospective Study | A: Ruyi Zhenbao Pill  B: Synovitis Granule | CEA |
| No.172: WANG Xingdong, 2021 | China | Dept.of Pharmacy, General Hospital of Eastern Theater Command of PLA | Sofren Injection, Danhong Injection | Stable  Angina Pectoris of Coronary Heart Disease | Retrospective Study | A: Sofren Injection  B: Danhong Injection | CMA |
| No.173: SONG Miaoyuan, 2022 | China | The Fifth Hospital of Harbin Nephrology dept | Compound Qinlan Oral Liquid | Chronic Bronchitis | Prospective Cohort Study | A: Compound Qinlan Oral Liquid  B: Azithromycin Tablet | CEA |
| No.174: WANG Xiu, 2023 | China | Taian First People's Hospital Taian | Ciwujia Injection | Angina Pectoria of Coronary Heart Disease | Prospective Cohort Study | A: Ciwujia Injection plus Conventional Standard Treatment  B: Conventional Standard Treatment | CEA |
| No.175: PAN Zhehao, 2023 | China | Deqing County People's Hospital/Deqing Branch of Sir Run Run Shaw Hospital Affiliated to Zhejiang University | Yunnan Baiyao Plaster, Futalin Ointment, Qizhengzhitong Plaster | Orthopedic Diseases | Retrospective Study | A: Chinese Medicinal Plaster  B: Conventional Standard Treatment | CEA |
| No.176: Feng Rongwei, 2020 | China | The 980th Hospital of Joint Logistics Support Force of Chinese People's Liberation Army | Xueshuantong Injection, Dengzhanxixin Injection, Danhong Injection, Shuxuetong Injection, Ginkgolides Injection | Acute Cerebral Infarction | Retrospective Study | A: Xueshuantong Injection plus Conventional Standard Treatment  B: Dengzhanxixin Injection plus Conventional Standard Treatment  C: Danhong Injection plus Conventional Standard Treatment  D: Shuxuetong Injection plus Conventional Standard Treatment  E: Ginkgolides Injection plus Conventional Standard Treatment | CEA |
| No.177: LI Meifang, 2024 | China | First Teaching Hospital of Tianjin University of Traditional Chinese Medicine, National Clinical Research Center for Chinese  Medicine Acupuncture and Moxibustion | Fuganlin Oral Liquid, Xiaoer Jiebiao Oral Liquid | Acute Upper Respiratory Tract Infection (Qi Deficiency Wind Heat Syndrome) | Retrospective Study | A: Fuganlin Oral Liquid plus Xiaoer Jiebiao Oral Liquid Simulator  B: Xiaoer Jiebiao Oral Liquid plus Fuganlin Oral Liquid Simulator | CEA |
| No.178: YANG Qian, 2024 | China | School of Management, Beijing University of Chinese Medicine | Puyuan Hewei Capsule | Functional Dyspepsia | Retrospective Study | A: Puyuan Hewei Capsule plus Domperidone vs. Domperidone  B: Puyuan Hewei Capsule plus Itopride Hydrochloride Tablet vs. Itopride Hydrochloride Tablet | CEA |
| No.179: LU Yunfei, 2021 | China | Dept.of Hepatology, Shanghai Shuguang Hospital Affiliated to Shanghai University of Traditional Chinese Medicine | Ku Huang Granule, Compound Glycyrrhizin Capsule | Viral Hepatitis | Retrospective Study | A: Ku Huang Granule  B: Compound Glycyrrhizin Capsule | CEA |
| No.180: CHEN Yi-peng ,et al., 2021 | China | Tongdao Hemodialysis Center | Yunnan Baiyao Capsule | Perioperative Bleeding During Posterior Cervical Laminoplasty | Prospective RCT | A: Yunnan Baiyao Capsule  B: Placebo | CEA |
| No.181: LIU Zhihui, 2022 | China | DepartmentⅡof Neurology, the Second Hospital of Heilongjiang Province | Xueshuantong Capsule | Cerebrovascular Accident | Prospective Cohort Study | A:Xueshuantong Capsule plus Conventional Standard Treatment  B: Clopidogrel plus Aspirin plus Conventional Standard Treatment | CMA |
| No.182: XU Lingjun, 2022 | China | Dept.of Emergency, the Children's Hospital of Jiangxi Province | Carmine Leaf Granule | Acute Upper Respiratory Tract Infection | Prospective Cohort Study | A: Carmine Leaf Granule  B: No-treatment Control | CEA |
| No.183: HUA Xiangxiang, 2021 | China | Dept.of pharmacy, Rudong people's Hospital | Pudilan Xiaoyan Oral Liquid | Influenza | Prospective Cohort Study | A: Oseltamivir Phosphate Granule plus Pudilan Xiaoyan Oral Liquid  B: Oseltamivir Phosphate Granule | CEA |
| No.184: LIU Huan, 2020 | China | Institute of Basic Research in Clinical Medicine,China Academy of Chinese Medical Sciences | Jinye Baidu Granule  Compound Shuanghua Capsule | Upper Respiratory Tract Infection | Model-Based Study | A: Jinye Baidu Granule plus Compound Shuanghua Capsule Simulator  B: Compound Shuanghua Capsule plus Jinye Baidu Granule Simulator | CEA |
| No.185: CHEN Wenwen, 2024 | China | Jinhe Tibetan Medicine Co., LTD. | Anerning Granule, Xiaoer Cold Granule | Upper Respiratory Tract Infection | Model-Based Study | A: Anerning Granule  B: Xiaoer Cold Granule | CEA |
| No.186: WANG Zhijun, 2024 | China | Dept.of Cardiology, Affiliated Hospital of North China University of Science and Technology | Shexiang Baoxin Pill, Tongxinluo Capsule | Coronary Heart Disease | Model-Based Study | A: Shexiang Baoxin Pill  B: Tongxinluo Capsule | CEA |
| No.187: QU Detao, 2023 | China | Dept.of Neurology,the People's Hospital of Guanghan City | Anisodine Hydrobromide Injection | Moderate and  Severe Acute Ischemic Stroke | Prospective RCT | A: Anisodine Hydrobromide Injection  B: Butyphthalide Sodium Chloride Injection | CEA |
| No.188: XIONG Liping, 2020 | China | Shenyang Hunnan District Central Hospital Liaoning | Ginko Leaf Extract Injection, Danshen Chuanxiongqin Injection | Acute Ischemic Stroke | Retrospective Study | A: Ginko Leaf Extract Injection  B: Danshen Chuanxiongqin Injection | CEA |
| No.189: ZHANG Yaxuan, 2022 | China | School of Public Health, Fudan University | Xiaoerguangpuzhixie Oral Liquid | Infantile Diarrhea | Retrospective Study | A: Xiaoerguangpuzhixie Oral Liquid plus Conventional Standard Treatment vs. Conventional Standard Treatment  B: Xiaoerguangpuzhixie Oral Liquid plus Conventional Standard Treatment and Montmorillonite Powder vs. Conventional Standard Treatment and Montmorillonite Powder | CEA |
| No.190: WANG Qingqing, 2025 | China | Eye Hospital, China Academy of Chinese Medical Sciences | Qiangli Dingxuan Tablet | Vertigo and Hypertension | Retrospective Study | A: Qiangli Dingxuan Tablet  B: Conventional Standard Treatment | CEA |
| No.191: XU Huanxiang, et al., 2020 | China | Nanjing University of Chinese Medicine | Pingwei San  Fengreganmao Fang | Influenza | Retrospective Study | A: Pingwei San  B: Fengreganmao Fang | CEA |
| No.192: WANG FENG, et al.2021 | China | Hebi Traditional Chinese Medicine Hospital | San Diao Tang | Insomnia Disorder | Retrospective Study | A: San Diao Tang  B: Conventional Standard Treatment | CEA |
| No.193: Zuo Kaini, 2020 | China | School of Management,Beijing University of Chinese Medicine | Liuwei Dihuang Pill, Xiaoke Pill | Type 2 Diabetes Mellitus | Retrospective Study | A: Liuwei Dihuang Pill plus Metformin vs. Metformin  B: Liuwei Dihuang Pill plus Xiaoke Pill vs. Xiaoke Pill | CEA |
| No.194: ZHANG Yaxuan, 2022 | China | School of Public Health, Fudan University | Xiaozheng Pill, Rupixiao Capsule, Rujiekang Pill | Breast Hyperplasia | Retrospective Study | A: Xiaozheng Pill vs. Rupixiao Capsule  B: Xiaozheng Pill vs. Placebo  C: Xiaozheng Pill vs. Rujiekang Pill | CEA |
| No.195: TAN Shurao, 2021 | China | Wuyi Hospital of Traditional Chinese Medicine, Jiangmen City, Guangdong Province Jiangmen | Jiawei Yiqibushen Decoction | Chronic Glomerulonephritis | Retrospective Study | A: Jiawei Yiqibushen Decoction plus Conventional Standard Treatment  B: Conventional Standard Treatment | CEA |
| No.196: HUANG Shaohua, 2025 | China | Xinchang County Hospital of Traditional Chinese Medicine | Not Specified | Neurological Disorders | Retrospective Study | A: TCM plus Conventional Standard Treatment  B: Conventional Standard Treatment | CEA |
| No.197: ZHANG He, 2025 | China | Tongzhou District Hospital of Integrated Traditional Chinese and Western Medicine | Ertongqingfen Oral Liquid | Pediatric Cough with Yin Deficiency and Lung Heat Pattern | Prospective Cohort Study | A: Ertongqingfen Oral Liquid  B: Montelukast Sodium | CEA |
| No.198: LV Xiaoqin, 2020 | China | Taixing Center for Disease Control and Prevention Jiangsu Province | Baixuanxiatare Tablet, Fufangzhenzhuanchuang Tablet, Xiaocuo Pill | Acne Vulgaris | Retrospective Study | A: Baixuanxiatare Tablets  B: Fufangzhenzhuanchuang Tablet  C: Xiaocuo Pill | CEA |

CEA, cost-effectiveness analyses. CMA, cost-minimization analyses. CUA, cost-utility analyses. CBA, cost-benefit analyses.

**Table S2. Main study characteristics of PE**

| **Study ID** | **Perspective** | **Model** | **Model States** | **Horizon** | **Sample** | **Cost Scope** | **Cost Items** | **Health Outcome** |
| --- | --- | --- | --- | --- | --- | --- | --- | --- |
| No.1: LongChen, Xu, 2023 | Payer | Markov | CHB, HBeAg Seroconversion (HBeAg-positive patients only), Compensated cirrhosis, Decompensated Cirrhosis, Hepatocellular carcinoma, Death | 30y (Lifetime) | 394 | Direct Medical Cost, Indirect Cost, Intangible Cost | / | Utility Outcome: QALY  Effectiveness Outcome: Primary Endpoint: Resolution of liver fibrosis and reduction in liver stiffness  Secondary Endpoints: Histological improvement, Non-Invasive fibrosis index |
| No.2: Cong Wang, 2025 | Health Care Provider's | Markov | No Fracture, Simple fracture, Complex fracture, Bedridden due to hip fracture, Death from fracture or other causes | 30y (Lifetime) | / | Direct Medical Cost | Drug fee, Treatment fee | Utility Outcome: QALY |
| No.3: Yumei He, 2023 | Healthcare System | Markov | CKD Stage 3, CKD Stage 4, CKD Stage 5 (Non-Hemodialysis), CKD Stage 5 (Hemodialysis), Death. | 20y (Lifetime) | 3,444 | Direct Medical Cost | Drug fee | Utility Outcome: QALY  Effectiveness Outcome: Serum creatinine, Adverse events |
| No.4: Lu Wang, 2025 | Payer | Markov | NYHA I, II, III, IV, and Death | 19y (Lifetime) | 3,110 | Direct Medical Cost |  | Utility Outcome: QALY |
| No.5: Liang Lu, MM, 2022 | Patient's | Decision Tree | Effective, Ineffective | >12m | 4,290 | Direct Medical Cost | Drug fee, Materials fee, Intravenous Injection fee, Examination fee, Medical Services fee, Hospitalization fee | Effectiveness Outcome: Judgment criteria for angina pectoris, ECG effective rate |
| No.6: Yang shuo, 2024 | Healthcare System | / | / | 1-2w | 457 | Direct Medical Cost | Registration fee, Drug fee, Surgical fee, Consultation fee, Treatment fee, Nursing fee, Monitoring fee, Material fee, Room fee, Laboratory Test fee | Effectiveness Outcome: Total effective rate |
| No.7: Guoqiang Liu, 2024 | Healthcare System | Markov | STEMI without  New events, Stroke, Post-stroke, Recurrent myocardial infarction,  Post-recurrent Myocardial infarction, Death | CEA: 30d, 1y  CUA: 1y, 16y | 3,777 | Direct Medical Cost | Bed fee, Nursing fee, Medication fee, Laboratory test and Examination fee, Diagnosis and Treatment fee, Surgical fee | Utility Outcome: QALY  Effective Outcome: MACCE |
| No.8: YANG Xuanru, 2024 | Societal | Decision Tree | / | 2w | 476 | Direct Medical Cost | Drug fee | Effectiveness Outcome: TCM Syndrome Score |
| No.9: Junjie Zhu, 2024 | Healthcare System | Markov | PD, PF, Death | 10y | / | Direct Medical Cost | Drug fee, Follow-up fee, Disease Management fee, Subsequent Treatment fee, End Life fee | Utility Outcome: QALY |
| No.10: ZHAO Xiaoxiao, 2023 | Healthcare System | Decision Tree | / | 25d | 2,503 | Direct Medical Cost | Drug fee, Treatment fee | Effectiveness Outcome: NIHSS Score |
| No.11: Jie Pan, 2024 | / | Markov | CVD event-free, Non-fatal MI, Post-MI, Non-fatal Stroke, Post-stroke, Vascular death (MI Or Stroke), Non-vascular death | 30y | 2,674 | Direct Medical Cost | Drug fee | Utility Outcome: QALY |
| No.12: Yake Lou, et al. 2025 | Healthcare System | Markov | PD, PF, Death | 10y | / | Direct Medical Cost | Drug fee, Follow-up fee, Disease management fee, Subsequent treatment fee, End life fee | Utility Outcome: QALY |
| No.13: WU Xue, 2024 | Healthcare System | / | / | 56d | 3,168 | Direct Medical Cost | Drug fee | Effectiveness Outcome: Total effective rate |
| No.14: Yang Li, 2021 | Patient's | Decision Tree | Mild, Moderate and  Severe | >12m | 1,425 | Direct Medical Cost | Drug fee | Effectiveness Outcome: Total effective rate |
| No.15: Zhou Wenxin, 2023 | Societal | Decision Tree+Markov | CKD 1-5, Death | 20y | 479 | Direct Medical Cost | Drug fee, Examination fee | Utility Outcome: QALY |
| No.16: CHEN Zijia, 2024 | Healthcare System | Decision Tree | Effective, Ineffective | 2w | 176 | Direct Medical Cost | Drug fee, Treatment fee | Effectiveness Outcome: Total effective rate |
| No.17: CHENG lili, 2022 | Societal | Markov | Effective, Ineffective | 30d | / | Direct Medical Cost | Drug fee | Utility Outcome: QALY  Effectiveness Outcome: Four-week effective rate |
| No.18: PAN Huimin, 2024 | Healthcare System | Decision Tree | NYHAⅠ~Ⅳ | 90d | / | Direct Medical Cost | Drug fee | Utility Outcome: QALY |
| No.19: Kai Xu, 2025 | Healthcare System | Decision Tree | CP, PR, PD, SD | 3w | / | Direct Medical Cost | Drug fee, Examination fee, Treatment fee for AE | Effectiveness Outcome: ORR |
| No.20: LIANG Liang, 2024 | Payer | Markov | HBeAg Seroconversion (Transition from HBeAg positive to HBeAg negative), Chronic hepatitis B, Compensated cirrhosis, Decompensated cirrhosis, Liver cancer, Death | 30y | / | Direct Medical Cost | Drug fee, Treatment fee | Utility Outcome: QALY |
| No.21: WAN Feng, 2023 | Payer | / | / | 42d | 2,270 | Direct Medical Cost | Registration fee, Consultation fee, Drug fee, Examination fee, Hospitalization fee, Surgical fee, Nursing fee, Monitoring fee, Material fee | Utility Outcome: QALY  Effectiveness Outcome: Postpartum hemorrhage rate, Heights of fundus of uterus after postpartum 72H, Incidence of subinvolution of uterus and lochia duration |
| No.22: ZHANG XinYing, 2024 | Societal | / | / | 5d | 108 | Direct Medical Cost, Indirect Cost | Drug fee, Productivity Loss fee | Effectiveness Outcome: Time of double CT, Conversion to negative after admission, Total score of TCM syndrome, Score of TCM main syndrome and score of TCM secondary syndrome |
| No.23: LIANG wanxian, 2022 | Healthcare System | / | Achieved ORR, Did not achieve ORR | 60d | 3,044 | Direct Medical Cost | Drug fee, Hospitalization fee, Surgical fee, Registration fee, Treatment fee | Effectiveness Outcome: ORR, CRR |
| No.24: ZHAO Yifan, 2022 | Healthcare System | Decision Tree | CR, PR, SD, PD | 42d | / | Direct Medical Cost | Drug fee, Adverse Reaction Treatment fee, Diagnostic Testing fee, Hospitalization fee | Effectiveness Outcome: ORR |
| No.25: Yuliang Xiang, 2021 | Societal | Markov | No significant Disability, Disability, Recurrence, Death | 13y | 949 | Direct Medical Cost, Direct Non-Medical Cost | Drug fee, Treatment fee, Examination fee, Hospital Bed fee, Transportation fee | Utility Outcome: QALY |
| No.26: Lisong Yang, et al. 2020 | Payer | / | / | / | 2,473 | Direct Medical Cost | Drug fee, Hospitalization fee | Effectiveness Outcome: Hospitalization duration, 10d re-hospitalization rate, 30d re-hospitalization rate, 90d re-hospitalization rate |
| No.27: Meng-Bin Tang, 2024 | Payer | / | / | 5y | 13,848 | / | / | Effectiveness Outcome: Mortality rates (All-cause mortality, lung cancer-specific mortality), differences in mortality rates among patients with different cancer stages |
| No.28: Gordon Liu, 2021 | / | / | / | / | 1,903 | Direct Medical Cost, Direct Non-Medical Cost | Inpatient fee, Outpatient fee, Drug fee | Utility Outcome: QALY  Effectiveness Outcome: Glycated hemoglobin (HbA1C), Adverse events |
| No.29: ZHAN Xudan, 2024 | Societal | / | / | / | 200 | Direct Medical Cost | Drug fee, Treatment fee, Bed fee, Nursing fee, Decoction Preparation fee | Effectiveness Outcome: Total effective rate, Cardiac function (LVEF, LVEDD, LVESD, 6MWD) |
| No.30: CAO ShiHuan, 2024 | Healthcare System | Decision Tree | / | / | 1,735 | Direct Medical Cost | Drug fee | Effectiveness Outcome: Total effective rate, cough disappearance rate,nucleic acid negative conversion rate, CT improvement rate |
| No.31: Wei Zhongyi, 2024 | Healthcare System | Decision Tree | / | 5d | 623 | Direct Medical Cost, Indirect Cost | / | Effectiveness Outcome: TCM Syndromes effective rate |
| No.32: DAI Yingying, 2023 | Societal | Markov | mRS score 0-2 (mild disability), mRS score 3-5 (moderate disability), mRS score 6 (death), recurrence | 13y | / | Direct Medical Cost | Medication fee, Examination fee, Laboratory test fee, Bed fee, Nursing fee, Other related treatment fee | Utility Outcome: QALY |
| No.33: YANG Shuo, 2023 | Healthcare System | Decision Tree | / | 66d | / | Direct Medical Cost | Drug fee, Examination fee | Effective Outcome: IPSS |
| No.34: Ming Hu, 2022 | Healthcare System | Markov | Stable phase, Non-severe Exacerbations, Severe exacerbations, Death | 10y |  | Direct Medical Cost | Drug fee, Treatment fee, Examination fee | Utility Outcome: QALY |
| No.35: Qian Xu et al., 2020 | Healthcare System | Markov | Alive Without Stroke/MI, MI, Stroke, Post-stroke, Post-MI, Death | 20y | 108 | Direct Medical Cost | Drug fee, Examination fee, Consultation fee | Utility Outcome: QALY  Effectiveness Outcome: Nocturnal Blood Pressure |
| No.36: SUN Yang, 2024 | Healthcare System | Markov | H, NH, MI, Stroke, Death | 30y | / | Direct Medical Cost | Drug fee, Medical Service fee, Nursing fee, Diagnostic fee, Surgical fee, Material fee | Utility Outcome: QALY |
| No.37: LI Mengyuan, 2025 | Healthcare System | Decision Tree+Markov | mRS 0-2, mRS 3-5, mRS6, Recurrent | 40y | 457 | Direct Medical Cost | Drug fee, Hospitalization fee, Prognosis fee, Treating Relapses fee | Utility Outcome: QALY |
| No.38:XIAO GuiRong, 2021 | Societal | / | / | 5d | 220 | Direct Medical Costs+Direct Non-Medical Costs+Indirect Costs | Drug Fee, Medical Service Fee, Examination Fee, Nursing Fee, Treatment Fee, Adverse Reactions Treatment Fee, Material Fee, Transportation Fee, Food and Accommodation Fee, Nutritional Food Fee, Lost Wages | Effectiveness Outcomes:  The Clinical Symptoms of Relief Time; Complete Antipyretic Time; effective rate of TCM Syndrome |
| No.39: WEI Chunyan, 2022 | Healthcare System | / | / | 1y | 1,688 | Direct Medical Cost | Drug fee, Outpatient fee, Examination fee | Effectiveness Outcome: The clinical effective rate, serum creatinine, urea nitrogen |
| No.40: CAO ShiHuan, 2023 | Healthcare System | Decision Tree | / | 7d | 185 | Direct Medical Cost | Registration fee, Drug fee, Laboratory fee, Treatment fee, Adverse Reaction Treatment fee | Effectiveness Outcome: Total effective rate |
| No.41: LU Zhenkai, 2024 | Healthcare System | Markov | / | 12w | 1,908 | Direct Medical Cost | / | Effectiveness Outcome: Triglyceride levels, Lipid profile effective rate |
| No.42: LIU Teng, 2021 | Societal, Healthcare System | Decision Tree | Cured, Significantly Improved, Effective, Ineffective | 1y | 416 | Direct Medical Cost , Indirect Cost | Drug fee, Lost Wages | Utility Outcome: QALY |
| No.43: CUI Mancang, 2020 | / | Decision Tree | Cured, Significantly Improved, Effective,  Ineffective | 15d | 279 | Direct Medical Cost | Drug fee, Examination fee, Nursing fee, Treatment fee, Bed fee, Laboratory Test fee, Material fee | Effectiveness Outcome: Total effective rate |
| No.44: LIANG WanXian, 2024 | Healthcare System | Decision Tree | Effective, Ineffective | 5d | 2,323 | Direct Medical Cost | Drug fee | Effectiveness Outcome: Total effective rate |
| No.45: Pengli Su, 2024 | Societal | Markov | No Angina, Monthly Angina, Daily Angina, Death | 1y | 872 | Direct Medical Cost, Direct Non-Medical Cost, Indirect Cost | Drug fee, Examination fee, Treatment fee, Adverse Reaction Treatment fee, Hospitalization fee, Transportation fee, Lost Wages | Utility Outcome: QALY |
| No.46: CHEN Zijia, 2024 | Healthcare System | Decision Tree | Effective, Ineffective | 7d | 146 | Direct Medical Cost | Drug fee, Examination fee, Treatment fee, Registration fee | Effectiveness Outcome: Disappearance rate of gingival pain |
| No.47: LIU Huan, 2022 | Healthcare System | Decision Tree | / | 53d | / | Direct Medical Cost | Drug fee, Treatment fee | Effectiveness Outcome: Lund-Mackay score |
| No.48: WANG Zhi-heng, 2021 | Healthcare System | Markov | Acute exacerbation requiring hospitalization, Stable period without hospitalization, Death | 3y | / | Direct Medical Cost | Drug fee | Utility Outcome: QALY |
| No.49: GUO ZhaoTing, 2021 | Societal | Markov | Independent living/No significant disability, Stroke disability, Death, recurrent stroke | 8y | 324 | Direct Medical Cost, Direct Non-Medical Cost | Drug fee, Diagnostic fee, Bed fee, Treatment fee, Attendant fee, Transportation fee | Utility Outcome: QALY  Effectiveness Outcome: Recurrent cerebral infarction at 28 Days, NIHSS score, discharge MRS score |
| No.50: Hu Haiyao, 2022 | Healthcare System | / | / | 3d | 114 | Direct Medical Cost | Drug fee, Outpatient fee, Examination fee, Adverse Reaction Management fee | Effectiveness Outcome: Total effective rate, Clinical symptom improvement (time to fever resolution, time to cough resolution, time to sore throat resolution, time to nasal discharge resolution), Inflammatory cytokine levels (Hs-CRP, TNF-α, IL-6, IL-4) |
| No.51: LI Han, 2020 | / | / | / | <14d | 92 | Direct Medical Cost | Drug fee, Consultation fee, Treatment fee | Effectiveness Outcome: Total effective rate |
| No.52: SHI Suisui, 2023 | / | / | / | 6m | 86 | Direct Medical Cost | Drug fee | Effectiveness Outcome: Total effective rate |
| No.53: WANG Zhuojue, 2022 | Health Care Provider's | / | / | 21d | 718 | Direct Medical Cost | Examination fee, Treatment fee, Drug fee, Hospitalization fee | Effectiveness Outcome: Total effective rate |
| No.54: ZHOU Li, 2020 | Societal | / | / | 14d | 162 | Direct Medical Cost, Direct Non-Medical Cost | Drug fee, Transportation fee, Meal fee, Caregiver fee | Effectiveness Outcome: Primary Efficacy Endpoint: Recurrence rate, Mortality rate at Day 28 Post-enrollment  Secondary Efficacy Endpoints: NIHSS score, MRS, BI score |
| No.55: Xiang Gao, 2024 | / | / | / | / | 161 | Direct Medical Cost, Indirect Cost | Hospitalization fee, Drug fee, Time Lost by Patients | Effectiveness Outcome: SFR |
| No.56: Sun Yan, 2025 | / | / | / | 24w | 200 | Direct Medical Cost | Drug fee | Effectiveness Outcome: Total effective rate |
| No.57: XIA Ruyu, 2022 | Health Care Provider's | / | / | 14d | 7,680 | Direct Medical Cost | Outpatient fee, Registration fee, Drug fee, Examination fee, Treatment fee | Effectiveness Outcome: HP eradication rate |
| No.58: LEI Chao, 2022 | Healthcare System | Decision Tree | / | 60d | 81 | Direct Medical Cost | Drug fee, Medical fee | Effectiveness Outcome: Number of epilepsy |
| No.59: HU Xingmao, 2020 | Healthcare System | Decision Tree | Effective, Ineffective | 2w | 167 | Direct Medical Cost, Direct Non-Medical Cost | Laboratory Tests fee, Examinations fee, Treatments fee, Hospitalization fee, Drug fee, other fee, Adverse Reaction Treatment fee, Accommodation And Meals fee, Transportation fee, Attendant Care fee | Effectiveness Outcome: Total effective rate |
| No.60: SHENG Ye, 2021 | Healthcare System | Markov | No Angina Pectoris, Angina Pectoris Monthly, Angina Pectoris Weekly, Angina Pectoris Daily | CEA: 4w  CUA: 1y | 2,374 | Direct Medical Cost, Direct Non-Medical Cost, Indirect Cost | Drug fee, Transportation fee, Lost Wages, Attendant care fee | Utility Outcome: QALY  Effectiveness Outcome: Effective rate of angina pectoris, ECG effective rate, Nitroglycerin arrest rate, Effective rate of TCM syndromes |
| No.61: ZHANG Lidan, 2022 | Societal | Decision Tree | / | 6m | 2,217 | Direct Medical Cost | Drug fee, Treatment fee | Effective Outcome: Median nerve conduction velocity |
| No.62: ZHENG Yingying, 2021 | Healthcare System | Decision Tree | Effective, Ineffective | 56d | / | Direct Medical Cost | Drug fee | Effective Outcome: Effective rates of symptom improvement, ECG effective rates |
| No.63: ZHANG Shiqin, 2025 | Healthcare System | Decision Tree | Effective, Ineffective | 4w | / | Direct Medical Cost | Drug fee | Effectiveness Outcome: Total effective rate, NIHSS neurological function evaluation, ADL Score, Total TCM syndrome volume |
| No.64: LI Wei, 2021 | Societal | Markov | Healthy, mRS 0-2 (Recurrent Stroke Independent), mRS 3-5 (Recurrent Stroke-dependent), Death | 5y | / | Direct Medical Cost, Indirect Cost | Drug fee, Treatment fee, Lost Wages | Utility Outcome: QALY |
| No.65: WANG Zhiheng, 2021 | Payer | Markov | mRS 0-2, mRS 3-5, Recurrence, Death | 30y | 439 | Direct Medical Cost | Drug fee, Hospitalization fee, Prognostic medical fee (Outpatient and inpatient rehabilitation, secondary prevention medication fee), Relapse treatment fee | Utility Outcome: QALY |
| No.66: KOU Zhenzhen, 2023 | / | / | / | 28d | 120 | Direct Medical Cost | Drug fee, Nursing fee, Treatment fee | Effectiveness Outcome: Clinical effective rate, Myocardial enzyme spectrum, CTnI, adverse events |
| No.67: GUO Xiaojie, 2020 | Healthcare System | / | / | 90d | 80 | Direct Medical Cost | Drug fee, Examination fee, Treatment fee | Effectiveness Outcome: Total effective rate |
| No.68: ZHEN Hui, 2021 | Societal | / | / | 14d | 400 | Direct Medical Cost, Direct Non-Medical Cost, Indirect Cost | Registration fee, Consultation fee, Drug fee, Adverse Reactions treatment fee, Accommodation And Meal fee, Transportation fee, Nutritional fee, Wage Lost Wage | Utility Outcome: QALY  Effectiveness Outcome: Total effective rate |
| No.69: LI Xuan, 2022 | Societal | / | / | 7d | 468 | Direct Medical Cost, Direct Non-Medical Cost | Registration fee, Examination fee, Drug fee, Adverse Reactions Treatment fee, Transportation fee | Effectiveness Outcome:  Cure Rates of TCM Syndromes, Complete, Disappearance Rates, Disappearance Rates of Constipation |
| No.70: GAN Xiong, 2023 | / | / | / | 12w | 512 | Direct Medical Cost, Direct Non-Medical Cost, Indirect Cost | Registration fee, Treatment fee, Examination fee, Bed fee, Drug fee, Nursing fee, Caregiver fee, Nutritional fee, Transportation fee, Accommodation fee | Effective Outcome: Median reduction rate of NT-proBNP, Incidence of CCEs, Total effective rate of cardiac function classification, Change of LVEF of the heart, Total score improvement value of the MLHFQ, 6MWD |
| No.71: LIU Zhaohui, 2024 | Societal | / | / | 4m | 236 | Direct Medical Cost | Drug fee, Registration fee, Examination fee, Adverse Reactions Treatment fee | Utility Outcome: QALY  Effective Outcome:  VAS for abdominal pain, Recovery rate of TCM syndromes, Recovery rate of diseases, Recurrence rate of pelvic pain, Incidence rate of pelvic inflammatory diseases |
| No.72: CUI Xin, 2022 | Healthcare System | Decision Tree | Effective, Ineffective | 12w | 74 | Direct Medical Cost | Registration fee, Drug fee, Nursing fee, Treatment fee, Surgery fee, Consultation fee, Monitoring fee, Material fee, Ward fee, Laboratory test fee, Oxygen fee, Other medical fee | Effective Outcome: Clinical effective rate, NIHSS |
| No.73: WEI Ruili, 2020 | Societal | Decision Tree | / | 4w | 2,608 | Direct Medical Cost | Drug fee, Bed fee, Nursing fee, Medical Service fee | Effectiveness Outcome:  Decreased levels of CK-MB |
| No.74: CUI Xin, 2023 | Healthcare System | Decision Tree | Effective, Ineffective | 24w | / | Direct Medical Cost | Drug fee | Effectiveness Outcome: Effective rate of TCM syndromes |
| No.75: CUI Xin, 2023 | Healthcare System | Decision Tree | / | 6m | / | Direct Medical Cost | Drug fee | Effectiveness Outcome:  VAS score |
| No.76: LIU Jifang, 2023 | Healthcare System | Decision Tree | Effective, Ineffective | 4w | 133 | Direct Medical Cost | Drug fee, Adverse Reactions treatment fee, Examination fee, Laboratory tests fee | Effective Outcome: PSQI |
| No.77: WANG Fangxu, 2023 | Healthcare System | Decision Tree | Effective, Ineffective | 30d | 1,164 | Direct Medical Cost | Drug fee, Adverse Reactions Treatment fee | Effectiveness Outcome: Clinical effective rate |
| No.78: PAN Jie, 2021 | Payer | Markov | Baseline characteristics, New-onset myocardial infarction, Prognosis of myocardial infarction, New-onset stroke, Prognosis of stroke, Death | 30y | / | Direct Medical Cost | Drug fee, Hospitalization fee, Outpatient fee, Rehabilitation fee, Secondary Prevention fee | Utility Outcome: QALY |
| No.79: CUI Xin, 2023 | Healthcare System | Decision Tree | Effective, Ineffective | 7d | 60 | Direct Medical Cost | Drug fee, Treatment fee, Examination fee | Effectiveness Outcome: Effective rate of TCM syndromes |
| No.80: XU Haiting, 2024 | Healthcare System | Markov+Decision Tree | mRS 0-2, mRS 3-5, mRS 6, Death | 30y | 3,072 | Direct Medical Cost | Drug fee, Hospitalization fee, Nursing fee | Utility Outcome: QALY |
| No.81: LI Fan, 2020 | / | / | / | 30d | 64 | Direct Medical Cost | Drug fee, Consultation fee, Examination fee, Nursing fee, Bed fee | Effectiveness Outcome: Total effective rate |
| No.82: LIU Xing, 2020 | / | / | / | / | 400 | Direct Medical Cost | Drug fee, Hospitalization fee, Laboratory Test fee, Nursing fee, Material fee | Effectiveness Outcome: Total effective rate (Absolute Values of CD3+, CD4+, CD8+ In Peripheral Blood T-Lymphocyte Subsets, Negative conversion rate of sputum bacteria, Effective rate of focus absorption, Cavity closure rate, TCM symptoms improvement rate, Adverse events) |
| No.83: Ma Luyao, 2023 | / | / | / | / | 1,925 | Direct Medical Cost | Drug fee | Effectiveness Outcome: Total effective rate |
| No.84: LIU Dan, 2022 | Healthcare System | / | / | 5d | 1,036 | Direct Medical Cost | Drug fee, Examination fee, Laboratory Test fee, Registration fee, Treatment fee | Effectiveness Outcome: Time to complete fever resolution, Time to symptom relief, Hospitalization rate. |
| No.85: WANG Yaqin, 2021 | Healthcare System | / | / | 16w | 868 | Direct Medical Cost | Drug fee, Adverse reactions treatment fee | Effectiveness Outcome: Clinical effective rate |
| No.86: XU Longchen, 2022 | / | / | / | 14d | 1,950 | Direct Medical Cost | Drug fee | Effectiveness Outcome: Total effective rate  Benefit Outcome: Savings in lost work time Cost, Savings in hospitalization Cost |
| No.87: MA Luyao, 2022 | / | / | / | 56d | 1,507 | Direct Medical Cost | Drug fee | Effectiveness Outcome: Total effective rate, ECG effective rate |
| No.88: Lian Tang, 2022 | Healthcare System | / | / | 2w | 2,818 | Direct Medical Cost | Drug fee, Examination fee, Bed fee, Nursing fee | Effectiveness Outcome: Clinical effective rate, ECG effective rate, Angina pectories effective rate |
| No.89: ZHANG XinYing, 2024 | Societal | Decision Tree | Hospitalization, Discharge after 6 weeks/42 days, 42-120 days after discharge, 120 days to 1 year after discharge/treatment. | 1y | 195 | Direct Medical Cost | Drug fee, Hospitalization fee | Utility Outcome: QALY |
| No.90: WANG Xiaoli, 2023 | Healthcare System | Decision Tree | Stable, Hospitalization of unstable angina | 6m | 292 | Direct Medical Cost | Drug fee, Examination fee, Laboratory test fee | Utility Outcome: QALY |
| No.91: ZHU Longxun, 2024 | Healthcare System | Decision Tree | Effective, Ineffective | / | / | Direct Medical Cost | Drug fee | Effectiveness Outcome: Total effective rate |
| No.92: HOU Xiaohua, 2024 | Healthcare System | Decision Tree | Symptom Relief, Mild, Moderate, Severe | 5y | 342 | Direct Medical Cost | Drug fee | Utility Outcome: QALY |
| No.93: LI Junhui, 2025 | Healthcare System | Decision Tree | / | 90d | 432 | Direct Medical Cost | Drug fee, Examination fee, Adverse Reactions Treatment fee | Utility Outcome: QALY |
| No.94: LIU Chang, 2022 | Societal | Markov | DKD1～5, Death | 1y | 439 | Direct Medical Cost, Indirect Cost | Drug fee, Treatment fee, Lost wages | Utility Outcome: QALY |
| No.95: LI Menglan, 2020 | / | / | / | 10d | 123 | Direct Medical Cost | Drug fee | Effectiveness Outcome: Total effective rate |
| No.96: ZHANG Huiling, 2020 | Societal | / | / | 3m | 40 | Direct Medical Cost, Indirect Cost | Direct Medical fee: Drug fee, Diagnostic fee, Consultation fee, Treatment fee,  Lost Wages | Effectiveness Outcome: Total effective rate |
| No.97: Yang Guangyao, 2024 | / | / | / | 2w | 42 | Direct Medical Cost | Drug fee, Bed fee, Treatment fee, Nursing fee, Consumables fee | Effectiveness Outcome: Primary Outcome: Pathogenic microorganism culture, MIC value, Infection control rate  Secondary Outcome: Wound surface area and wound area healing rate |
| No.98: JIANG Qiyun, 2025 | Healthcare System | Markov | ACR <20%, 20%≦ ACR <50%, 50%≦ ACR <70%, ACR≧70% | 6m | / | Direct Medical Cost | Drug fee, Examination fee, Hospitalization fee | Utility Outcome: QALY |
| No.99: LI Xuejing, 2021 | Healthcare System | / | / | 42d | / | Direct Medical Cost | Drug fee | Effectiveness Outcome: Total effective rate |
| No.100: WEI Jiayin, 2023 | / | / | / | 3m | / | Direct Medical Cost | Drug fee | Effectiveness Outcome: Total effective rate |
| No.101: HE Yumei, 2024 | Healthcare System | Decision Tree | Effective, Ineffective | 3m | / | Direct Medical Cost | Drug fee | Effectiveness Outcome: Total effective rate |
| No.102: YANG Daowen, 2025 | / | / | / | 1y | 299 | Direct Medical Cost, Direct Non-Medical Cost, Indirect Cost | Registration fee, Drug fee, Treatment fee, Nursing fee, Laboratory test fee, Transportation fee, Food and accommodation fee, Nutritional food fee, Lost wages | Utility Outcome: QALY  Effective Outcome: Number of acute exacerbation of chronic bronchitis after one year of treatment |
| No.103: Zou Hong, 2025 | / | / | / | 7d | 60 | Direct Medical Cost | Drug fee, Examination fee, Hospitalization fee | Effectiveness Outcome: Total effective rate (NHISS, Barthel) |
| No.104: LIU Guoqiang, 2021 | Healthcare System | Decision Tree | ACR ≥20%, ACR Improvement <20%. | 3m | 153 | Direct Medical Cost | Drug fee, Adverse Treatment Reaction fee | Effectiveness Outcome: Total effective rate |
| No.105: LI Kebiao, 2022 | Payer | Decision Tree | / | 4w | 610 | Direct Medical Cost | Drug fee | Effective Outcome: Clinical effective rate |
| No.106: CUI Xin, 2023 | Healthcare System | Decision Tree | Effective, Ineffective | 12w | / | Direct Medical Cost | Drug fee | Effective Outcome: Clinical effective rate |
| No.107: HU Yuer, 2023 | Societal | Decision Tree | Effective, Ineffective | 7d | / | Direct Medical Cost | Drug fee | Effective Outcome: Clinical effective rate |
| No.108: WANG Guozhen, 2020 | Healthcare System | Decision Tree | Effective, Ineffective | 30d | 1,774 | Direct Medical Cost | Drug fee | Effectiveness Outcome: Total effective rate, Adverse events |
| No.109: WANG Xianying, 2021 | Healthcare System | Decision Tree | Effective, Ineffective | 3m | 292 | Direct Medical Cost | Drug fee | Effective Outcome: ECG effective rate, Adverse events, Symptom improvement effective rate |
| No.110: LI Yunhui, 2025 | / | Decision Tree | Effective, Ineffective | 7d | 285 | Direct Medical Cost | Drug fee | Effectiveness Outcome: Total effective rate (TCM syndrome curative effect) |
| No.111: LIU Guoqiang, 2020 | Societal | / | / | 6m | 93 | Direct Medical Cost, Indirect Cost | Drug fee, Diagnostic testing fee, Bed fee, Consultation fee, Treatment fee,  Lost wages | Effectiveness Outcome: Total effective rate |
| No.112: ZHENG Qinxin, 2021 | / | / | / | 12d | 48 | Direct Medical Cost | Drug fee, Diagnostic Testing fee, Bed fee, Examination fee | Effectiveness Outcome: Total effective rate |
| No.113: LU Yunfei, 2021 | Patient's | / | / | 30d | 2,029 | Direct Medical Cost | Drug fee | Effective Outcome: Clinical effective rate |
| No.114: ZHOU Ruiyue, 2023 | Health Care Provider's | / | / | 30d | / | Direct Medical Cost | Drug fee | Effective Outcome: Clinical effective rate |
| No.115: CHEN Yu, 2025 | / | / | / | 7d | / | Direct Medical Cost | Drug fee, Examination fee, Registration fee, TCM diagnosis and treatment fee | Effective Outcome: Cough Disappearance Time |
| No.116: DING Liman, 2023 | Societal | / | / | 5-9d | 116 | Direct Medical Cost, Direct Non-Medical Cost, Indirect Cost | Drug fee, Examination fee, Laboratory fee, Material fee, Transportation fee, Lost Wages | Effectiveness Outcome: Remission time of clinical symptoms, Cure time, Cure rate, Antipyretic onset time, Complete antipyretic time, Viral nucleic acid negative rate, TCM syndrome curative effect |
| No.117: CHEN Zhongguo, 2023 | Societal | Partition Survival Model | Effective, Ineffective | 4w | 6,467 | Direct Medical Cost, Direct Non-Medical Cost, Indirect Cost | Drug fee, Registration fee, Examination fee, Consultation fee, Additional fee Due To Treatment Failure, Transportation fee, Lost Wages | Effectiveness Outcome: Total effective rate (NIH-CPSI) |
| No.118: QI Ran, 2022 | Healthcare System | Decision Tree | Effective, Ineffective | 24w | / | Direct Medical Cost | Drug fee | Utility Outcome: QALY  Effectiveness Outcome: Total effective rate |
| No.119: LI Jintian, 2022 | Payer | Decision Tree | / | 7d | 7,166 | Direct Medical Cost | Drug fee | Effectiveness Outcome: Total effective rate, Cure rate, Adverse events |
| No.120: WANG Fuping, 2024 | Healthcare System | Decision Tree | Effective, Ineffective | 90d | / | Direct Medical Cost | Drug fee | Effective Outcome: Kupperman Score |
| No.121: WANG Fuping, 2024 | Healthcare System | Decision Tree | / | 180d | / | Direct Medical Cost | Drug fee | Effective Outcome: Number of COPD exacerbation |
| No.112: CUI Xin, 2022 | Healthcare System | Decision Tree | / | 24w | 600 | Direct Medical Cost | Drug fee, Registration fee, Surgical fee, Examination fee, Treatment fee, Nursing fee, Monitoring fee, Material fee, Ward fees, Laboratory Test fee, Oxygen fee, Other Medical Cost. | Effective Outcome: NIHSS Score |
| No.123: DU Guiping, 2022 | Healthcare System | Decision Tree | Effective, Ineffective | 6m | 360 | Direct Medical Cost | Drug fee, Treatment fee | Effective Outcome: Clinical effective rate |
| No.124: CUI Xin, 2023 | Healthcare System | Decision Tree | / | 4w | 685 | Direct Medical Cost | Drug fee, Examination fee, Hospitalization fee | Effective Outcome: Clinical effective rate |
| No.125: ZHAO Dongming, 2024 | Healthcare System | Decision Tree | Effective, Ineffective | 3d | 160 | Direct Medical Cost | Drug fee | Effective Outcome: Clinical effective rate |
| No.126: WEI Zhongyi, 2024 | Healthcare System | Decision Tree | Effective, Ineffective | 12w | 205 | Direct Medical Cost | Drug fee | Effective Outcome: The Percentage of Forward Sperm Motility Before and After, Total effective rate |
| No.127: ZHANG Yanling, 2025 | Healthcare System | Decision Tree | Effective, Ineffective | 10d | / | Direct Medical Cost | Drug fee | Effective Outcome: Total effective rate |
| No.128: XU Longchen, 2025 | Payer | Markov | Mild NPDR, Moderate NPDR, Severe NPDR, PDR, Blindness | 25y | 312 | Direct Medical Cost | Drug fee | Utility Outcome: QALY |
| No.129: SHAO Wei, 2025 | Healthcare System | Decision Tree | Cured, Significantly Improved, Effective, Ineffective | 5d | 240 | Direct Medical Cost | Drug fee | Effectiveness Outcome: Disease cure rate |
| No.130: CUI Xin, 2023 | Healthcare System | Decision Tree | Effective, Ineffective | 6m | 442 | Direct Medical Cost | Drug fee, Examination fee | Effective Outcome: Clinical effective rate (Degree of relief from lower back and back pain symptoms, Degree of reduction in original symptom scores, Degree of improvement in bone density) |
| No.131: ZHANG Xuebin, 2021 | Societal | / | / | 48d | 2,180 | Direct Medical Cost, Direct Non-Medical Cost, Indirect Cost | Direct Medical fee: Drug fee, Diagnostic Test fee, Physician Service fee  Direct Non-Medical fee: Transportation fee, Accommodation and meals fee,  Indirect fee: Loss of working hours for patients and their accompanying family members | Utility Outcome: QALY  Effectiveness Outcome: The incidence of major Cardiovascular events, Improvement of seattle angina pectoris scale, TCM syndrome improvement |
| No.132: HU Jing, 2020 | Healthcare System | Decision Tree | Effective, Ineffective | 2w | 167 | Direct Medical Cost, Direct Non-Medical Cost | Laboratory Tests fee, Examinations fee, Nursing fee, Treatments fee, Hospitalization fee, Drug fee, Other fee, Adverse reaction treatment fee, Accommodation and meals fee, Transportation fee, Attendant care fee | Effectiveness Outcome: Total effective rate |
| No.133: LIU GuoQiang, 2020 | Societal | / | / | 4w | 60 | Direct Medical Cost, Indirect Cost | Drug fee, Diagnostic fee, Consultation fee, Decoction Preparation fee, Lost Wages | Effectiveness Outcome: Total effective rate (TCM syndrome Effective rate) |
| No.134: LI Xuejing, 2020 | Healthcare System | / | / | 56d | 3,893 | Direct Medical Cost | Drug fee | Effectiveness Outcome: Total effective rate for improvement of cardiac function, LVEF, 6WMT, BNP Levels |
| No.135: YU Rong, 2023 | Healthcare System | Decision Tree | Effective, Ineffective | 28d | 729 | Direct Medical Cost | Drug fee | Effectiveness Outcome: Total effective rate, ECG effective rate |
| No.136: LIU Fumei, 2021 | Health Care Provider's | Not Reported | / | 14d | 3,682 | Direct Medical Cost | Drug fee, Treatment fee | Effectiveness Outcome: Total effective rate, BI score, NIHSS Score, NDS, Plasma viscosity |
| No.137: CHEN Binbin, 2022 | Societal | Markov | CR (PASI Score Improvement ≥90%), Markedly Effective (60% ≤ PASI Score Improvement <90%),  Effective (30% ≤ PASI Score Improvement <60%), Ineffective (PASI Score Improvement <30%), Standard Treatment, No Treatment, Death. | 10y | 335 | Direct Medical Cost | Drug fee | Utility Outcome: QALY  Effectiveness Outcome: PASI |
| No.138: CUI Peng, 2022 | / | / | / | 12w | 100 | Direct Medical Cost, Indirect Cost | Bed fee, Examination fee, Drug fee, Time fee | Effectiveness Outcome: Primary Outcome: Total effective rate  Secondary Outcome: Clinical symptom improvement, Treatment safety, Tumor marker levels and positive status, Immunological evaluation indicators |
| No.139: GUAN Haijing, 2022 | Healthcare System | Decision Tree | NYHAⅡ~Ⅳ | 1y | 9.575 | Direct Medical Cost | Drug fee | Utility Outcome: QALY |
| No.140: LIN Sisi, 2024 | Healthcare System | Decision Tree+Markov | Decision Tree: Effective, Ineffective  Markov: Health, Vertebral fracture, Hip fracture, Previous vertebral fracture, Previous hip fracture, Death | Decision Tree: 6m  Markov: 50y | / | Direct Medical Cost | Drug fee, Treatment fee, Examination fee, Laboratory Test fee, Nursing fee | Utility Outcome: QALY |
| No.141: PENG Nan, 2024 | Healthcare System | / | / | 6m | 319 | Direct Medical Cost | Drug fee, Surgical fee, Examination fee, Treatment fee, Hospitalization fee, Material fee, Laboratory Test fee, Other Medical fee | Utility Outcome: QALY  Effectiveness Outcome: Osteoporosis symptom grading and quantification scale score |
| No.142: LEI Xiang, 2020 | Healthcare System | / | / | 12w | 360 | Direct Medical Cost | Drug fee,Treatment fee | Effective Outcome: Disease activity (DAS28 score, number of tender joints, number of swollen joints, and overall health), Disease control status |
| No.143: WANG Yulai, 2021 | / | / | / | 21d | / | Direct Medical Cost | Drug fee | Effective Outcome: Acute cerebral infarction, posterior circulation ischemic vertigo, basilar artery insufficiency |
| No.144: HE Yumei, 2024 | Healthcare System | Decision Tree | Effective, Ineffective | 3m | 3,114 | Direct Medical Cost | Drug fee | Effectiveness Outcome: Total effective rate |
| No.145: XIANG Heng, 2025 | Health Care Provider's | Decision Tree | / | 30d | / | Direct Medical Cost | Drug fee, Adverse Reaction Treatment fee | Effective Outcome: Clinical effective rate |
| No.146: XU Manling, 2025 | Societal | Decision Tree | Effective, Ineffective | 12w | / | Direct Medical Cost | Drug fee, Hospitalization fee | Effectiveness Outcome: Achieved or maintained clinical remission or low disease activity (DAS28 score ≤3.2 points) at the 12th week |
| No.147: CAI Jiarui, 2025 | Healthcare System | Decision Tree | Effective, Ineffective | / | / | Direct Medical Cost | Drug fee, Hospitalization fee, Laboratory Test fee, Registration fee | Effective Outcome: Clinical effective rate |
| No.148: HOU Hairuo, 2023 | / | Decision Tree | Effective, Ineffective | 4-6d | 102 | Direct Medical Cost | Drug Cost, Diagnostic testing fee (Laboratory diagnostic fee, Imaging diagnostic fee, Pathology diagnostic fee, Clinical diagnostic item fee, Etc.), Medical service fee (Decoction preparation fee, Special medication fee, Comprehensive medical service fee, TCM treatment fee, Consumables fee, Etc.) | Effectiveness Outcome: Total effective rate |
| No.149: WANG Xin, 2024 | Healthcare System | Decision Tree | Effective, Ineffective | 7d | 1,063 | Direct Medical Cost | Drug fee | Effective Outcome: Clinical effective rate |
| No.150: Zhang Hongyan, 2025 | Healthcare System | Decision Tree | / | 90d | 279 | Direct Medical Cost | Drug fee | Effective Outcome: The reduction in headache duration before and after treatment |
| No.151: HUANG Zhengming, 2025 | Healthcare System | Decision Tree | Effective, Ineffective | 12w | / | Direct Medical Cost | Drug fee | Effective Outcome: Clinical effective rate |
| No.152: XU Qian, 2022 | / | / | / | 4w | 223 | Direct Medical Cost | / | Utility Outcome: QALY  Effectiveness Outcome: Curative effect of TCM, Effect of treating angina and treating angina (Score), Total effective rate of TCM syndrome, DUKE score |
| No.153: LUO Ji, 2021 | / | / | / | 12w | 80 | Direct Medical Cost, Indirect Cost | Drug fee, Nursing fee, Treatment fee, Laboratory Test fee, Consultation fee, Lost Wages | Effectiveness Outcome: TCM syndrome curative effective rate |
| No.154: ZHANG Xuebin, 2021 | Societal | / | / | 52w | 5,094 | Direct Medical Cost, Direct Non-Medical Cost, Indirect Cost | Medical Service fee, Examination fee, Drug fee, Transportation fee, Nutritional fee, Meal fee, Lost wages | Utility Outcome: QALY  Effectiveness Outcome: 52-week cardiovascular events, Total effective rate of angina pectoris, weekly average of angina pectoris, VAS score, SAQ score |
| No.155: ZHENG Hang, 2023 | / | / | / | 2w | / | Direct Medical Cost | Drug fee | Effectiveness Outcome: Total effective rate |
| No.156: PANG Hongbo, 2022 | / | / | / | 2w | 400 | Direct Medical Cost | Drug fee, Examination fee | Effectiveness Outcome: Prevention and treatment effective rate |
| No.157: YANG Chunmei, 2020 | Societal | Markov | No angina attacks, Angina attacks monthly, Angina attacks weekly, Angina attacks daily, Death | 1y | 2,443 | Direct Medical Cost, Direct Non-Medical Cost, Indirect Cost | Drug fee, Registration fee, Examination fee, Transportation fee, Lost Wages, Other fee | Utility Outcome: QALY |
| No.158: Zhou Bin, 2023 | Health Care Provider's | Decision Tree | Significant effect, Effective, Ineffective | / | 40 | Direct Medical Cost | Drug fee | Effectiveness Outcome: Total effective rate |
| No.159: LI Chang, 2024 | Healthcare System | Decision Tree | Effective, Ineffective | 28d | / | Direct Medical Cost | Drug fee | Effective Outcome: Clinical effective rate, ECG effective rate |
| No.160: WANG Yizhu, 2024 | Healthcare System | Decision Tree | / | 28d | / | Direct Medical Cost | Drug fee, Medical Service fee, Treatment fee, Examination fee | Effective Outcome: Clinical effective rate |
| No.161: WANG XiaoYan, 2024 | Healthcare System | Decision Tree | / | 28d | 1,411 | Direct Medical Cost | Drug fee | Effective Outcome: Pelvic mass disappearance |
| No.162: DING Yanbing, 2025 | Healthcare System | Decision Tree | Effective, Ineffective | 14-42d | 656 | Direct Medical Cost | Drug fee | Effectiveness Outcome: Total effective rate |
| No.163: REN Huijun, 2024 | Payer | / | / | / | / | Direct Medical Cost, Indirect Cost | Drug fee, Hospitalization fee, Lost Wages | Effectiveness Outcome: Total effective rate |
| No.164: MA Haiqing, 2021 | / | / | / | 4w | 93 | Direct Medical Cost | Drug fee | Effectiveness Outcome: Total effective rate |
| No.165: HUANG Qian, 2020 | / | / | / | 14d | 2,422 | Direct Medical Cost | Drug fee, Hospitalization fee | Effectiveness Outcome: Total effective rate |
| No.166: ZHANG Xueyi, 2021 | / | / | / | 14d | 151 | Direct Medical Cost | Drug fee, Treatment fee, Bed fee, Other fee | Effectiveness Outcome: NIHSS, MRS, BI, TCM syndrome scale, Level of Interleukin-1β, Interleukin-6, Tumor necrosis factor alpha |
| No.167: LI Na, 2021 | / | / | / | 5d | 88 | Direct Medical Cost | Drug fee | Effectiveness Outcome: Total effective rate |
| No.168: JIA Jianying, 2023 | Healthcare System | / | / | 1m | 120 | Direct Medical Cost | Drug fee | Effectiveness Outcome: Total effective rate (TCM syndrome curative effect) |
| No.169: LIU Yu, 2020 | / | / | / | 4w | 93 | Direct Medical Cost | Drug fee | Effectiveness Outcome: Total effective rate |
| No.170: ZHU Jingyan, 2020 | / | / | / | 90d | 98 | Direct Medical Cost | Drug fee, Examination fee, Rehabilitation fee | Effectiveness Outcome: MRS, BI |
| No.171: HUANG Haitao, 2022 | Healthcare System | / | / | 60d | / | Direct Medical Cost | Drug fee | Effectiveness Outcome: Total effective rate |
| No.172: WANG Xingdong, 2021 | / | / | / | 14d | 94 | Direct Medical Cost | Drug fee, Examination fee, Hospitalization fee, Treatment fee | Effective Outcome: Total effective rate, Adverse events |
| No.173: SONG Miaoyuan, 2022 | / | / | / | 7d | 83 | Direct Medical Cost | Drug fee, Treatment fee | Effectiveness Outcome: Total effective rate (Symptoms include cough and wheezing, auscultation reveals moist rales in the lungs) |
| No.174: WANG Xiu, 2023 | / | / | / | 14d | 160 | Direct Medical Cost | Drug fee | Effectiveness Outcome: ECG effective rate, Angina pectories effective rate |
| No.175: PAN Zhehao, 2023 | / | / | / | 10d | 88 | Direct Medical Cost, Direct Non-Medical Cost, Indirect Cost | Drug fee, Diagnostic Testing fee, Hospitalization fee, Treatment fee, Monitoring fee, Surgical fee, Consultation fee, Nursing fee, Nutritional fee, Transportation fee, Accommodation and meal fee, Lost Wages | Effectiveness Outcome: Local ecchymosis resolution time, Functional impairment recovery time, local pain resolution time |
| No.176: Feng Rongwei, 2020 | / | / | / | / | 200 | Direct Medical Cost | Drug fee, Hospitalization fee, Diagnostic Test fee, Injection fee | Effectiveness Outcome: Total effective rate, BI, NIHSS, Hematological rheology parameters, adverse events |
| No.177: LI Meifang, 2024 | Healthcare System | / | / | 7d | 467 | Direct Medical Cost | Drug fee | Effectiveness Outcome: Total effective rate, Total effective rate of TCM syndromes |
| No.178: YANG Qian, 2024 | / | / | / | 28d | / | Direct Medical Cost | Drug fee | Effectiveness Outcome: Total effective rate, upper abdominal distension score, belching score, loss of appetite score, early satiety score, epigastric pain score |
| No.179: LU Yunfei, 2021 | / | / | / | 90d | 257 | Direct Medical Cost | Drug fee, Hospitalization fee, Examination fee | Effectiveness Outcome: TCM syndrome score, level sf TBIL, ALB, TBA |
| No.180: CHEN Yi-peng  et al. 2021 | / | / | / | 5d | 268 | Direct Medical Cost | Drug fee, Hospitalization fee, Treatment fee | Effectiveness Outcome: Blood Loss Per unit time and total blood loss during surgery drainage volume within 24 hours postoperatively, drainage volume between 24 and 48 hours postoperatively, and total drainage volume within 48 hours postoperatively, number of days until postoperative drainage tube removal, length of postoperative hospitalization and number of days spent bedridden at home after discharge |
| No.181: LIU Zhihui, 2022 | / | / | / | 6m | 360 | Direct Medical Cost | Drug fee | Effectiveness Outcome: NIHSS |
| No.182: XU Lingjun, 2022 | / | / | / | 7d | 112 | Direct Medical Cost, Indirect Cost | Drug fee, Examination fee, Lost Wages | Effectiveness Outcome: Percentage of people without colds within 1 week |
| No.183: HUA Xiangxiang, 2021 | Patient's | / | / | 5d | 198 | Direct Medical Cost | Drug fee | Effectiveness Outcome: Total effective rate |
| No.184: LIU Huan, 2020 | / | Decision Tree | / | 6d | 606 | Direct Medical Cost, Direct Non-Medical Cost, Indirect Cost | Drug fee, Registration fee, Consultation fee, Laboratory fee, Treatment fee, Transportation fee, Lost wages | Effectiveness Outcome: TCM syndrome scale score |
| No.185: CHEN Wenwen, 2024 | Healthcare System | Decision Tree | Effective, Ineffective | 6d | 834 | Direct Medical Cost | Drug fee | Effectiveness Outcome: Clinical effective rate (Patient symptoms, signs, laboratory tests, and pathogen detection indicators) |
| No.186: WANG Zhijun, 2024 | Healthcare System | Decision Tree | Effective, Ineffective | 4-16w | / | Direct Medical Cost | Drug fee | Effectiveness Outcome: Clinical effective rate, ECG effective rate |
| No.187: QU Detao, 2023 | / | / | / | 14d | 186 | Direct Medical Cost | Drug fee, Hospitalization fee, Treatment fee, Laboratory Test fee, Nursing fee, Medical Service fee | Effectiveness Outcome: Clinical effective rate, NHISS, mRS, adverse events |
| No.188: XIONG Liping, 2020 | / | / | / | 14d | 66 | / | / | Effectiveness Outcome: Total effective rate, NHISS, adverse events |
| No.189: ZHANG Yaxuan, 2022 | / | / | / | 3d | 692 | Direct Medical Cost | Drug fee | Effectiveness Outcome: Clinical effective rate |
| No.190: WANG Qingqing, 2025 | / | / | / | 2m | 1,512 | Direct Medical Cost | Drug fee | Effectiveness Outcome: Total effective rate (TCM syndrome curative effect) |
| No.191: XU Huanxiang, et al.2020 | / | / | / | 7d | 86 | Direct Cost, Direct Non-Medical Cost, Indirect Cost | / | Effectiveness Outcome:  Total effective rate |
| No.192: WANG FENG, et al.2021 | / | / | / | 12w | 156 | / | / | Effectiveness Outcome: Clinical effective rate, PSQI |
| No.193: Zuo Kaini, 2020 | / | / | / | 6m | 4,915 | Direct Medical Cost | Drug fee | Effectiveness Outcome: Total effective rate |
| No.194: ZHANG Yaxuan, 2022 | / | / | / | 2m | 580 | Direct Medical Cost | Drug fee | Effectiveness Outcome: Total effective rate |
| No.195: TAN Shurao, 2021 | / | / | / | 2m | 100 | Direct Medical Costs | / | Effectiveness Outcome: TCM symptoms and signs, red blood cell count in urine sediment microscopy, quantitative analysis of 24h urine, renal function, adverse events |
| No.196: HUANG Shaohua, 2025 | / | / | / | / | 150 | Direct Medical Costs | Drug fee, Laboratory Test fee, Medical Service fee | Effectiveness Outcome: effective rate after 14 days |
| No.197: ZHANG He, 2025 | / | / | / | 7d | 120 | Direct Medical Costs | Drug fee, Treatment fee | Effectiveness Outcome: Total effective rate |
| No.198: LV Xiaoqin, 2020 | / | / | / | 6w | 90 | Direct Medical Costs | Drug fee | Effectiveness Outcome: Total effective rate |

d: day, m: month, y: year, QALY: quality-adjusted life years, ECG: Electrocardiogram, MACCE: Major Adverse Cardiac and Cerebrovascular Events, ISI: Insomnia Severity Index, PSQI: Pittsburgh Sleep Quality Index, WPAI: Work Productivity And Activity Impairment, NIHSS: National Institutes of Health Stroke Scale, CR: Complete Response, PR: Partial Response, SD: Stable Disease, PD: Progressive Disease, ORR: Objective Response Rate, CT: Computed Tomography, CVD: Cardiovascular Disease, CRR: Complete Response Rate, LVEF: Left Ventricular Ejection Fraction, LVEDD: Left Ventricular End-Diastolic Diameter, LVESD: Left Ventricular End-Systolic Diameter, 6MWD: 6-Minute Walking Distance, MRS: Modified Rankin Scale, IPSS: International Prostate Symptom Score, Hs-CRP: Hypersensitive-C-Reactive-Protein, TNF-α: Tumor Necrosis Factor-alpha, IL-6: Interleukin-6, IL-4: Interleukin-4, BI: Barthel Index, SFR: Stone-Free Rate, HP: Helicobacter Pylori, ADL: Activity of Daily Living, CTnI: Cardiac Troponin I, NT-proBNP: N-terminal pro-B-type natriuretic peptide, CCEs: Composite Cardiac Events, MLHFQ: Minnesota Heart Failure Quality of Life Scale, CK-MB: Creatine Kinase Isoenzymes, VAS: Visual Analogue Method, CD3+: Cluster of Differentiation 3‌, CD4+: Cluster of Differentiation 4, CD8+: Cluster of Differentiation 8, MIC: Minimum Inhibitory Concentration, NIH-CPSI: National Institutes of Health Chronic Prostatitis Symptom Index, BNP: B-Type Natriuretic Peptide, NDS: Neurological Disability Score, MSQoL:Migraine-Specific Quality of Life, HADs: Headache Attack Days, HIT: Headache Impact Test, DEPIQ: The Deficiency And Excess Pattern Identification Questionnaire, CHPIQ: The Cold And Heat Pattern Identification Questionnaire, BSPQ: The Blood Stasis Pattern Questionnaire, ACR: American College of

Rheumatology Improvement, DAS28 : Disease Activity Score for 28 joints, IL-1β: Interleukin-1β, SAQ: The Seattle Angina Questionnaire, TBIL: Total Bilirubin, ALB: Albumin, TBA: Total Bile Acids.

**Table S3. Incremental analyses results and sensitive analyses of included studies.**

| **Study ID** | Incremental Analyses | Thresholds | Discount | Sensitive Analyses | Sensitivity Analyses Variables |
| --- | --- | --- | --- | --- | --- |
| No.1: LongChen, Xu, 2023 | ICUR = ¥ 15,147/QALY | GDP | 5% | DSA+PSA | Cost and utility values for each disease state:±10% and discount rate fluctuated between 0 to 10% |
| No.2: Cong Wang, 2025 | Group A vs. Group C:  ICUR= $ 3,147/QALY (55-59 years)  ICUR= $ 2,313/QALY (60-64 years)  ICUR= $ 1,886/QALY (65-69 years)  ICUR= $ 1,221/QALY (70-74 years)  Group B vs. Group C:  ICUR= $ 11,955/QALY (55-59 years)  ICUR= $ 9,858/QALY (60-64 years)  ICUR= $ 10,731/QALY (65-69 years)  ICUR= $ 9,711/QALY (70-74 years) | GDP | 3% | DSA+PSA | Relative risk of fracture in individuals with osteoporosis, initial BMD, the therapeutic effect of drugs, cost, Health utility, and discount rate. |
| No.3: Yumei He, 2023 | ICUR = ¥ 63,001/QALY | GDP | 5% | DSA+PSA | Drug cost, efficacy, state transition probabilities, and mortality rate parameters fluctuated within 95% CI from the reference literature or baseline values±30% variation. |
| No.4: Lu Wang, 2025 | ICUR:= ¥ 57,381.85 /QALY | GDP | 5% | DSA+PSA | Cardiovascular mortality rate, drug cost, hospitalization cost, hospitalization rate, utility value parameters within 95% CI, and discounting rate fluctuated between 0~8% |
| No.5: Liang Lu, MM, 2022 | ICER:  CT, SYI vs.CT: ¥ 6,766.47,  CT, SYI, L-C vs.CT, SYI: ¥ 7,606.79,  CT, SYI, CD vs.CT, SYI, L-C: ¥ 4,907.50  CT, SI, H vs.CT, SYI, CD: ¥ 6,909.54,  CT, SYI, SMI vs.CT, SI, H: ¥ 14,400.79,  CT, SYI, AC vs.CT, SYI, SMI: ¥ 6,780.25  CT, SI, DS vs.CT, SYI, AC: ¥ 4,010.74  CT, SI vs.CT, SI, DS: ¥ 3,614.18 | / | 5% | DSA+PSA | Efficacy rate of the 9 treatment regimens:±5%, and  drug cost fluctuated within highest and lowest manufacturer retail price in China. |
| No.6: Yang shuo, 2024 | ICER = ¥ 495. 97 | CDI | / | DSA+PSA | Unit price of Zhizhuanling Oral Solution, average cost of  asthma treatment, patient medication adherence, patient medication duration fluctuated within 95% CI or±20% fluctuation range of the baseline value, and efficacy parameters fluctuated within upper and lower limits |
| No.7: Guoqiang Liu, 2024 | 1 year:  ICER= ¥ 74,653 (MACCE)  ICUR= ¥ 115,408/QALY  16 years:  ICUR= ¥ 12,421/QALY | GDP | 5% | DSA+PSA | Cost parameters and clinical outcome fluctuated based on the 95% CI |
| No.8: YANG Xuanru, 2024 | ICER= ¥ 293.48 | CDI | / | DSA+PSA | / |
| No.9: Junjie Zhu, 2024 | ICUR= $ 476.41/QALY | GDP | 5% | DSA+PSA | Drug acquisition cost, follow-up cost, disease management cost, subsequent treatment cost, end life cost, health utility and clinical parameter. |
| No.10: ZHAO Xiaoxiao, 2023 | ICER= ¥ 238.84 | CDI | / | DSA+PSA | Drug cost, patient medication adherence, and efficacy parameters fluctuated±10% |
| No.11: Jie Pan, 2024 | ICUR= ¥ -2628.13/QALY | GDP | 5% | DSA+PSA | Cost utility, probability of each transfer, variable ranges (95% CI or±25% of base-case values), the annual discount rate of cost and utilities: 0% to 8%, time horizon: 10 years to 30 years. |
| No.12: Yake Lou, et al. 2025 | ICUR= ¥ 54,522/QALY | GDP | 5% | DSA+PSA | CV mortality, drug cost, hospitalization cost, and utility weights varying model inputs within their 95% CI |
| No.13: WU Xue, 2024 | ICER:  Jiwei Xifeng Granule vs Shaoma Zhijing Granule = ¥ 169.95  Shaoma Zhijing Granule plus Conventional Standard Treatment vs.Jiwei Xifeng Granule plus Conventional Standard Treatment = ¥31.20,  Shaoma Zhijing Granule plus Conventional Standard Treatment vs.Changma Xifeng Tablets plus Conventional Standard Treatment = ¥6,334.06 | GDP | / | DSA+PSA | / |
| No.14: Yang Li, 2021 | ICER= ¥ 3,885.75 | GDP | 5% | DSA+PSA | Treatment efficacy:±5%, and drug cost fluctuated within highest and the lowest manufacturer retail price. |
| No.15: Zhou Wenxin, 2023 | ICUR= ¥ 84,871.48/QALY | GDP | 5% | DSA+PSA | The proportions of patients in each state after treatment, cost, transition probabilities, and utility values were determined. If 95% CI were reported in the original data or previous literature, these were used as the upper and lower limits. If not, a range of ±10% of the estimated value was used as the upper and lower limits. |
| No.16: CHEN Zijia, 2024 | ICER= ¥ 2,678.67 | CDI | / | DSA+PSA | / |
| No.17: CHENG lili, 2022 | ICUR= ¥ 73,301.12  ICER= ¥ 48.33 | ICUR:GDP  ICER: 57.33 per 1% four-week Effective Rate (Threshold based on patient willingness-to-pay survey results) | / | DSA+PSA | Effective rate and utility value (with 95% CI parameter fluctuation value). |
| No.18: PAN Huimin, 2024 | ICUR= ¥ 264,827.7648/QALY | GDP | / | DSA+PSA | Drug cost ±20%, and the upper and lower limits of the 95% CI for probability and health utility value parameters. |
| No.19: Kai Xu, 2025 | Group A: ICER= ¥ 43.38  Group B: ICER= ¥ 29.68  Group C: ICER= ¥ 56.94  Group D: ICER= ¥ 19.56  Group E: ICER= ¥ 28.08 | / | / | DSA+PSA | The values for drug cost, other cost and efficacy parameters were adjusted to be within ±20% of their baseline values. |
| No.20: LIANG Liang, 2024 | ICUR= ¥ 28,137.42883/QALY | GDP | 5% | DSA+PSA | The cost and utility values for each state, and adjustments to the discount rate from 0% to 10%. |
| No.21: WAN Feng, 2023 | Natural delivery group:  ICER = ¥ 94.11 (postpartum hemorrhage rate)  ICER = ¥ 58.35 (improvement in fundal height at 72 hours postpartum)  ICER = ¥ -17.58 (incidence of poor uterine involution)  ICER = ¥ 58.35 (duration of lochia)  Cesarean section group:  ICER = ¥ -8,003.20 (postpartum hemorrhage rate)  ICER = ¥ 1,755.43 (improvement in fundal height at 72 hours postpartum)  ICER = ¥ -102.21 (incidence of poor uterine involution)  ICER = ¥ 403.75 (duration of lochia) | / | / | DSA | Effect parameters, drug cost, and non-drug cost fluctuated within±20%. |
| No.22: ZHANG XinYing, 2024 | ICER= ¥ 21.65 | CDI | / | DSA | Effect parameters and drug cost parameters fluctuated within±10% from the base value. |
| No.23: LIANG wanxian, 2022 | ORR:  ICER= ¥ 5,801.0 (compound cantharidin Capsule plus TACE versus TACE)  ICER= ¥ -31,416.0 (Yangzheng Xiaoji Capsule plus TACE versus compound cantharidin Capsule plus TACE)  ICER= ¥ −40,520.6 (Sophora Flavescens Granule plus TACE versus Compound Mylabris Capsule plus TACE)  CRR:  ICER= ¥ 239,718.6 (compound cantharidin Capsule plus TACE versus TACE)  ICER= ¥ 178,960.4 (Yangzheng Xiaoji Capsule plus TACE versus compound cantharidin Capsule plus TACE)  ICER= ¥ −796,974.0 (Sophora Flavescens Granule plus TACE versus Compound Mylabris Capsule plus TACE) | / | / | DSA+PSA | / |
| No.24: ZHAO Yifan, 2022 | FOLFOX: ICER = ¥ 23,223.53  CapeOX: ICER = ¥ 34,245.34  FOLIRI: ICER = ¥ 26,669.46 | GDP | / | DSA | Drug cost decreased by-10%, and other cost increased by 10%. |
| No.25: Yuliang Xiang, 2021 | ICUR = ¥ 14,866.06 / QALY | GDP | 5% | DSA+PSA | Price of the two drugs, clinical outcome, probability of each transfer, and utility value of the two drugs after treatment fluctuated within 95% CI or±10% of the base case values |
| No.26: Lisong Yang, et al. 2020 | / | / | / | / | / |
| No.27: Meng-Bin Tang, 2024 | ICER= NT$ 880,908 (all populations)  ICER= NT$ -2,419,623 (stage I)  ICER= NT$ 994,722( stage II)  ICER= NT$ 568,869 (stage III)  ICER= NT$ 724,791 (stage IV) | GDP | 3% | / | / |
| No.28: Gordon Liu, 2021 | / | / | / | / | / |
| No.29: ZHAN Xudan, 2024 | / | / | / | DSA | Drugs cost decreased by 10%, while bed fees, treatment fees, nursing fees, and herbal decoction fees each increased by 5%. |
| No.30: CAO ShiHuan, 2024 | ICER= ¥ 73.76 (Lianhua Qingwen combination therapy vs. Conventional Standard Treatment)  ICER= ¥ 985.64 (Jinhua Qinggan combination therapy vs.Conventional Standard Treatment)  ICER=¥ 936.50 (Xuanfei Baidu Decoction combination therapy vs. Conventional Standard Treatment) | / | / | DSA+PSA | Drug cost fluctuated within a 10% range as the upper and lower limits. The treatment duration range is estimated based on the number of days specified in drug package inserts and research literature from the Pharmaceutical Intelligence Network. For the conventional treatment group, the cough resolution rate and RR value utilize 95% CI as the upper and lower limits of the range. |
| No.31: Wei Zhongyi, 2024 | ICER=¥ 100.94 | CDI | / | DSA | Drug cost fluctuate by up to 20%. |
| No.32: DAI Yingying, 2023 | ICUR= ¥ 18,519.24/QALY | GDP | 5% | DSA+PSA | The cost parameters were set within a range of 20% above and below the baseline value, while the transition probability and utility value parameters were set within a range of 10% above or below the baseline value. |
| No.33: YANG Shuo, 2023 | ICER= ¥ 102.42 | CDI | / | DSA+PSA | The cost, patient medication time, and efficacy parameters use the 95% CI of the baseline values or values fluctuating by±20% as the upper and lower limits of the parameters. |
| No.34: Ming Hu, 2022 | ICUR= ¥ 12,051.27/QALY | GDP | 5% | DSA+PSA | Various cost and patient status transition probabilities, with parameters:±10%, and discount Rate ranging from 0% to 8%. |
| No.35: Qian Xu et al., 2020 | ICUR= ¥ 22,443.32($ 3,390.23)/QALY | GDP | 5% | DSA+PSA | Cost, utility, transfer probability fluctuated by 10%, and  discount rate fluctuated by 1% ~ 8% |
| No.36: SUN Yang, 2024 | ICUR= ¥ 2,452.39/QALY | GDP | 5% | DSA | Drug prices, hospitalization cost, effectiveness Rate, and utility values for each health state ±10%, and discount Rate ranging from 0-10%. |
| No.37: LI Mengyuan, 2025 | ICUR= ¥ 73,231.75/QALY (Decision Tree)  ICUR= ¥ -4,412.70/QALY (Markov) | GDP | 5% | DSA+PSA | The mortality rate for mRS 0-2 and mRS 3-5 were allowed to fluctuate by ±10%, the drug price was allowed to fluctuate by ± 20%, and the discount rate was varied within the range of 0-8%. |
| No.38:XIAO GuiRong, 2021 | / | / | / | DSA | Drug cost was reduced by 10%. Other costs including medical service fee, laboratory fee, treatment fee, direct non-medical fee, and indirect fee were increased by 10%. |
| No.39: WEI Chunyan, 2022 | ICER= ¥ -38.47 (The Clinical Effective Rate)  ICER= ¥ -38.47 (Serum creatinine)  ICER= ¥ 534.17 (Urea nitrogen) | The minimum treatment cost per unit of urea nitrogen for each stage of CKD is ¥ 13,916.42, which is used as the threshold.  (This value is obtained through a retrospective survey of medical expense data, using the minimum treatment cost as the threshold.) | / | DSA | The 95% CI for the mean difference in serum creatinine and blood urea nitrogen levels was calculated, and the prices of Shenshuining Capsule and other medications were reduced by 10%. |
| No.40: CAO ShiHuan, 2023 | ICER= ¥ 437.84 | / | / | DSA+PSA | Drug prices, treatment duration, and RR values fluctuated within 95% CI. |
| No.41: LU Zhenkai, 2024 | ICER= ¥ 1345. 43 | CDI | / | DSA+PSA | / |
| No.42: LIU Teng, 2021 | ICUR= ¥ 111,909/QALY | GDP | / | DSA+PSA | Drug prices, lost work time and health utility value fluctuated by 20%. |
| No.43: CUI Mancang, 2020 | ICER= ¥1,682.52 | / | / | DSA+PSA | Drug prices fluctuated by 10%. |
| No.44: LIANG WanXian, 2024 | ICER= ¥ -1,294.2 (Group A)  ICER= ¥ 986.7 (Group B) | / | / | DSA+PSA | The drug price was fluctuated within 10% of the baseline value, the range of treatment days is derived from the treatment durations reported in the literature, and the range of RR values comes from the 95% CI. |
| No.45: Pengli Su, 2024 | ICUR= $29,588.91/QALY | GDP | / | DSA+PSA | Cost, transition probabilities, all-cause mortality rate, and frequency. |
| No.46: CHEN Zijia, 2024 | ICER= ¥ 15.10 | CDI | / | DSA+PSA | The drug unit price, basic cost of gingivitis treatment, patient medication duration, patient medication adherence, and efficacy parameters were based on the 95% CI of the baseline values or a 10% fluctuation on baseline values. |
| No.47: LIU Huan, 2022 | ICER = ¥ 263.71 | CDI | / | DSA+PSA | Cost (drug cost, treatment cost) and efficacy parameter variation ranges shall be within the 95% CI of the baseline value or fluctuate within±20% of the baseline value. |
| No.48: WANG Zhi-heng, 2021 | ICUR= ¥ -2,283,583.96/QALY | GDP | 5% | DSA+PSA | The utility value variable adopts the upper and lower bounds from the literature, while all other variables have a variation range of mean±10%. |
| No.49: GUO ZhaoTing, 2021 | ICUR= ¥ 39.91/QALY | GDP | 5% | DSA+PSA | The 95% CI is used as the upper and lower bounds for cost and patient state transition probabilities. If no CI is available, a fluctuation of 10% of the estimated value. |
| No.50: Hu Haiyao, 2022 | ICER= ¥ 8.34 | WTP threshold of ¥ 15.00 as determined by questionnaire surveys of patients. | / | DSA | Cost was fluctuated within 10% range, including drug cost, medical cost, etc. |
| No.51: LI Han, 2020 | ICER= ¥ -1,860.47 (Shuxuening injection (trade name Xingxue) vs.Ginkgo biloba extract injection group)  ICER= ¥ 6,494.63 (Ginkgo biloba extract injection group vs.Ginkgo biloba extract injection (trade name Ginaton) ) | / | / | DSA | Cost decreased by 10%, and Total effective rate decreased by 10%. |
| No.52: SHI Suisui, 2023 | / | / | / | / | / |
| No.53: WANG Zhuojue, 2022 | / | / | / | DSA | The lower and upper limit of the 95% CI for Total effective rate. |
| No.54: ZHOU Li, 2020 | / | / | / | DSA | Drug cost fluctuate by 10%. |
| No.55: Xiang Gao, 2024 | / | / | / | / | / |
| No.56: Sun Yan, 2025 | ICER= ¥ 816.48 | CDI | / | / | / |
| No.57: XIA Ruyu, 2022 | ICER= ¥ 1,199.18 (Group A vs. Group H)  ICER= ¥ 3,705.22(Group C vs. Group A)  ICER= ¥ 1,034.06(Group B vs. Group H)  ICER= ¥ 3,113.84(Group A vs. Group B)  ICER= ¥ -659.76(Group D vs. Group A) | / | / | DSA+PSA | / |
| No.58: LEI Chao, 2022 | ICER= ¥ 7,051.79 | CDI | / | DSA+PSA | The medication duration for patients fluctuate by 20% around the baseline value. Drug cost, patient compliance, and efficacy parameters each exhibit a 95% CI variation. |
| No.59: HU Xingmao, 2020 | / | / | / | DSA | All cost (15% variation), overall patient response rate (3% variation) |
| No.60: SHENG Ye, 2021 | Phase II Clinical Trial:  ICER= ¥ 20.00 (The efficacy index of angina pectoris)  ICER= ¥ 11.55(the Effective Rate of ECG change)  ICER= ¥ 34.32 (the nitroglycerin arrest rate),  ICER= ¥ 50.45(the efficacy of TCM syndrome)  Phase III Clinical Trial:  ICER= ¥ 102.75 (The efficacy index of angina pectoris)  ICER= ¥ 36.36(the Effective Rate of ECG change)  ICER= ¥ -157.01 (the nitroglycerin arrest rate)  ICER= ¥ -145.45 (the efficacy of TCM syndrome)  ICUR= ¥ 80,355 /QALY | GDP | / | DSA+PSA | Drug cost, patient wage losses, and utility values are subject to±20% estimation margins. |
| No.61: ZHANG Lidan, 2022 | ICER= ¥ 222.07 | CDI | / | DSA+PSA | The treatment cost, drug unit prices, and efficacy parameters use the 95% CI of the baseline values or a 20% fluctuation above and below the baseline values as the upper and lower limits of the parameters. |
| No.62: ZHENG Yingying, 2021 | ICER= ¥ 566.73 | / | / | DSA | The drug price is fluctuate within the highest and lowest possible prices, with the effect variation range set at 5%. |
| No.63: ZHANG Shiqin, 2025 | / | / | / | DSA | Drug cost and efficacy indicators exhibit a 95% CI variation. |
| No.64: LI Wei, 2021 | ICUR= ¥ 74,479/ QALY | GDP | 5% | DSA+PSA | Hospitalization cost, post-discharge rehabilitation cost, and secondary prevention cost, and the unit price of drug and the range of utility value changes under different health states, were obtained from literature or databases. For other parameters, a 20% fluctuation above and below the base value was used as the upper and lower limits for these parameters. And discount rate was ranging from 0% to 8%. |
| No.65: WANG Zhiheng, 2021 | ICUR= ¥ -102.52 /QALY | GDP | 5% | DSA+PSA | The ranges of the transition probability parameters, cost parameters, and utility value parameters were adjusted accordingly, the discount rate was adjusted within the range of 0-10%, and the initial proportions of states 0-2 in the experimental group and the control group were adjusted upwards and downwards by 20%. |
| No.66: KOU Zhenzhen, 2023 | ICER= ¥-4.13 (Group A vs. Group B)  ICER= ¥-22.72(Group B vs. Group C)  ICER= ¥ 6.73 (Group C vs. Group D) | / | / | DSA | Drug cost reduced by 10%. |
| No.67: GUO Xiaojie, 2020 | / | / | / | DSA | Drug cost reduced by 10%. |
| No.68: ZHEN Hui, 2021 | ICER= ¥ 4.80  ICUR= ¥ 13,689.09/QALY | ICUR: GDP | / | DSA | Changes in total cost, clinical cure rate, and QALY within 10% of the original data. |
| No.69: LI Xuan, 2022 | ICER= ¥ 2.80 (improving TCM syndromes)  ICER= ¥ 4.99 (constipation symptoms) | 100 (One published economic evaluation study related to upper respiratory tract infections was referenced.) | / | DSA+PSA | Various cost and cure rate (within a 95% CI or±20% range). |
| No.70: GAN Xiong, 2023 | ICER= ¥ -75.58 (The median reduction rate of NT-proBNP)  ICER= ¥ 290.79 (the incidence of CCEs)  ICER= ¥ -146.54 (the Total effective rate of cardiac function classification)  ICER= ¥ -915.13 (the change of LVEF of the heart)  ICER= ¥ -66.67 (the total score improvement value of the MLHFQ)  ICER= ¥ -82.17 (6MWD) | / | / | DSA | Drug prices will be reduced by 10%, while other cost will fluctuate by 10%. |
| No.71: LIU Zhaohui, 2024 | ICUR= ¥ 12,132/QALY  ICER= ¥ -131.87 (VAS)  ICER= ¥ 6.33 (the recovery rate of diseases)  ICER= ¥ 8.42 (the recovery rate of traditional Chinese)  ICER= ¥ 5.37 (the recurrence rate of pelvic pain) | GDP | / | DSA | Cost , effectiveness and utility data fluctuate by 5%. |
| No.72: CUI Xin, 2022 | ICER= ¥ 6,827.59 | CDI | / | DSA+PSA | For medication cost, treatment cost, patient adherence, days of medication, and efficacy parameters, the 95% CI of the baseline value or a 20% fluctuation above and below the baseline value was used as the upper and lower limits of the parameters. |
| No.73: WEI Ruili, 2020 | ICER= ¥ 95.89 | CDI | / | DSA+PSA | Treatment cost uses a 10% fluctuation range around the base value as the parameter upper and lower limits, unit price of Qidongyi Oral Liquid is based on the highest and lowest bid values from Menet as the parameter upper and lower limits, and the two efficacy parameters use the maximum and minimum values from the meta-analyses as the parameter upper and lower limits. |
| No.74: CUI Xin, 2023 | ICER= ¥ 1,982. 69 | CDI | / | DSA+PSA | / |
| No.75: CUI Xin, 2023 | ICER= ¥ 1,982. 69 | CDI | / | DSA+PSA | / |
| No.76: LIU Jifang, 2023 | ICER= ¥ 2.47 | GDP | / | DSA+PSA | Drug cost are determined based on the highest and lowest winning bid prices over the past three years, while other cost and effectiveness indicators are determined by allowing for a 5% fluctuation above and below the baseline. |
| No.77: WANG Fangxu, 2023 | Group B vs. Group A: ICER= ¥ 3.46  Group C vs. Group A: ICER= ¥ 11.55 | / | / | DSA+PSA | The cost parameters were set with a range of±20% variation, and the treatment efficacy and adverse event rate were taken from the upper and lower limits of the 95% CI. |
| No.78: PAN Jie, 2021 | ICUR= ¥-3,466/QALY | GDP | 5% | DSA+PSA | The ranges of the transition probability parameters and utility value parameters were adjusted accordingly, the discount rate was adjusted from 0% to 10%, the RR varied from 0.22 to 0.91, cost were adjusted by 10%, and medication adherence rate were adjusted from 50% to 100%. |
| No.79: CUI Xin, 2023 | ICER= ¥ 655.2 | CDI | / | DSA+PSA | The cost of conventional treatment and the number of days patients take medication were varied within a range of 20% of the baseline values, while effective rate of different interventions was varied within a range of 5% of the baseline values. |
| No.80: XU Haiting, 2024 | ICUR= ¥ -487,9.83/QALY | GDP | 5% | DSA+PSA | Cost, transition probabilities, and utility values were varied within 95% CI or maximum/minimum ranges, and the discount rate was varied within the range of 0-8%. |
| No.81: LI Fan, 2020 | ICER= ¥ 245.49 | / | / | DSA | Drug prices was reduced by 10%, and effective rate was reduced by 10%. |
| No.82: LIU Xing, 2020 | ICER= ¥ 28,002.9 | / | / | DSA | Drug prices reduced by 10%. |
| No.83: Ma Luyao, 2023 | ICER= ¥ 23.73 (Group A)  ICER= ¥ 34.03 (Group B) | GDP | / | DSA | Drug prices reduced by 10%. |
| No.84: LIU Dan, 2022 | / | / | / | DSA | The price of drugs was fluctuated by 20% |
| No.85: WANG Yaqin, 2021 | ICER= ¥ 126.34 | GDP | / | DSA | Drug prices increased by 20%, while the cost of managing adverse reactions and the difference in clinical efficacy decreased by 50%. |
| No.86: XU Longchen, 2022 | Group A: ICER= ¥ 0.35  Group B: ICER= ¥ 1.70  Group C: ICER= ¥ 15.07/2.70 | / | / | DSA+PSA | Drug prices reduced by 10%. |
| No.87: MA Luyao, 2022 | Total effective rate:  Group A: ICER= ¥ 7.70  Group B: ICER= ¥ 8.45  Group C: ICER= ¥ 7.07  ECG Effective Rate:  Group A: ICER= ¥ 7.81  Group C: ICER= ¥ 35.31 | GDP | / | DSA | Drug prices reduced by 10%. |
| No.88: Lian Tang, 2022 | ICER= ¥ 2,997.56 | 2,100-2,200 (Obtained from published literature) | / | DSA+PSA | Drug cost fluctuate within the range of the highest and lowest prices listed on Yaozhi.com, efficacy rate vary within a 5% margin above and below the point estimate. |
| No.89: ZHANG XinYing, 2024 | ICUR= ¥ -709,471.85/QALY (Mild to moderate patients)  ICUR= ¥ -408,504.73/QALY(severely ill patients) | GDP | / | DSA | Length of hospital stay for different disease subtypes, average daily hospitalization cost, utility values for different health states, and cost of Huashi Baidu Granule. |
| No.90: WANG Xiaoli, 2023 | ICUR= ¥ 3,501,593.41/QALY (Group A vs. Group B)  ICUR= ¥ -1,319,656,80/QALY (Group A vs. Group C) | GDP | / | DSA | Cost fluctuate by 20%, and utility values fluctuate by 0.03. |
| No.91: ZHU Longxun, 2024 | ICER= ¥ 455.79 | / | / | DSA+PSA | The efficiency change range is estimated to fluctuate by 10%, and the drug price range is based on the highest retail prices of drugs in different provinces as found in the Yaozhi.com. |
| No.92: HOU Xiaohua, 2024 | ICUR= ¥ 2,142.75/QALY (Total)  ICUR= ¥ 5.86/QALY (Mild)  ICUR= ¥ 3,741.84/QALY (Moderate)  ICUR= ¥ 249.43/QALY (Severe ) | GDP | / | DSA | Drug prices and efficacy parameters fluctuate by 10%. |
| No.93: LI Junhui, 2025 | ICUR= ¥ 68,181.9/QALY | GDP | / | DSA+PSA | The drug cost is reduced by 10% as its lower price limit, and basic examination fees, efficacy rate, and adverse event rate are set with upper and lower limits based on their 95% CI (if the 95% CI is unknown, the upper and lower limits are set by adjusting the baseline value up and down by 10%). |
| No.94: LIU Chang, 2022 | ICUR= ¥ 60,460.25/QALY | GDP | 5% | DSA+PSA | Treatment duration 30-168 days, medication cost, lost work time cost, labor productivity loss ± 15%, state transition probability ± 5%. |
| No.95: LI Menglan, 2020 | ICER= ¥ 32.38 (Group A vs. Group B)  ICER= ¥ 164.53 (Group B vs. Group C) | / | / | DSA | Drug cost reduced by 5% |
| No.96: ZHANG Huiling, 2020 | / | / | / | DSA | Drug cost decrease by 20%, and time cost decrease by 20%. |
| No.97: Yang Guangyao, 2024 | / | / | / | / | / |
| No.98: JIANG Qiyun, 2025 | ICUR= ¥ -35897.91/QALY | / | / | DSA | Drug cost, other cost excluding medications, including medical service fees, laboratory fees, treatment fees, direct non-medical cost, and indirect cost fluctuate by 20%. |
| No.99: LI Xuejing, 2021 | / | / | / | DSA | The price of drugs was fluctuated by 30%. |
| No.100: WEI Jiayin, 2023 | Chronic Cholecystitis:  ICER= ¥ -0.64 (Group A vs. Group B)  ICER= ¥ 0.31 (Group B vs. Group C)  ICER= ¥ -41.75 (Group A vs. Group C)  Chronic Calculous Cholecystitis:  ICER= ¥ -43.96 (Group A vs. Group B)  ICER= ¥ -0.97 (Group A vs. Group E) | / | / | DSA+PSA | Drug cost fluctuate by 10%, and efficacy parameters fluctuate by 10%. |
| No.101: HE Yumei, 2024 | ICER= ¥ 5,430 | / | / | DSA+PSA | The drug price, dosage, patient compliance, overall efficacy rate, and the upper and lower limits of variable values are assigned specific values. For parameters where the range of variable values cannot be obtained, a 20% variation range is used. |
| No.102: YANG Daowen, 2025 | ICER= ¥ -793.82 (Group B vs. Group A)  ICUR= ¥ -76,386.79/QALY (Group B vs. Group A)  ICER= ¥ 795.17 (Group C vs. Group A)  ICUR= ¥ 30,192.48/QALY (Group C vs. Group A) | GDP (ICER, ICUR) | / | DSA+PSA | The upper and lower limits (95% CI) of key parameters such as drug prices, the number of acute exacerbation of chronic bronchitis, and QALY. |
| No.103: Zou Hong, 2025 | ICER= ¥ 102.28 | / | / | DSA | Drug cost decreased by 10%, while examination cost increased by 5%. |
| No.104: LIU Guoqiang, 2021 | / | / | / | DSA | Drugs cost adjusted by 15%. |
| No.105: LI Kebiao, 2022 | ICER= ¥ 640.29 | Based on patient surveys (¥ 1,506.67) | / | DSA+PSA | The highest and lowest winning bid prices for the drugs, as well as the highest and lowest values of the efficacy data from the two groups, were analyzed. |
| No.106: CUI Xin, 2023 | ICER= ¥ 65.15 | / | / | DSA+PSA | The parameter ranges for the highest and lowest winning bid prices of the drugs, and medication duration, were set to fluctuate by 10% above and below the baseline values, the efficacy parameters for the two intervention schemes were set based on the range of efficacy changes included in the meta-analyses, fluctuating by 5% above and below the baseline values. |
| No.107: HU Yuer, 2023 | ICER= ¥ 6.06 | / | / | DSA+PSA | Two sets of efficacy parameters ±10%, patient medication time (7-10 d), unit price of compound Danshen spray ±10%, patient medication adherence (6-9). |
| No.108: WANG Guozhen, 2020 | ICER= ¥ 1,045.00 | / | / | DSA | Drug prices and efficacy parameters were fluctuate by up to 15%. |
| No.109: WANG Xianying, 2021 | ICER= ¥ 216.42 | / | / | DSA | The winning bid price for the drug will fluctuate between the highest and lowest winning bid prices, with the efficacy parameters fluctuating by 5% above or below the baseline value. |
| No.110: LI Yunhui, 2025 | ICER= ¥ 197.19 | CDI | / | DSA+PSA | Drug prices and clinical effects of the two groups were fluctuated with a range of 5%. |
| No.111: LIU Guoqiang, 2020 | ICER= ¥ 4,987. 93 (Group A vs. Group B)  ICER= ¥ - 5,394. 05 (Group A vs. Group C) | / | / | DSA+PSA | Drug cost ±10%, adverse reaction treatment cost ±20%, and Total effective rate ±15%. |
| No.112: ZHENG Qinxin, 2021 | / | / | / | DSA | Drugs cost adjusted by 10% up or down |
| No.113: LU Yunfei, 2021 | ICER= ¥ 2,626.00 (Group A vs. Group B) | GDP | / | DSA+PSA | Drugs cost, RR value and treatment duration (If real-world data is available for the parameter's range of variation, the real-world data will be used directly, if no real-world source is available for the parameter's range of variation, it will be assumed that the parameter fluctuates within 10% of the baseline value). |
| No.114: ZHOU Ruiyue, 2023 | / | / | / | DSA | Drugs cost adjusted by 20% up or down. |
| No.115: CHEN Yu, 2025 | ICER= ¥ 57.70 | CDI | / | DSA+PSA | Cost parameters fluctuate by 10% of the baseline value, the time to cough resolution and the number of days of medication for patients are set with a fluctuation range of 4 to 14 days, based on the range of cough resolution times observed in individual patients during clinical trials. |
| No.116: DING Liman, 2023 | ICER= ¥ 213.9 (The remission time of clinical symptoms)  ICER= ¥ 149.3 (cure time)  ICER= ¥ 8.2 (cure rate)  ICER= ¥ 295.4 (antipyretic onset time)  ICER= ¥ 114.3 (complete antipyretic time)  ICER= ¥ 29.3 (viral nucleic acid negative rate 3d)  ICER= ¥ 4.9 (viral nucleic acid negative rate 5d)  ICER= ¥15.4 (TCM syndrome curative effect Cure rate 3d)  ICER= ¥ 8.4 (TCM syndrome curative effect Visible efficacy rate 3d)  ICER= ¥ 2.7 (TCM syndrome curative effect Effective Rate 3d)  ICER= ¥ 19.2 (TCM syndrome curative effect Cure rate 5d)  ICER= ¥ 10.8 (TCM syndrome curative effect Visible efficacy rate 5d) | / | / | DSA+PSA | Drug cost and the remission time of clinical symptoms in the Oseltamivir Phosphate Capsule group is derived from the extreme values and 95% CI reported in the literature, all other parameters were adjusted by 20% from their original values. |
| No.117: CHEN Zhongguo, 2023 | ICER= ¥ 19.4 | CDI | / | / | / |
| No.118: QI Ran, 2022 | ICUR= ¥ 781,442.95/QALY (24w)  ICUR= ¥ -2,604,809.84 /QALY (36w)  ICER= ¥ -1,724.8 (36w) | GDP | / | DSA+PSA | The fluctuation range for drug cost is 20%, and the fluctuation range for efficacy parameters is 10%. |
| No.119: LI Jintian, 2022 | ICER= ¥ 444.89 (Group A)  ICER= ¥ -316.00 (Group B)  ICER= ¥ -683.20 (Group C)  ICER= ¥ -1,220.80 (Group D)  ICER= ¥ 4,288.67 (Group E)  ICER= ¥ -71.07 (Group F) | GDP | / | DSA | Treatment course and dosage (within a 95% CI or±20% range). |
| No.120: WANG Fuping, 2024 | ICER= ¥ -170.65 | CDI | / | DSA+PSA | The average cost of MS treatment, the unit price of Kuntian Capsule, and the range of data variation for efficacy parameters under different intervention measures were obtained from literature or databases. The 95% CI of the baseline value or a 20% fluctuation above and below the baseline value was used as the upper and lower limits of the parameters. |
| No.121: WANG Fuping, 2024 | ICER= ¥ 822.46 | CDI | / | DSA+PSA | The unit price of the drug and the effect parameters under different intervention measures use the 95% CI of the baseline value or a 10% fluctuation above and below the baseline value as the upper and lower limits of the parameters. |
| No.112: CUI Xin, 2022 | ICER= ¥ 2,811.36 | CDI | / | DSA+PSA | The average treatment cost, drug unit price, and the range of variation in effect parameters for different interventions were obtained from literature or databases, using the 95% CI of the baseline value or a 20% fluctuation above and below the baseline value. |
| No.123: DU Guiping, 2022 | ICER= ¥ -1,723.28 | / | / | DSA | The cost fluctuates within a range of 20%, and the effect parameter is assumed to fluctuate within a range of 10%. |
| No.124: CUI Xin, 2023 | ICER= ¥ 1078.38 | / | / | DSA+PSA | The parameter ranges for cost and patient medication time were set using a base value ±20%, while the parameter ranges for the efficacy of the two intervention schemes in treating angina were set using a base value ±5%. |
| No.125: ZHAO Dongming, 2024 | Group A: ICER= ¥ -0.56-0.49  Group B: ICER= ¥ 0.001 | / | / | DSA | The parameter ranges for drug prices and clinical efficacy were set to fluctuate within 10% above and below the base value. |
| No.126: WEI Zhongyi, 2024 | ICER= ¥ 11,452.30 (the percentage of forward sperm motility before and after)  ICER= ¥ 4.094.10 (Total effective rate) | CDI | / | DSA+PSA | Drug prices, patient medication adherence, patient medication timing, and efficacy indicators use the 95% CI of the baseline value or a 20% fluctuation above and below that value as the upper and lower limits of the parameters. |
| No.127: ZHANG Yanling, 2025 | ICER= ¥ -1.24 | / | / | DSA | The parameter ranges for drug prices and clinical efficacy were set to fluctuate within 10% above and below the base value. |
| No.128: XU Longchen, 2025 | ICUR= ¥ 35,421.75/QALY (15y)  ICUR= ¥ 34,105.15/QALY (25y) | GDP | / | PSA | / |
| No.129: SHAO Wei, 2025 | ICER= ¥ 96.8 | / | / | DSA+PSA | Drug prices and clinical efficacy are parameters that vary within their respective ranges (minimum value, baseline value, maximum value). |
| No.130: CUI Xin, 2023 | ICER= ¥ 2,867.26 | / | / | DSA+PSA | Patient medication adherence (2-3 pills), number of days of medication taken by the patient (±10%), unit price of medication in the QC group (±10%), treatment cost of the control group (±10%), and efficacy parameters of both groups (±5%). |
| No.131: ZHANG Xuebin, 2021 | ICUR= ¥33,165.74 | GDP | / | DSA | Drugs cost, other cost adjusted by 10% up or down |
| No.132: HU Jing, 2020 | / | / | 8% | DSA | Drug cost reduced by 10%, Bed cost, treatment cost, and nursing cost each increased by 5%. |
| No.133: LIU GuoQiang, 2020 | / | / | / | DSA | Drug cost reduced by10% |
| No.134: LI Xuejing, 2020 | / | / | / | DSA | The range of price fluctuations for the drug is defined by the highest and lowest winning bid prices for drug announced on the yaozhi.com in 2019. |
| No.135: YU Rong, 2023 | ICER= ¥ -14.82 (in China Nationwide)  ICER= ¥ 35.08 (in Gansu province)  ICER= ¥ 46.35 (in Ningxia, Shandong province)  ICER= ¥ 84.00 (in Jiangxi province) | / | / | / | / |
| No.136: LIU Fumei, 2021 | ICER= ¥ 118 | CDI |  | DSA | Unit price of drug, patient medication adherence, number of days of medication use, average cost of acute cerebral infarction treatment, efficacy parameters, etc. |
| No.137: CHEN Binbin, 2022 | ICUR= ¥ 174,136.62/QALY | GDP | 5% | DSA+PSA | Discount rate (0~10%), utility parameter (±10%), cost parameter (20% reduction in drug cost), treatment discontinuation rate and disease progression probability (±20%). |
| No.138: CUI Peng, 2022 | ICER= ¥ 104.72 | / | / | / | / |
| No.139: GUAN Haijing, 2022 | ICUR= ¥ 117,861/QALY | GDP | / | DSA | For efficacy parameters, RR value, drug cost, hospitalization cost, utility value, hospitalization rate, mortality rate, and length of hospital stay, the 95% CI of the baseline value or a 20% fluctuation above and below the baseline value were used as the upper and lower limits of the parameters. |
| No.140: LIN Sisi, 2024 | ICUR= ¥ 77,002.17/QALY (life years)  ICUR= ¥ 33,421.84/QALY (10y) | GDP | 5% | DSA+PSA | Treatment efficacy parameters, patient compliance, number of days of medication, drug cost and utility value. |
| No.141: PENG Nan, 2024 | ICER= ¥ -2,180  ICUR= ¥ 51,375/QALY | GDP (ICUR) | / | / | / |
| No.142: LEI Xiang, 2020 | ICER= ¥ 2,299.06 (DAS28 score)  ICER= ¥ 623.78 (Number of tender joints)  ICER= ¥ 462.45(Number of swollen joints)  ICER= ¥ 171.57(Overall health assessment of the subject)  ICER= ¥ 63.56 (ACR20)  ICER= ¥ 68.57 (ACR50)  ICER= ¥ 216.31 (Pain assessment of the subject)  ICER= ¥ 153.86 (Overall assessment of disease activity of the subject)  ICER= ¥ 148.19 (Overall assessment of disease activity of the physician)  ICER= ¥ 957.94 (HAQ) | GDP | / | DSA | Drug prices and efficacy parameters fluctuate by 10%. |
| No.143: WANG Yulai, 2021 | Acute cerebral infarction:  ICER= ¥ 10.01 (Group A vs. Group B)  ICER= ¥ 121.66 (Group A vs. Group C)  ICER= ¥ 192.46 (Group A vs. Group D)  Posterior circulation ischemic vertigo:  ICER= ¥ 5.56 (Group A vs. Group B)  Basilar artery insufficiency:  ICER= ¥ 146.65 (Group A vs. Group B)  ICER= ¥ 1.07 (Group A vs. Group D) | / | / | DSA | The range of variation in drug efficacy is given by the 95% CI obtained from the meta-analyses, and the range of variation in drug price is a fluctuation of 10% from the original data. |
| No.144: HE Yumei, 2024 | ICER= ¥ 5,223.00 | / | / | DSA+PSA | The drug price, dosage, patient adherence, Total effective rate, and upper and lower limits of variable values are assigned specific values. For parameters where the range of variable values cannot be obtained, a variation range of 20% is used. |
| No.145: XIANG Heng, 2025 | ICER= ¥ 1,893.97 | According to the 2022 China Health Statistics Yearbook, the average medical cost for fracture treatment in public hospitals, which is ¥ 21,074.50, was used as the WTP threshold. | / | DSA+PSA | The parameter ranges for drug prices, clinical efficacy rate, and adverse event rate are within a 10% fluctuation range above and below the original data. |
| No.146: XU Manling, 2025 | ICER= ¥ -31.17 | / | / | DSA | The parameter ranges for drug prices, clinical efficacy rate are within a 10% fluctuation range above and below the original data. |
| No.147: CAI Jiarui, 2025 | ICER= ¥ 19.51 | CDI | / | DSA | The parameter ranges for drug prices, clinical efficacy rate are within a 10% fluctuation range above and below the original data. |
| No.148: HOU Hairuo, 2023 | / | / | / | DSA | Cost were fluctuated by 15%. |
| No.149: WANG Xin, 2024 | ICER= ¥ -43.55 (Group A)  ICER= ¥ -51.47 (Group B) | / | / | DSA | The parameter ranges for drug prices, clinical efficacy rate are within a 10% fluctuation range above and below the original data. |
| No.150: Zhang Hongyan, 2025 | ICER= ¥ 277.015 | CDI | / | DSA+PSA | The drug unit price, patient medication adherence, number of days of medication, average cost of headache treatment, and efficacy parameters for both groups will use a variation range of 20% of the baseline values. |
| No.151: HUANG Zhengming, 2025 | ICER= ¥ -39.24 | / | / | DSA | The parameter ranges for drug prices, clinical effective rate are within a 10% fluctuation range above and below the original data. |
| No.152: XU Qian, 2022 | / | / | / | / | / |
| No.153: LUO Ji, 2021 | / | / | / | DSA | Cost were fluctuate by 10%. |
| No.154: ZHANG Xuebin, 2021 | Total effective rate of angina pectoris:  ICER= ¥ -29.44 (Group A vs. Group B)  ICER= ¥ -141.72 (Group B vs. Group C)  VAS score:  ICER= ¥ -407.23 (Group A vs. Group B)  ICER= ¥ -1118.04 (Group B vs. Group C) | / | / | DSA | Drug cost increased by 10%. |
| No.155: ZHENG Hang, 2023 | ICER= ¥ 58.3 (Group A)  ICER= ¥ 47.7 (Group B)  ICER= ¥ 31.6 (Group C)  ICER= ¥ 44.5 (Group D)  ICER= ¥ 21.6 (Group E)  ICER= ¥ 56.9 (Group F) | GDP | / | DSA | Drug cost fluctuated by 20%. |
| No.156: PANG Hongbo, 2022 | / | / | / | DSA | Drug cost decreased by 20%. |
| No.157: YANG Chunmei, 2020 | ICUR= ¥ 135.8/QALY | The Institute for Clinical and Economic Review recommends an ICUR threshold of $50,000 to $150,000/QALY. | / | / | / |
| No.158: Zhou Bin, 2023 | / | / | / | / | / |
| No.159: LI Chang, 2024 | Clinical Effective Rate:  ICER= ¥ -211.5 (Nationwide in China)  ICER= ¥ -4,580 (Guangxi, Chongqin, Guangdong, Heibei province)  ICER= ¥ -212.75 (Heilongjiang, Hunan, Yunnan province)  ICER= ¥ -211.50 (Jiangsu, Guizhou, Hubei, Jilin, Liaoning, Inner Mongolia, Shanxi, Xinjiang, Shanghai, Hennan, Zhejiang)  ICER= ¥ -102.75 (Ningxia)  ICER= ¥ 0.50 (Gansu)  ICER= ¥ 48.50 (Shandong)  ECG Effective Rate:  ICER= ¥ -40.29 (Nationwide in China)  ICER= ¥ -872.38 (Guangxi, Chongqin, Guangdong, Heibei province)  ICER= ¥ -40.52 (Heilongjiang, Hunan, Yunnan province)  ICER= ¥ -40.29 (Jiangsu, Guizhou, Hubei, Jilin, Liaoning, Inner Mongolia, Shanxi, Xinjiang, Shanghai, Hennan, Zhejiang)  ICER= ¥ -19.57 (Ningxia)  ICER= ¥ 0.10 (Gansu)  ICER= ¥ 9.24 (Shandong) | / | / | / | / |
| No.160: WANG Yizhu, 2024 | ICER= ¥ 3,046.91 | CDI | / | DSA+PSA | The baseline values, minimum and maximum values for the unit prices of the two drugs were determined based on multiple variables, including the winning bid prices from Menet.com,and the baseline values for the efficacy parameters of the two groups were based on a meta-analyses, and a 20% fluctuation above and below the baseline value was used as the upper and lower limits for the efficacy parameters. |
| No.161: WANG XiaoYan, 2024 | ICER= ¥ 2,324.06 | CDI | / | DSA | Drug unit price, patient medication adherence, number of days of medication, average cost of treatment, efficacy parameters. |
| No.162: DING Yanbing, 2025 | ICER= ¥ 7.01 (2w)  ICER= ¥ 11.09 (more than 2 w) | CDI | / | DSA | The parameter ranges for drug prices, clinical efficacy Rate are within a 10% fluctuation range above and below the original data. |
| No.163: REN Huijun, 2024 | ICER= ¥ -173.29 | / | 5% | DSA | Drug prices fluctuate between the highest and lowest winning bid prices in China. |
| No.164: MA Haiqing, 2021 | ICER= ¥ 2.04 (Group A vs. Group C)  ICER= ¥ 5.06 (Group B vs. Group C) | / | / | / | / |
| No.165: HUANG Qian, 2020 | ICER= ¥ 199.33 | / | / | / | / |
| No.166: ZHANG Xueyi, 2021 | ICER= ¥ 383.88 | / | / | / | / |
| No.167: LI Na, 2021 | ICER= ¥ 337.90 | / | / | / | / |
| No.168: JIA Jianying, 2023 | / | / | / | / | / |
| No.169: LIU Yu, 2020 | ICER= ¥ 111.75 (Group A vs. Group B) | / | / | DSA | The prices of three injections were decreased by 15%. |
| No.170: ZHU Jingyan, 2020 | / | / | / | / | / |
| No.171: HUANG Haitao, 2022 | ICER= ¥ -10,389.61 | / | / | DSA | Drug prices fluctuate between the highest and lowest winning bid prices in China. |
| No.172: WANG Xingdong, 2021 | / | / | / | / | / |
| No.173: SONG Miaoyuan, 2022 | / | / | / | DSA | Drug cost decreased by 35%, examination and treatment cost increased by 25%, and hospitalization cost increased by 10%. |
| No.174: WANG Xiu, 2023 | ICUR= ¥ 60,460.25/QALY | GDP | 5% | DSA+PSA | Treatment duration: 30–168 days, drug cost, lost work time cost, and labor productivity losses were subject to a ±15% variation, state transition probabilities are subject to a ±5% variation. |
| No.175: PAN Zhehao, 2023 | / | / | / | / | / |
| No.176: Feng Rongwei, 2020 | ICER= ¥ - 76.33 (Group B vs. Group A)  ICER= ¥ 145.09 (Group C vs. Group A)  ICER= ¥ - 77.41 (Group D vs. Group A)  ICER= ¥ - 6.68 (Group E vs. Group A) | / | / | / | / |
| No.177: LI Meifang, 2024 | ICER= ¥ 8.94 (Total effective rate)  ICER= ¥ 10.06 (Total effective rate of TCM syndromes) | Based on the assumption of a cost-effectiveness threshold of ¥100 per unit in published economic studies. | / | / | / |
| No.178: YANG Qian, 2024 | ICER= ¥ 14.48 (Total effective rate)  ICER= ¥ -507.28 (Upper abdominal distension score)  ICER= ¥ -310 (Belching score)  ICER= ¥ -744 (Loss of appetite score)  ICER= ¥ -656.47 (Epigastric pain score) | CDI | / | DSA | The parameter ranges for drug prices, efficacy rate are within a 10% fluctuation range above and below the original data. |
| No.179: LU Yunfei, 2021 | / | / | / | / | / |
| No.180: CHEN Yi-peng  et al. 2021 | ICER= ¥ 12.90 | / | / | DSA | / |
| No.181: LIU Zhihui, 2022 | / | / | / | DSA | Drug cost decreased by 35%. |
| No.182: XU Lingjun, 2022 | / | / | / | / | / |
| No.183: HUA Xiangxiang, 2021 | ICER= ¥ 576.57 | / | / | / | / |
| No.184: LIU Huan, 2020 | ICER= ¥ 23.39 | 100 (WTP threshold determined through expert interviews) | / | DSA+PSA | / |
| No.185: CHEN Wenwen, 2024 | ICER= ¥ 0.4983 | GDP | / | DSA | Drug cost decrease by 10%, while efficacy parameters increase by 10% or decrease by 5%. |
| No.186: WANG Zhijun, 2024 | Clinical Effective Rate:  ICER= ¥ -285.26 (Nationwide in China)  ICER= ¥ -306.60 (Jilin province)  ICER= ¥ -285.26 (Chongqing, Liaoning, Inner Mongolia,, Shanxi, Xinjiang, Jiangsu, Yunnan, Shanghai, Henan, Hebei, Guangdong province)  ICER= ¥ -217.22 (Gansu province)  ICER= ¥ -207.98 (Sichuan, Zhejiang, Guizhou province)  ICER= ¥ -207.48 (Guangxi, Hubei province)  ICER= ¥ -184.63 (Ningxia province)  ICER= ¥ -181.27 (Shandong province)  ICER= ¥ -177.24 (Jiangxi province)  ECG Effective Rate:  ICER= ¥ -570.53 (Nationwide in China)  ICER= ¥ -613.20 (Jilin province)  ICER= ¥ -570.53 (Chongqing, Liaoning, Inner Mongolia,, Shanxi, Xinjiang, Jiangsu, Yunnan, Shanghai, Henan, Hebei, Guangdong province)  ICER= ¥ -434.45 (Gansu province)  ICER= ¥ -415.97 (Sichuan, Zhejiang, Guizhou province)  ICER= ¥ -414.96 (Guangxi, Hubei province)  ICER= ¥ -369.26 (Ningxia province)  ICER= ¥ -362.54 (Shandong province)  ICER= ¥ -354.48 (Jiangxi province) | / | / | / | / |
| No.187: QU Detao, 2023 | / | / | / | / | / |
| No.188: XIONG Liping, 2020 | / | / | / | / | / |
| No.189: ZHANG Yaxuan, 2022 | Group A:  ICER= ¥ 2.16 (< 1y infant)  ICER= ¥ 4.32 (1-3y infant)  Group B:  ICER= ¥ 1.03 (3-6m infant)  ICER= ¥ 1.37 (7m-1y infant)  ICER= ¥ 2.06 (1-3y infant) | GDP | / | / | / |
| No.190: WANG Qingqing, 2025 | ICER= ¥ 36.66 (Vertigo)  ICER= ¥ 35.97 (Hypertension) | / | / | / | / |
| No.191: XU Huanxiang, et al.2020 | / | / | / | / | / |
| No.192: WANG FENG, et al.2021 | / | / | / | / | / |
| No.193: Zuo Kaini, 2020 | Group A:  ICER= ¥ 20.43 (1 month)  ICER= ¥ 16.55 (2 month)  ICER= ¥ 15.56 (3 month)  ICER= ¥ 28.21 (6 month)  Group B:  ICER= ¥ 2.43 (1 month) | / | / | DSA | Drug cost decreased by 10%. |
| No.194: ZHANG Yaxuan, 2022 | ICER= ¥ 37.58 (Group A)  ICER= ¥ 17.85 (Group B)  ICER= ¥ 19.38 (Group C) | / | / | / | / |
| No.195: TAN Shurao, 2021 | / | / | / | / | / |
| No.196: HUANG Shaohua, 2025 | / | / | / | / | / |
| No.197: ZHANG He, 2025 | ICER= ¥ -5.56 | / | / | / | / |
| No.198: LV Xiaoqin, 2020 | / | / | / | DSA | Drug cost and the efficacy rate decreased by 10%. |

ICER: Incremental Cost-Effectiveness Ratio, ICUR: Incremental Cost-Utility Ratio, GDP: Gross Domestic Product, CDI: Capita Disposable income, WTP: Willingness to Pay, DSA: Deterministic Sensitivity Analyses, PSA: Probabilistic Sensitivity Analyses.

**Table S4. Subgroup and Sensitivity Analyses.**

| **Category** | **Group** | **N** | **CHEERS**  **(Mean±SD)** | **BMJ**  **(Mean±SD)** | **QHES**  **(Mean±SD)** | **ICC** | **C vs BMJ**  **MD (95% LoA)** | **C vs QHES**  **MD (95% LoA)** | | **BMJ vs QHES**  **MD (95% LoA)** | |
| --- | --- | --- | --- | --- | --- | --- | --- | --- | --- | --- | --- |
| **Subgroup: Language** | Chinese | 178 | 52.77±8.46 | 70.67±9.24 | 67.93±14.27 | 0.571 | -17.94 (-32.12, 3.75) | -15.15 (-39.04, 8.74) | 2.78 (-18.16, 23.72) | |  |
|  | English | 20 | 68.58±5.79 | 74.02±10.78 | 76.50±16.52 | 0.588 | -8.06 (-25.85, 9.74) | -10.54(-38.73, 17.65) | -2.48 (-17.44, 12.47) | |  |
| **Subgroup: Database** | CNKI | 172 | 53.16±8.46 | 71.12±9.04 | 68.66±14.19 | 0.556 | -17.96 (-31.99, -3.94) | -15.50 (-39.49, 8.49) | 2.47 (-18.44, 23.37) | |  |
|  | WF | 174 | 53.11±8.48 | 70.83±9.41 | 68.50±14.51 | 0.555 | -17.73 (-32.89, -2.56) | -15.39 (-39.95, 9.17) | 2.33 (-18.53, 23.20) | |  |
|  | VIP | 189 | 54.47±8.96 | 71.51±9.07 | 69.45±14.46 | 0.552 | -17.04 (-32.75, -1.33) | -14.98 (-39.63, 9.66) | 2.06 (-18.55, 22.66) | |  |
|  | PubMed | 30 | 62.87±7.94 | 74.09±9.22 | 72.23±16.67 | 0.423 | -11.22 (-32.29, 9.85) | -9.36 (-39.55, 20.83) | 1.86 (-21.22, 24.94) | |  |
|  | Embase | 28 | 62.79±8.15 | 74.27±9.52 | 73.25±16.77 | 0.441 | -11.48 (-33.06, 10.11) | -10.46 (-40.27, 19.36) | 1.02 (-21.94, 23.98) | |  |
|  | Cochrane Library | 7 | 56.40±9.54 | 71.42±9.51 | 68.86±13.21 | 0.486 | -15.02 (-41.31, 11.27) | -12.46 (-33.05, 8.13) | 2.56 (-14.52, 19.65) | |  |
| **Sensitivity Analyses** | Original Dataset | 198 | 54.14±9.16 | 71.08±9.41 | 68.83±14.69 | 0.580 | -16.94 (-32.61, -1.27) | -14.69 (-39.12, 9.75) | 2.25 (-18.37, 22.87) | |  |
|  | Excluded (n=7)* | 191 | 54.29±9.15 | 71.18±9.39 | 69.49±14.42 | 0.581 | -16.89 (-32.39, -1.39) | -15.20 (-39.37, 8.97) | 1.69 (-18.31, 21.69) | |  |
|  | P value | - | 0.87 | 0.92 | 0.65 | - | - |  |  | |  |

***** Excluding 7 studies with undefined TCM drug interventions.

**Table S5. Compliance Rate for CHEERS 2022 Checklist Items.**

|  | **Evaluation Item** | **Yes N(%)** | **Partially N(%)** | **No, N/A N(%)** | **Compliance Rate (%) (Mean±SD)** |
| --- | --- | --- | --- | --- | --- |
| **Title** | Item 1: Identify the study as an economic evaluation and specify the interventions being compared. | 99(50.00) | 98(49.49) | 1(0.51) | 74.75±25.56 |
| **Abstract** | Item 2: Provide a structured summary that highlights context, key methods, results, and alternative analyses. | 180(90.91) | 10(5.05) | 8(4.04) | 93.43±22.13 |
| **Introduction** | Item 3: Give the context for the study, the study question, and its practical relevance for decision making in policy or practice. | 150(75.76) | 48(24.24) | 0 | 87.88±21.48 |
| **Method** | Item 4: Indicate whether a health economic analyses plan was developed and where available. | 3(1.52) | 6(3.03) | 189(95.45) | 3.03±14.81 |
|  | Item 5: Describe characteristics of the study population (such as age range, demographics, socioeconomic, or clinical characteristics). | 62(31.31) | 55(27.78) | 81(40.91) | 45.20±42.33 |
|  | Item 6: Provide relevant contextual information that may influence findings. | 8(4.04) | 62(31.31) | 128(64.65) | 19.70±28.34 |
|  | Item 7: Describe the interventions or strategies being compared and why chosen. | 76(38.38) | 121(61.11) | 1(0.51) | 68.94±24.83 |
|  | Item 8: State the perspective(s) adopted by the study and why chosen. | 22(11.11) | 116(58.59) | 60(30.30) | 40.40±30.79 |
|  | Item 9: State the time horizon for the study and why appropriate. | 41(20.71) | 140(70.71) | 17(8.59) | 56.06±26.44 |
|  | Item 10*: Report the discount rate(s) and reason chosen. | 19(9.60) | 12(6.06) | 2(1.01)+165(83.33) | 75.76±30.93 |
|  | Item 11: Describe what Outcome were used as the measure(s) of benefit(s) and harm(s). | 191(96.46) | 7(3.54) | 0 | 98.23±9.26 |
|  | Item 12: Describe how Outcome used to capture benefit(s) and harm(s) were measured. | 146(73.74) | 20(10.10) | 32(16.16) | 78.79±37.76 |
|  | Item 13: Describe the population and methods used to measure and value Outcome. | 122(61.62) | 27(13.64) | 49(24.75) | 68.43±42.76 |
|  | Item 14: Describe how Cost were valued. | 163(82.32) | 32(16.16) | 3(1.52) | 90.40±21.58 |
|  | Item 15: Report the dates of the estimated resource quantities and unit Cost, plus the currency and year of conversion. | 61(30.81) | 39(19.70) | 98(49.49) | 40.66±43.93 |
|  | Item 16*: If modelling is used, describe in detail and why used. Report if the model is publicly available and where it can be accessed. | 63(31.82) | 44(22.22) | 1(0.51)+90(45.45) | 78.70±25.76 |
|  | Item 17*: Describe any methods for analyzing or statistically transforming data, any extrapolation methods, and approaches for validating any model used. | 29(14.65) | 35(17.68) | 44(22.22)+90(45.45) | 43.06±40.71 |
|  | Item 18: Describe any methods used for estimating how the results of the study vary for subgroups. | 15(7.58) | 4(2.02) | 179(90.40) | 8.59±27.17 |
|  | Item 19: Describe how impacts are distributed across different individuals or adjustments made to reflect priority populations. | 2(0.99) | 0 | 196(98.99) | 1.01±10.02 |
|  | Item 20: Describe methods to characterize any sources of uncertainty in the analyses. | 147(74.24) | 14(7.07) | 37(18.69) | 77.78±39.49 |
|  | Item 21: Describe any approaches to engage patients or service recipients, the general public, communities, or stakeholders (such as clinicians or payers) in the design of the study. | 9(4.55) | 3(1.52) | 186(93.94) | 5.30±21.60 |
| **Result** | Item 22: Report all analytic inputs (such as values, ranges, references) including uncertainty or distributional assumptions. | 144(72.73) | 49(24.75) | 5(2.53) | 85.10±25.54 |
|  | Item 23: Report the mean values for the main categories of Cost and Outcome of interest and summarize them in the most appropriate overall measure. | 175(88.38) | 23(11.62) | 0 | 94.19±16.06 |
|  | Item 24: Describe how uncertainty about analytic judgments, inputs, or projections affect findings. Report the effect of choice of discount rate and time horizon, if applicable. | 117(59.09) | 44(22.22) | 37(18.69) | 70.20±39.30 |
|  | Item 25: Report on any difference patient/service recipient, general public, community, or stakeholder involvement made to the approach or findings of the study. | 3(1.52) | 1(0.51) | 194(97.98) | 1.77±12.72 |
|  | Item 26: Report key findings, limitations, ethical or equity considerations not captured, and how these could affect patients, policy, or practice. | 154(77.78) | 40(20.20) | 4(2.02) | 87.88±23.72 |
| **Other Relevant Information** | Item 27: Describe how the study was funded and any role of the funder in the identification, design, conduct, and reporting of the analyses. | 20(10.10) | 81(40.91) | 97(48.99) | 30.56±33.24 |
|  | Item 28: Report authors conflicts of interest according to journal or International Committee of Medical Journal Editors requirements. | 38(19.19) | 0 | 160(80.81) | 19.19±39.48 |
| **Total (%)**  **(Mean±SD, Range)** | 54.14%±9.16% (28.00%~76.79%) | | | | |

Items marked with "*" have applicable conditions.

**Table S6. Compliance Rate for the BMJ Guidelines Items.**

|  | **Evaluation Item** | **Yes N(%)** | **Partially N(%)** | **No, N/A N(%)** | **Compliance Rate (%) (Mean±SD)** |
| --- | --- | --- | --- | --- | --- |
| **Study Design** |  |  |  |  |  |
| **Study Question** | Item 1: The research question is stated | 190(95.96) | 8(4.04) | 0 | 97.98±9.87 |
|  | Item 2: The economic importance of the research question is stated | 171(86.36) | 16(8.08) | 11(5.56) | 90.40±25.86 |
| **Comparative Approach Selection** | Item 3: The viewpoint(s) of the analyses are clearly stated and justified | 128(64.65) | 11(5.56) | 59(29.80) | 67.42±45.47 |
|  | Item 4: The rationale for choosing the alternative programmes or interventions compared is stated | 80(40.40) | 118(59.60) | 0 | 70.20±24.50 |
|  | Item 5: The alternatives being compared are clearly described | 119(60.10) | 35(17.68) | 44(22.22) | 68.94±41.33 |
| **Evaluation Method** | Item 6: The form of economic evaluation used is stated | 195(98.48) | 2(1.01) | 1(0.51) | 98.99±8.67 |
|  | Item 7: The choice of form of economic evaluation is justified in relation to the questions addressed | 104(52.53) | 27(13.64) | 67(33.84) | 59.34±45.63 |
| **Data Collection** |  |  |  |  |  |
| **Data Validity** | Item 8: The source(s) of effectiveness estimates used are stated | 195(98.48) | 0 | 3(1.52) | 98.48±12.25 |
|  | Item 9*: Details of the design and results of effectiveness study are given (if based on a single study) | 70(35.35) | 28(14.14) | 12(6.06)+88(44.44) | 76.36±34.34 |
|  | Item 10*: ) Details of the method of synthesis or meta-analyses of estimates are given (if based on an overview of a number of effectiveness studies) | 64(32.32) | 8(4.04) | 16(8.08)+110(55.56) | 77.27±39.33 |
| **Measurement and Evaluation of Benefit** | Item 11: The primary Outcome measure(s) for the economic evaluation are clearly stated | 194(97.98) | 4(2.02) | 0 | 98.99±7.05 |
|  | Item 12: Methods to value health states and other benefits are stated | 52(26.26) | 21(10.61) | 125(63.13) | 31.57±43.64 |
|  | Item 13: Details of the subjects from whom valuations were obtained are given | 71(35.86) | 47(23.74) | 81(40.91) | 47.73±43.72 |
| **Cost** | Item 14*: ) Productivity changes (if included) are reported separately | 21(10.61) | 1(0.51) | 0+176(88.89) | 97.73±10.66 |
|  | Item 15: The relevance of productivity changes to the study question is discussed | 13(6.57) | 7(3.54) | 178(89.90) | 8.33±26.06 |
|  | Item 16: Quantities of resources are reported separately from their unit Cost | 64(32.32) | 14(7.07) | 120(60.61) | 35.86±46.20 |
|  | Item 17: Methods for the estimation of quantities and unit Cost are described | 127(64.14) | 34(17.17) | 37(18.69) | 72.73±39.52 |
|  | Item 18: Currency and price data are recorded | 154(77.78) | 16(8.08) | 28(14.14) | 81.82±35.95 |
|  | Item 19: Details of currency of price adjustments for inflation or currency conversion are given | 4(1.98) | 13(6.44) | 185(91.58) | 5.30±18.43 |
| **Model** | Item 20*: Details of any model used are given | 85(42.93) | 22(11.11) | 1(0.51)+90(45.45) | 88.89±21.97 |
|  | Item 21*: The choice of model used and the key parameters on which it is based are justified | 40(20.20) | 57(28.79) | 11(5.56)+90(45.45) | 63.43±31.77 |
| **Analyses and Interpretation of Results** |  |  |  |  |  |
| **Cost and Benefit Adjustment** | Item 22: Time horizon of Cost and benefits is stated | 154(77.78) | 17(8.59) | 27(13.64) | 82.07±35.54 |
|  | Item 23*: The discount rate(s) is stated | 31(15.66) | 0 | 2(1.01)+165(83.33) | 93.94±24.23 |
|  | Item 24*: The choice of rate(s) is justified | 23(11.62) | 1(0.51) | 9(4.55)+165(83.33) | 71.21±45.12 |
|  | Item 25*: An explanation is given if Cost or benefits are not discounted | 44(22.22) | 0 | 118(59.60)+33(16.67) | 28.48±45.27 |
| **Acceptability and Uncertainty** | Item 26: Details of statistical tests and confidence intervals are given for stochastic data | 136(68.69) | 22(11.11) | 40(20.20) | 74.24±40.53 |
|  | Item 27: The approach to sensitivity analyses is given | 156(78.79) | 5(2.53) | 37(18.69) | 80.05±39.26 |
|  | Item 28: The choice of variables for sensitivity analyses is justified | 81(40.91) | 68(34.34) | 49(24.75) | 58.08±39.80 |
|  | Item 29: The ranges over which the variables are varied are stated | 147(74.24) | 3(1.52) | 48(24.24) | 75.00±42.97 |
|  | Item 30: Relevant alternatives are compared | 198(100.00) | 0 | 0 | 100.00±0.00 |
|  | Item 31: Incremental analyses is reported | 155(78.28) | 0 | 43(21.72) | 78.28±41.34 |
| **Reporting of Result** | Item 32: Major Outcome are presented in a dissaggregated as well as aggregated form | 192(96.97) | 5(2.53) |  | 98.23±10.54 |
|  | Item 33: The answer to the study question is given | 197(99.49) | 1(0.51) | 0 | 99.75±3.55 |
|  | Item 34: Conclusions follow from the data reported | 198(100.00) | 0 | 0 | 100.00±0.00 |
|  | Item 35: Conclusions are accompanied by the appropriate caveats | 123(62.12) | 0 | 75(37.88) | 62.12±48.63 |
| **Total (%)**  **(Mean±SD, Range)** | 71.08%±9.41% (45.00%~93.75%) | | | | |

Items marked with "*" have applicable conditions.

**Table S7. Scores and Compliance Rate for QHES Scale Items.**

| **Evaluation Item** | **Yes N(%)** | **No N(%)** | **Item Average Score**  **(Mean±SD)** |
| --- | --- | --- | --- |
| Item 1: Was the study objective presented in a clear, specific, and measurable manner? | 181(91.41) | 17(8.59) | 6.34±2.05 |
| Item 2: Were the perspective of the analyses (societal, third-party payer, etc) and reasons for its selection stated? | 140(70.71) | 58(29.29) | 2.81±1.83 |
| Item 3: Were variable estimates used in the analyses from the best available source (i.e., randomized control trial-best, expert opinion-worst)? | 80(40.40) | 118(59.60) | 3.21±3.93 |
| Item 4: If estimates came from a subgroup analyses, were the groups prespecified at the beginning of the study? | 19(9.60) | 179(90.40) | 0.10±0.30 |
| Item 5: Was uncertainty handled by (1) statistical analyses to address random events, (2) sensitivity analyses to cover a range of assumptions? | 140(70.71) | 58(29.29) | 6.42±4.08 |
| Item 6: Was incremental analyses performed between alternatives for resources and Cost? | 155(78.28) | 43(21.72) | 4.66±2.50 |
| Item 7: Was the methodology for data abstraction (including the value of health states and other benefits) stated? | 190(95.96) | 8(4.04) | 4.80±0.98 |
| Item 8: Did the analytic horizon allow time for all relevant and important Outcome? Were benefits and Cost that went beyond 1 year discounted (3% to 5%) and justification given for the discount rate? | 114(57.58) | 84(42.42) | 4.05±3.46 |
| Item 9: Was the measurement of Cost appropriate and the methodology for the estimation of quantities and unit Cost clearly described? | 94(47.47) | 104(52.53) | 3.84±4.01 |
| Item 10: Were the primary Outcome measure(s) for the economic evaluation clearly stated and did they include the major short-term was justification given for the measures/scales used? | 177(89.39) | 21(10.61) | 5.38±1.84 |
| Item 11: Were the health Outcome measures/scales valid and reliable? If previously tested valid and reliable measures were not available, was justification given for the measures/scales used? | 138(69.70) | 60(30.30) | 4.92±3.21 |
| Item 12: Were the economic model (including structure), study methods and analyses, and the components of the numerator and denominator displayed in a clear transparent manner? | 141(71.21) | 57(28.79) | 5.62±3.66 |
| Item 13: Were the choice of economic model, main assumptions, and limitations of the study stated and justified? | 73(36.87) | 125(63.13) | 2.56±3.38 |
| Item 14: Did the author(s) explicitly discuss direction and magnitude of potential biases? | 155(78.28) | 43(21.72) | 4.66±2.50 |
| Item 15: Were the conclusions/recommendations of the study justified and based on the study results? | 194(97.98) | 4(2.02) | 7.84±1.12 |
| Item 16: Was there a statement disclosing the source of funding for the study? | 101(51.01) | 97(48.99) | 1.56±1.50 |
| **Total** (Mean±SD, Range) | 68.83±14.69 (27.00~99.00) | | |

Items marked with "*" have applicable conditions.
